# Targeting c‐Myc transactivation by LMNA inhibits tRNA processing essential for malate‐aspartate shuttle and tumour progression

**DOI:** 10.1002/ctm2.1680

**Published:** 2024-05-20

**Authors:** Jianqun Wang, Mei Hong, Yang Cheng, Xiaojing Wang, Dan Li, Guo Chen, Banghe Bao, Jiyu Song, Xinyi Du, Chunhui Yang, Liduan Zheng, Qiangsong Tong

**Affiliations:** ^1^ Department of Pediatric Surgery Union Hospital Tongji Medical College Huazhong University of Science and Technology Wuhan Hubei Province P. R. China; ^2^ Department of Geriatrics Union Hospital, Tongji Medical College, Huazhong University of Science and Technology Wuhan Hubei Province China; ^3^ Department of Pathology Union Hospital Tongji Medical College Huazhong University of Science and Technology Wuhan Hubei Province P. R. China

**Keywords:** c‐Myc, lamin A, malate‐aspartate shuttle, neuroblastoma, transfer RNA processing, tumour progression

## Abstract

**Background:**

A series of studies have demonstrated the emerging involvement of transfer RNA (tRNA) processing during the progression of tumours. Nevertheless, the roles and regulating mechanisms of tRNA processing genes in neuroblastoma (NB), the prevalent malignant tumour outside the brain in children, are yet unknown.

**Methods:**

Analysis of multi‐omics results was conducted to identify crucial regulators of downstream tRNA processing genes. Co‐immunoprecipitation and mass spectrometry methods were utilised to measure interaction between proteins. The impact of transcriptional regulators on expression of downstream genes was measured by dual‐luciferase reporter, chromatin immunoprecipitation, western blotting and real‐time quantitative reverse transcription‐polymerase chain reaction (RT‐PCR) methods. Studies have been conducted to reveal impact and mechanisms of transcriptional regulators on biological processes of NB. Survival differences were analysed using the log‐rank test.

**Results:**

c‐Myc was identified as a transcription factor driving tRNA processing gene expression and subsequent malate‐aspartate shuttle (MAS) in NB cells. Mechanistically, c‐Myc directly promoted the expression of glutamyl‐prolyl‐tRNA synthetase (*EPRS*) and leucyl‐tRNA synthetase (*LARS*), resulting in translational up‐regulation of glutamic‐oxaloacetic transaminase 1 (GOT1) as well as malate dehydrogenase 1 (MDH1) via inhibiting general control nonrepressed 2 or activating mechanistic target of rapamycin signalling. Meanwhile, lamin A (LMNA) inhibited c‐Myc transactivation via physical interaction, leading to suppression of MAS, aerobic glycolysis, tumourigenesis and aggressiveness. Pre‐clinically, lobeline was discovered as a LMNA‐binding compound to facilitate its interaction with c‐Myc, which inhibited aminoacyl‐tRNA synthetase expression, MAS and tumour progression of NB, as well as growth of organoid derived from *c‐Myc* knock‐in mice. Low levels of *LMNA* or elevated expression of *c‐Myc*, *EPRS*, *LARS*, *GOT1* or *MDH1* were linked to a worse outcome and a shorter survival time of clinical NB patients.

**Conclusions:**

These results suggest that targeting c‐Myc transactivation by LMNA inhibits tRNA processing essential for MAS and tumour progression.

## Graphical abstract

• *c‐Myc* drives transcription of tRNA processing genes *EPRS* and *LARS* essential for tumour progression.

• *EPRS* and *LARS* facilitate MAS via translational up‐regulation of GOT1 and MDH1 in NB cells.

• LMNA interacts with and inhibits c‐Myc activity in tRNA processing, MAS and NB progression.

• Therapeutic targeting the LMNA‒c‐Myc interaction by lobeline inhibits tumourigenesis and aggressiveness.

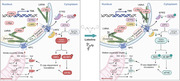

## 1 INTRODUCTION

During translation process, specific amino acids are assembled to 3′‐adenosine of their corresponding transfer RNA (tRNA) by aminoacyl‐tRNA synthetases (ARSs), which are essential for cell homeostasis in physiological context.^1^ According to recent research, dysregulation of ARSs is crucial for carcinogenesis and aggressiveness.^2^ In gastric cancer, elevated glutamyl‐prolyl‐tRNA synthetase (*EPRS*) expression is correlated with poor patients’ outcome.^3^ In lung cancer and osteosarcoma, leucyl‐tRNA synthetase (*LARS*) is up‐regulated and enhances the proliferative or invasive capabilities of tumour cells.^4^ Nevertheless, the functions and regulatory processes of ARSs in the progression of tumours are still largely unknown.

Malate‐aspartate shuttle (MAS), a process transferring cytosolic nicotinamide adenine dinucleotide hydrogen (NADH) into mitochondria, is mainly regulated by glutamic‐oxaloacetic transaminase (GOT) and malate dehydrogenase (MDH), two sets of enzymes localising at cytoplasm or mitochondria.^5^ Within cytoplasm, MDH1 is responsible for the conversion of oxaloacetate and NADH produced by glycolysis into malate and nicotinamide adenine dinucleotide (NAD^+^), while GOT1 converts aspartate and α‐glutaric acid to oxaloacetate and glutamate.^5^ Recent evidence shows that MAS is indispensable for decreasing cytosolic NADH/NAD^+^ ratio that inhibits glycolysis, with slight impact on mitochondrial energy metabolism in cancer cell lines.^5^ Treatment with aminooxyacetic acid, an established MAS inhibitor, decreases the aerobic glycolysis, lactic acid generation, ATP abundance and proliferation of glioma cells via a mitochondria‐independent manner.^6^ However, the regulatory effects of ARSs on MAS in tumours still have not been elucidated.

Herein, c‐Myc is identified to be a powerful transcriptional regulator driving expression of ARSs, which facilitates MAS in neuroblastoma (NB), the prevalent malignant tumour outside the brain in children.^7^ Mechanistically, c‐Myc directly promotes the expression of *EPRS* and *LARS* at transcriptional levels, leading to translational up‐regulation of GOT1 and MDH1 via inhibiting general control nonrepressed 2 (GCN2) or activating mechanistic target of rapamycin (mTOR) signalling. Meanwhile, lamin A (LMNA) physically interacts with and inhibits c‐Myc transactivation. As an identified compound facilitating the interaction of LMNA with c‐Myc, lobeline inhibits *EPRS* and *LARS* expression, MAS, aerobic glycolysis and tumour progression, suggesting the essential interplay of *LMNA* and *c‐Myc* in tRNA processing essential for MAS during tumour progression.

## 2 RESULTS

### 2.1 c‐Myc facilitates tRNA processing gene expression in NB

**FIGURE 1:**
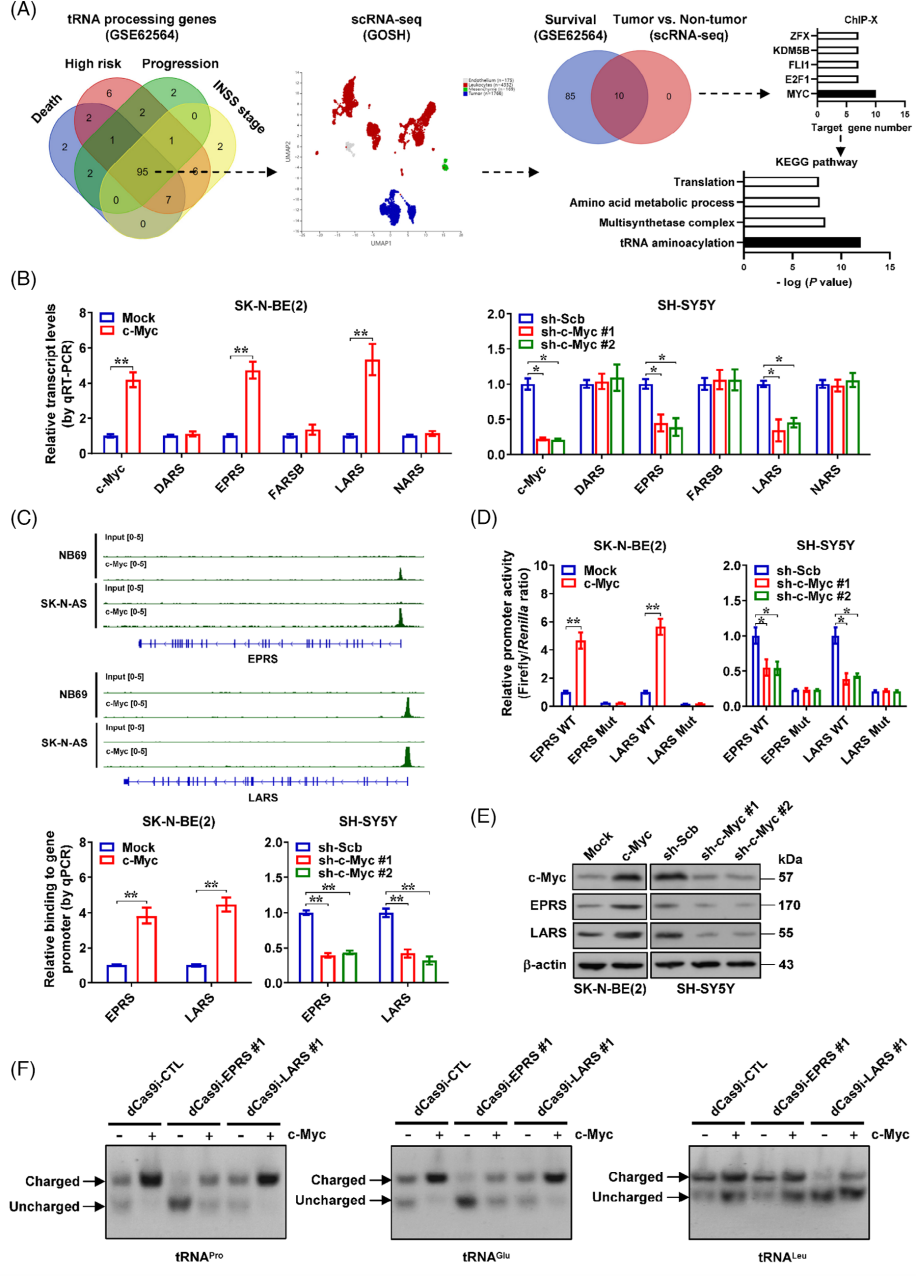
Identification of c‐Myc as a transcription factor regulating transfer RNA (tRNA) processing genes in neuroblastoma (NB). (A) Venn diagram (left and right panels) and U‐map of single‐cell RNA sequencing (scRNA‐seq) results (middle panel) revealing the identification of tumour cell‐abundant tRNA processing genes differentially expressed in 498 NB cases (GSE62564) with various status of death, risk, clinical progression and international neuroblastoma staging system (INSS) stages. ChIP‐X program (right upper panel) and Kyoto Encyclopedia of Genes and Genomes (KEGG) pathway (right lower panel) analyses showing the potential transcription factors regulating tRNA processing genes, and their involvement in biological process. (B) Real‐time quantitative RT‐PCR (qRT‐PCR) assay revealing the transcript levels of *c‐Myc* and tRNA processing genes (normalised to *β‐actin*) in SK‐N‐BE(2) and SH‐SY5Y cells stably transfected with empty vector (mock), *c‐Myc*, scramble shRNA (sh‐Scb) or sh‐c‐Myc (*n *= 5). (C) Chromatin immunoprecipitation sequencing (ChIP‐seq) (GSE138295), ChIP and quantitative PCR (qPCR) assays showing the enrichment of c‐Myc (normalised to input DNA) on promoter region of *EPRS* and *LARS* in NB cells or those stably transfected with mock, *c‐Myc*, sh‐Scb or sh‐c‐Myc (*n *= 5). (D) Dual‐luciferase assay using reporters with wild‐type (WT) or mutant (Mut) c‐Myc binding site indicating the promoter activity of *EPRS* and *LARS* in SK‐N‐BE(2) and SH‐SY5Y cells stably transfected with mock, *c‐Myc*, sh‐Scb or sh‐c‐Myc (*n *= 5). (E) Western blot assay revealing the protein levels of c‐Myc, EPRS and LARS in SK‐N‐BE(2) and SH‐SY5Y cells stably transfected with mock, *c‐Myc*, sh‐Scb or sh‐c‐Myc (*n *= 5). (F) Northern blot assay showing the aminoacylation of tRNA^Pro^, tRNA^Glu^ and tRNA^Leu^ in SK‐N‐BE(2) cells stably transfected with mock or *c‐Myc*, and those co‐transfected with dCas9i control (dCas9i‐CTL), dCas9i‐EPRS #1 or dCas9i‐LARS #1. Fisher's exact test for overlapping analysis in (A). Student's *t*‐test and analysis of variance (ANOVA) compared the difference in (B‒D). ^*^ *p <* .05, ^**^ *p <* .01. Data are shown as mean ± standard error of the mean (s.e.m.) (error bars) or representative of three independent experiments in (B‒F).

To investigate mechanisms regulating tRNA processing gene expression, we performed comprehensive mining of publicly available RNA sequencing (RNA‐seq) results of 498 NB cases (GSE62564),^8^ and identified 95 tRNA processing genes invariably linked to death, high risk, clinical advancement and late stages of international neuroblastoma staging system (INSS, Figure 1A and Table S1). By analysing a dataset of single‐cell RNA‐seq of 6442 cells derived from NB tissues,^9^ 10 tRNA processing enzymes were found to be enriched within tumoural cells, and were strongly correlated with patients’ survival (Figure 1A). Additional integrative analysis via ChIP‐X software^10^ demonstrated that c‐Myc was the primary transcription factor for these tRNA processing genes, with downstream targets engaged in tRNA aminoacylation (Figure 1A). Using SK‐N‐BE(2) as well as SH‐SY5Y as cellular models (representing low or intermediate c‐Myc levels, Figure S1A,B), persistent up‐regulation or down‐regulation of c‐Myc elevated or reduced the amounts of *EPR*S and *LARS* transcripts (Figure 1B). Utilising a publicly available chromatin immunoprecipitation sequencing (ChIP‐seq) dataset of NB cells (GSE138295), endogenous c‐Myc peaks were discovered near the promoter regions of *EPRS* and *LARS* (Figure 1C). Ectopic over‐expression or knockdown of *c‐Myc* facilitated or reduced its enrichment and promoter activity of *EPRS* and *LARS* (Figures 1C,D and S1C‒E), resulting in increase or decrease in their protein expression in NB cells (Figures 1E and S1F). As a control, *MYCN* did not alter the expression of *EPRS* or *LARS* in NB cells (Figure S1G). Ectopic expression of *c‐Myc* led to increase in aminoacyl‐tRNA (tRNA^Pro^, tRNA^Glu^, tRNA^Leu^) production, while knockdown of *EPRS* or *LARS* rescued these alterations (Figure 1F). These findings suggested that at transcriptional levels, c‐Myc promoted the expression of key tRNA processing genes in NB.

### 2.2 *EPRS* and *LARS* facilitate MAS protein expression

**FIGURE 2:**
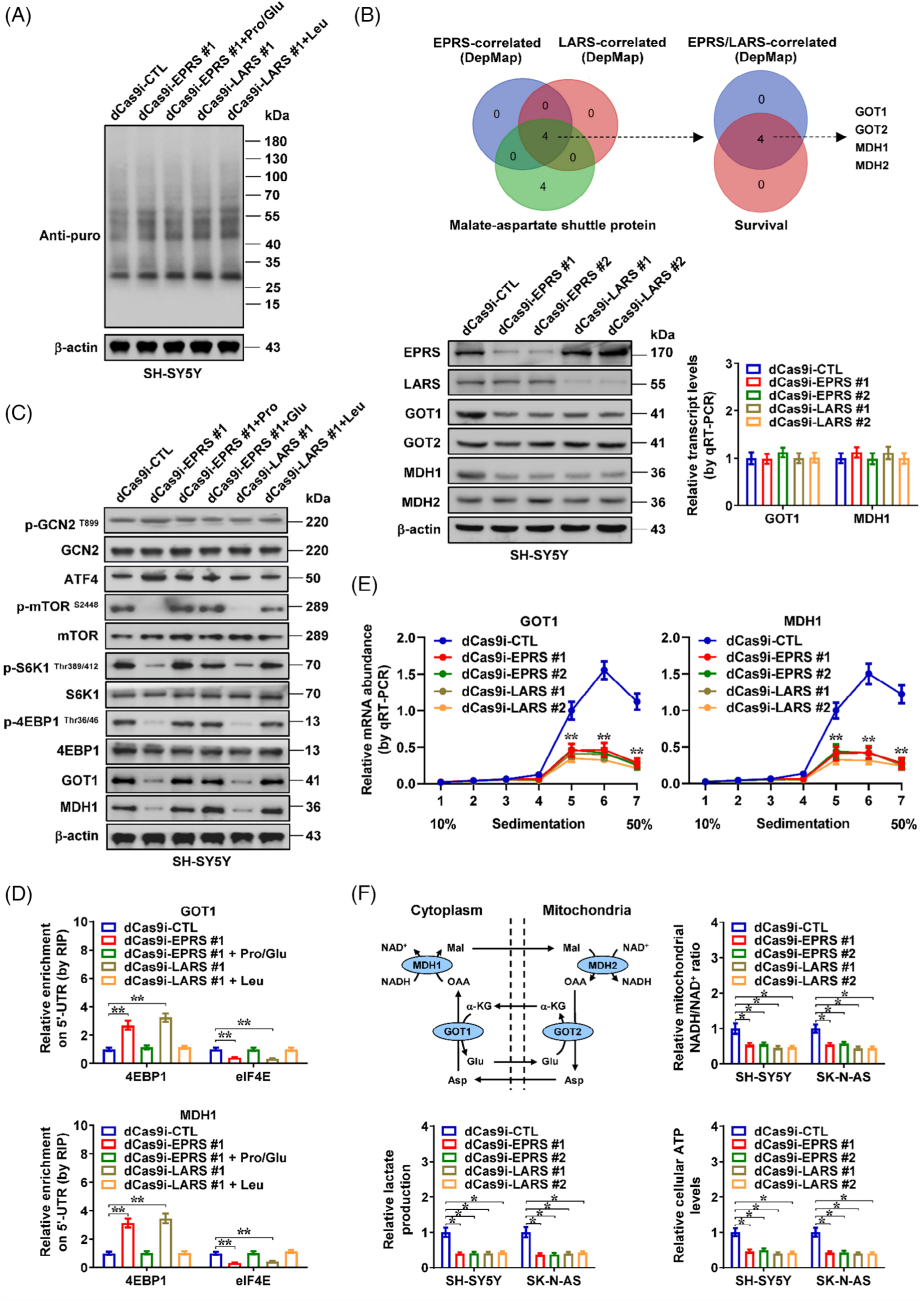
*EPRS* and *LARS* facilitate malate‐aspartate shuttle (MAS) protein expression, tumourigenesis and aggressiveness of neuroblastoma (NB) cells. (A) Puromycin incorporation and western blot assays indicating nascent protein synthesis levels in SH‐SY5Y cells stably transfected with dCas9i control (dCas9i‐CTL), dCas9i‐EPRS or dCas9i‐LARS, and those treated with L‐proline (Pro, 3 mM), glutamate (Glu, 1 mM) or leucine (Leu, 10 mM). (B) Venn diagram (upper panel) showing identification of EPRS‐ and LARS‐correlated MAS proteins derived from DepMap database (https://depmap.org/portal), and those associated with survival of 498 NB cases (GSE62564). Western blot and real‐time quantitative RT‐PCR (qRT‐PCR) assays (normalised to *β‐actin*, lower panels) indicating levels of EPRS, LARS, glutamic‐oxaloacetic transaminase 1 (GOT1), GOT2, malate dehydrogenase 1 (MDH1) and MDH2 in SH‐SY5Y cells stably transfected with dCas9i‐CTL, dCas9i‐EPRS or dCas9i‐LARS. (C) Western blot assay indicating the protein levels of p‐GCN2, general control nonrepressed 2 (GCN2), ATF4, p‐mTOR, mechanistic target of rapamycin (mTOR), p‐S6K1, S6K1, p‐4EBP1, 4EBP1, GOT1 or MDH1 in SH‐SY5Y cells stably transfected with dCas9i‐CTL, dCas9i‐EPRS or dCas9i‐LARS and those treated with L‐proline (Pro, 3 mM), glutamate (Glu, 1 mM) or leucine (Leu, 10 mM). (D) RNA immunoprecipitation (RIP) and qRT‐PCR assays showing the enrichment of 4EBP1 or eIF4E on 5′‐untranslated region (5′‐UTR) of *GOT1* or *MDH1* in SH‐SY5Y cells stably transfected with dCas9i‐CTL, dCas9i‐EPRS or dCas9i‐LARS, and those treated with L‐proline (Pro, 3 mM), glutamate (Glu, 1 mM) or leucine (Leu, 10 mM, *n *= 4). (E) Ribosome enrichment on *GOT1* or *MDH1* messenger RNA (mRNA) in SH‐SY5Y cells stably transfected with dCas9i‐CTL, dCas9i‐EPRS or dCas9i‐LARS. (F) Schematic illustration of MAS (upper left panel) and mitochondrial nicotinamide adenine dinucleotide hydrogen/nicotinamide adenine dinucleotide (NADH/NAD^+^) ratio (upper right panel), lactic acid generation (lower left panel) and ATP levels (lower right panel) of SH‐SY5Y and SK‐N‐AS cells stably transfected with dCas9i‐CTL, dCas9i‐EPRS or dCas9i‐LARS (*n *= 5). Analysis of variance (ANOVA) compared the difference in (B) and (D‒F). ^*^ *p <* .05, ^**^ *p <* .01 versus dCas9i‐CTL. Data are shown as mean ± standard error of the mean (s.e.m.) (error bars) or representative of three independent experiments in (A‒F).

Notably, global protein synthesis was not altered by *EPRS* or *LARS* silencing (Figure 2A). Overlapping analysis of EPRS‐ and LARS‐correlated proteins derived from DepMap database (https://depmap.org/portal) with MAS regulators revealed four potential downstream targets (Figures 2B and S2A and Table S2), including GOT1, GOT2, MDH1 and MDH2, which were substantially linked to survival of 498 cases suffering from NB (GSE62564). Silencing of *EPRS* or *LARS* via clustered regularly interspaced short palindromic repeats (CRISPR)/dCas9‐based strategies led to down‐regulation of *GOT1* and *MDH1*, but not of *GOT2* or *MDH2*, in cultured SH‐SY5Y cells, without alteration in their transcript levels (Figure 2B). In line with previous studies,^11^ knockdown of *EPRS*, but not of *LARS*, induced the phosphorylation of GCN2 and up‐regulation of activating transcription factor 4 (ATF4) within SH‐SY5Y cell line (Figure 2C). Unexpectedly, silencing of both *EPRS* and *LARS* decreased the phosphorylation of mTOR, ribosomal protein S6 kinase 1 (S6K1) and eukaryotic translation initiation factor 4E (eIF4E)‐binding protein 1 (4EBP1), while knockdown of *GCN2* attenuated these changes in response to stable silencing of *EPRS* (Figures 2C and S2B,C). There was increased 4EBP1 enrichment and reduced eIF4E enrichment on 5′‐untranslated region (5′‐UTR) of *GOT1* and *MDH1* in SH‐SY5Y cells upon stable silencing of *EPRS* or *LARS* (Figure 2D). Notably, there was decreased ribosome enrichment on *GOT1* or *MDH1* messenger RNA (mRNA), as well as reduced incorporation of ^3^H‐labedled proline, glutamate or leucine into GOT1 and MDH1 protein, in NB cell lines stably knocking down *EPRS* or *LARS* (Figures 2E and S2D). Meanwhile, supplementation of proline, glutamate or leucine rescued the alteration in GCN2 or mTOR signalling, 4EBP1 and eIF4E enrichment and down‐regulation of GOT1 and MDH1 induced by silencing of *EPRS* or *LARS* (Figure 2C,D). Consistently, knockdown of *EPRS* or *LARS* reduced the mitochondrial NADH abundance, accompanied by increased cytoplasmic NADH and decreased mitochondrial NADH/NAD^+^ ratio, lactic acid generation and ATP amount in NB cells (Figures S2E and 2F). Above results revealed that *EPRS* and *LARS* promoted MAS protein levels in NB cells.

### 2.3 *c‐Myc* promotes MAS via up‐regulating *EPRS* or *LARS* in NB

**FIGURE 3:**
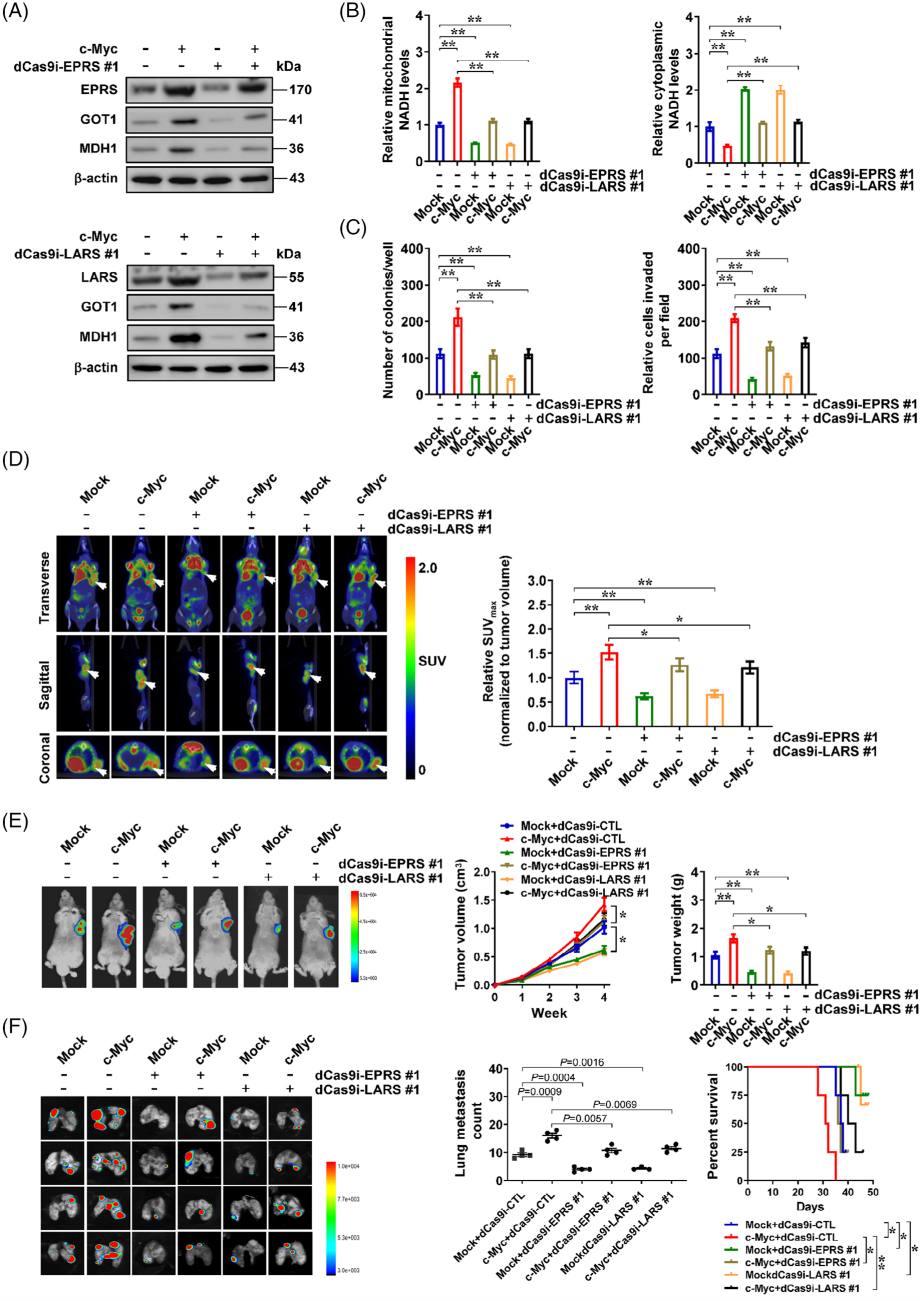
*c‐Myc* promotes malate‐aspartate shuttle (MAS) via up‐regulating *EPRS* or *LARS* in neuroblastoma (NB). (A) Western blot assay indicating expression of EPRS and LARS in SK‐N‐BE(2) cells stably transfected with empty vector (mock) or *c‐Myc*, and those co‐transfected with dCas9i control (dCas9i‐CTL), dCas9i‐EPRS #1 or dCas9i‐LARS #1. (B) Relative mitochondrial and cytoplasmic nicotinamide adenine dinucleotide hydrogen (NADH) levels in SK‐N‐BE(2) cells stably transfected with mock or *c‐Myc*, and those co‐transfected with dCas9i‐CTL, dCas9i‐EPRS #1 or dCas9i‐LARS #1. (C) Soft agar and Matrigel invasion assays showing the anchorage‐independent growth and invasive capabilities of SK‐N‐BE(2) cells stably transfected with mock or *c‐Myc*, and those co‐transfected with dCas9i‐CTL, dCas9i‐EPRS #1 or dCas9i‐LARS #1. (D) 18‐Fluoro‐deoxy‐glucose (^18^F‐FDG) positron emission tomography‒computed tomography (PET‒CT) imaging (left panel) and standardised uptake value (SUV) quantification (right panel) showing the uptake of ^18^F‐labelled glucose (arrowheads) in nude mice with hypodermic xenografts formed by SK‐N‐BE(2) cells stably transfected with mock or *c‐Myc*, and those co‐transfected with dCas9i‐CTL, dCas9i‐EPRS #1 or dCas9i‐LARS #1 (*n *= 3 for each group). (E) In vivo images (left panel), growth curve (middle panel) and weight at the end points (right panel) of xenografts formed by hypodermic injection of SK‐N‐BE(2) cells stably transfected with mock or *c‐Myc*, and those co‐transfected with dCas9i‐CTL, dCas9i‐EPRS #1 or dCas9i‐LARS #1 (*n *= 5 for each group). (F) In vivo imaging (left panel), quantification of lung metastatic colonies (middle panel) and Kaplan‒Meier curves (right panel) of nude mice treated with tail vein injection of SK‐N‐BE(2) cells stably transfected with mock or *c‐Myc*, and those co‐transfected with dCas9i‐CTL, dCas9i‐EPRS #1 or dCas9i‐LARS #1 (*n *= 4 for each group). Analysis of variance (ANOVA) compared the difference in (B‒F). Log‐rank test for survival comparison in (F). ^*^ *p *< .05, ^**^ *p *< .01. Data are shown as mean ± standard error of the mean (s.e.m.) (error bars) or representative of three independent experiments in (A‒C).

We further investigated the roles of *c‐Myc*‐mediated *EPRS* or *LARS* expression in MAS. Ectopic over‐expression of *c‐Myc*‐facilitated GOT1 and MDH1 expression, MAS, mitochondrial NADH/NAD^+^ ratio, lactic acid generation, as well as ATP abundance in SK‐N‐BE(2) cells, while knockdown of *EPRS* or *LARS* rescued these alterations (Figures 3A,B and S3A). Stable *c‐Myc* over‐expression led to increase in growth as well as invasive features of SK‐N‐BE(2) cell line, and rescued the decrease in these pro‐tumoural features induced by *EPRS* or *LARS* silencing (Figures 3C and S3B,C). Elevated ^18^F‐labelled glucose accumulation was noted within xenografts generated by *c‐Myc* over‐expressing NB cell line in nude mice, which was partially rescued by knockdown of *EPRS* or *LARS* (Figure 3D). In vivo imaging assay revealed significantly higher fluorescence intensity in hypodermic xenograft tumours generated by *c‐Myc* over‐expressing NB cell line (Figure 3E). There was a notable rise in growing curve, weight, Ki‐67 (proliferation marker) or CD31 (microvessel marker) levels, mitochondrial NADH/NAD^+^ ratio, lactic acid generation, as well as ATP abundance of hypodermic xenografts formed via NB cells stably over‐expressing *c‐Myc* in nude mice, while knocking down *EPRS* or *LARS* attenuated these alterations (Figures 3E and S4A,B). Furthermore, after NB cells stably over‐expressing c‐Myc were injected via tail vein, nude mice presented an increase in fluorescent signals and lung metastases, and a decrease in survival time, while knockdown of *EPRS* or *LARS* partially abolished these alterations (Figures 3F and S4C). These data indicated that *c‐Myc* promoted MAS via up‐regulating *EPRS* or *LARS* in NB.

### 2.4 LMNA directly interacts with c‐Myc in NB

**FIGURE 4:**
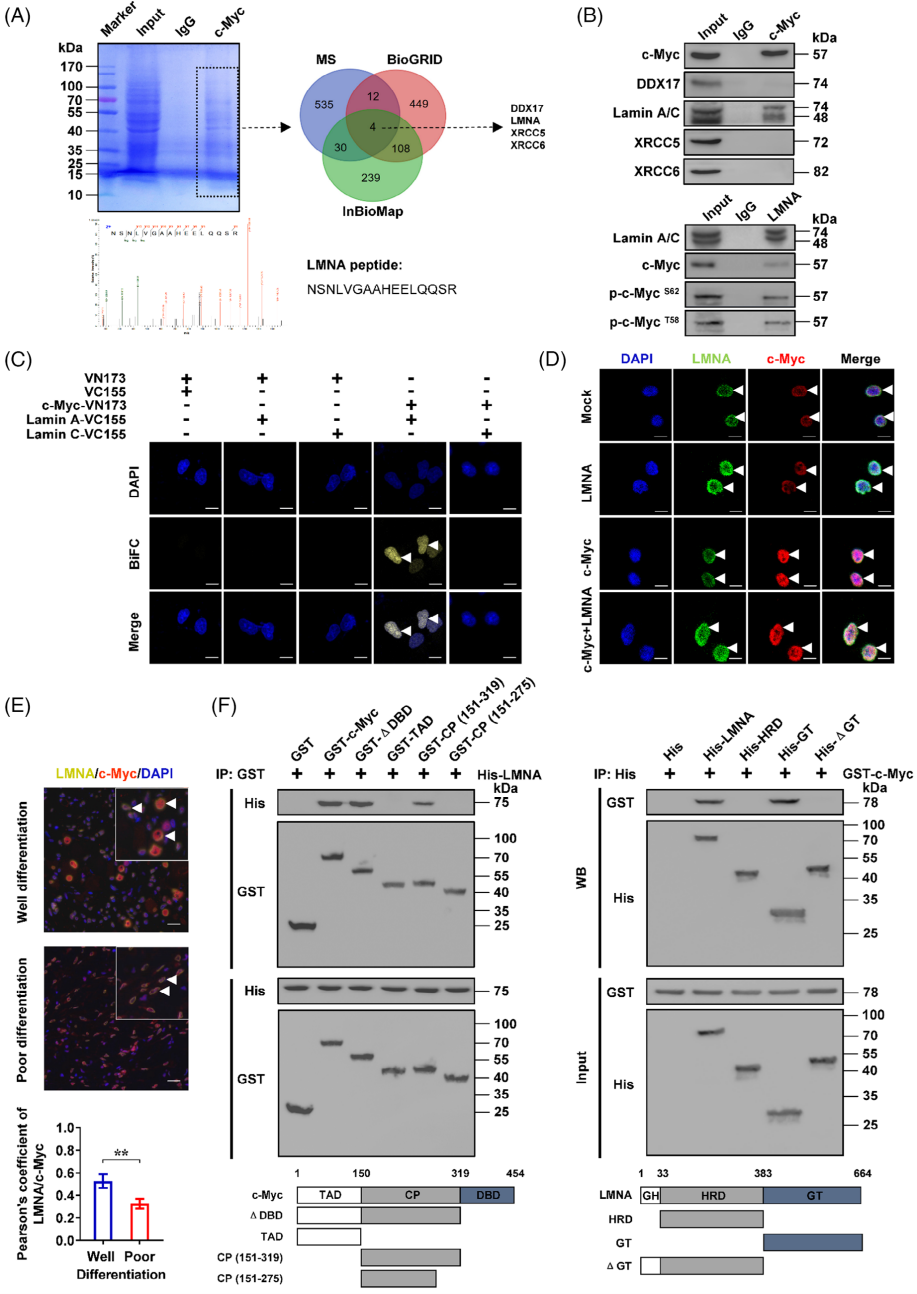
Lamin A (LMNA) directly interacts with c‐Myc in neuroblastoma (NB) cells. (A) Coomassie brilliant blue staining, co‐immunoprecipitation (co‐IP) and mass spectrometry (MS) assays showing differential proteins immunoprecipitated by c‐Myc antibody in SH‐SY5Y cells, with overlapping analysis with c‐Myc‐binding protein derived from BioGRID (https://thebiogrid.org) and InBioMap (https://www.intomics.com/inbio/map.html) databases. (B) Co‐IP and western blot assays indicating endogenous interaction of c‐Myc, p‐c‐Myc^S62^ or p‐c‐Myc^T58^ with DEAD‐box helicase 17 (DDX17), LMNA/C, X‐ray repair cross complementing 5 (XRCC5) or X‐ray repair cross complementing 6 (XRCC6) protein in SH‐SY5Y cells. Immunoglobulin G (IgG)‐bound protein served as a negative control. (C) Representative images of bimolecular fluorescent complimentary (BiFC) assay indicating physical interaction (arrowheads) of c‐Myc with LMNA or LMNC, in SK‐N‐BE(2) cells, with nuclei staining with 4′,6‐diamidino‐2‐phenylindole (DAPI). Scale bars: 10 μm. (D) Representative images of immunofluorescence assay showing co‐localisation of c‐Myc and LMNA (arrowheads) in SH‐SY5Y cells, and those co‐transfected with empty vector (mock), *c‐Myc* or *LMNA*, with nuclei staining with DAPI. Scale bars: 10 μm. (E) Representative images (upper panel) and quantification (lower panel) of immunofluorescence assay revealing the expression of c‐Myc and LMNA (arrowheads) in NB specimens with different differentiation status (*n *= 5), with nuclei staining with DAPI. Scale bars: 50 μm. (F) Co‐IP and western blot assays indicating the direct interaction between recombinant GST‐tagged c‐Myc and His‐tagged LMNA truncation proteins as indicated. ^**^ *p *< .01. Data are representative of three independent experiments in (B‒D) and (F).

To reveal c‐Myc's protein partner, proteomic analysis revealed 581 proteins within SH‐SY5Y cell lysates after immunoprecipitation using c‐Myc antibody (Table S3), which were subjected to overlapping analysis with c‐Myc‐interacting protein in BioGRID^12^ and InBioMap (https://www.intomics.com/inbio/map.html) databases (Figure 4A). The results revealed four potential c‐Myc‐interacting proteins, including DEAD‐box helicase 17 (DDX17), LMNA, X‐ray repair cross complementing 5 (XRCC5) and X‐ray repair cross complementing 6 (XRCC6; Figure 4A). Further validating studies indicated that only LMNA protein was able to interact with c‐Myc and its phosphorylated form at threonine 58 (pT58) or serine 62 (pS62; Figure 4B). Especially, c‐Myc physically interacted with LMNA, but not with lamin C (LMNC) (Figure 4C). Co‐localisation of LMNA and c‐Myc was observed at nuclear periphery or intranuclear foci of NB cell line that was increased following ectopic expression of *LMNA* or *c‐Myc* (Figure 4D). In clinical NB tissues, co‐localisation of LMNA and c‐Myc was also noted at nuclear periphery and intranuclear foci of tumour cells, which was more frequent in specimens with well differentiation (Figure 4E). By using recombinant proteins of c‐Myc and LMNA fused to glutathione S‐transferase (GST)‐ or His‐tag, 276‒319 amino acids (aa) of c‐Myc was found to be necessary for interacting with LMNA, while C‐terminus (383‒664 aa) of LMNA was essential for its direct binding to c‐Myc (Figure 4F). Similar results were observed by transfection of haemagglutinin (HA)‐tagged *c‐Myc* and Flag‐tagged *LMNA* constructs into SH‐SY5Y cell line (Figure S5A). In line with analysis via ZDOCK program,^13^ mutation of 319th threonine residue of c‐Myc or 559th aspartate residue within LMNA abolished their interaction (Figure S5B,C). All these findings suggested the direct binding of LMNA to c‐Myc in NB cells.

### 2.5 LMNA inhibits c‐Myc transactivation essential for *EPRS* or *LARS* expression and MAS in NB

**FIGURE 5:**
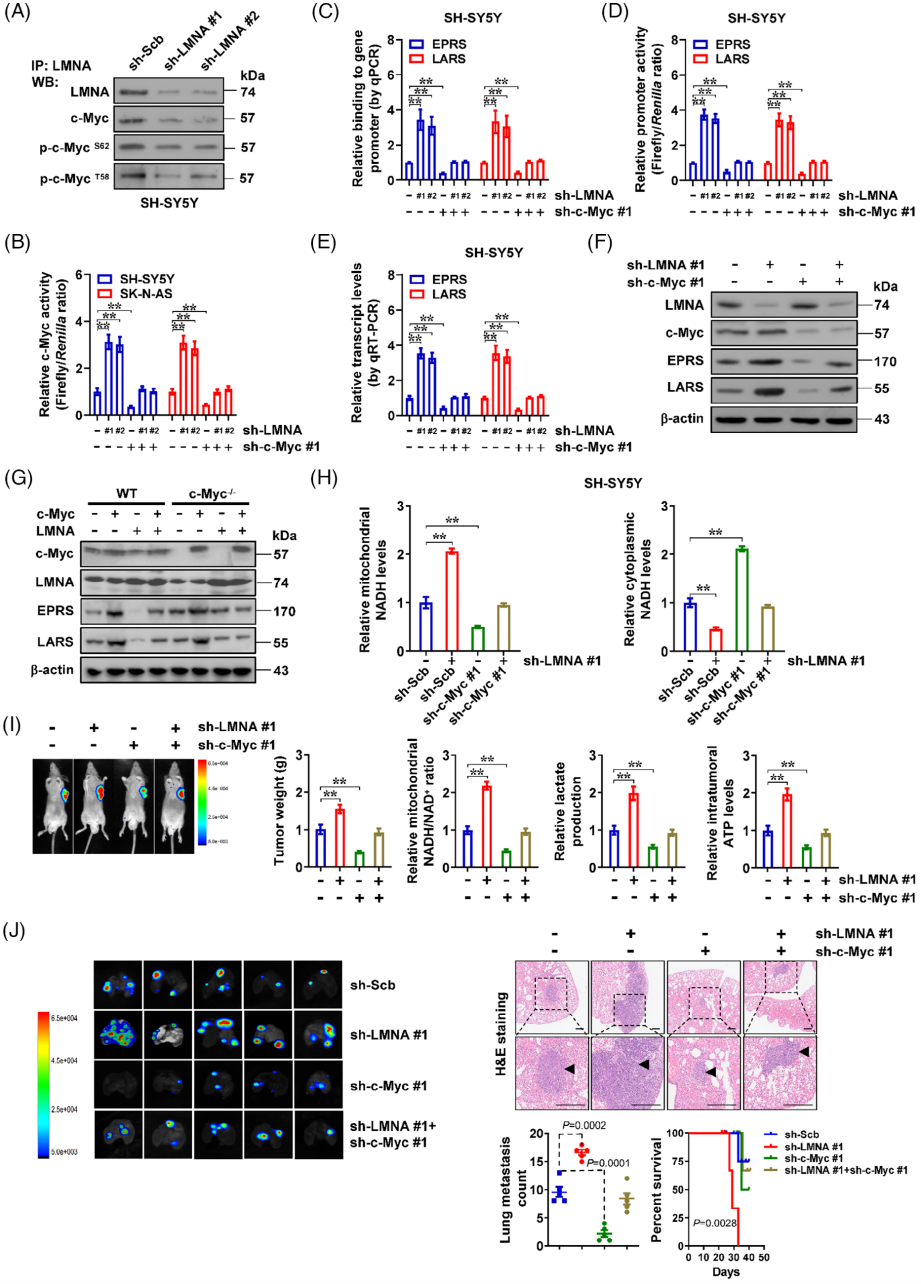
Lamin A (LMNA) inhibits c‐Myc transactivation essential for *EPRS* or *LARS* expression and malate‐aspartate shuttle (MAS) in neuroblastoma (NB). (A) Co‐immunoprecipitation (co‐IP) and western blot assays showing the interaction between LMNA and c‐Myc, p‐c‐Myc^S62^ or p‐c‐Myc^T58^ in SK‐N‐BE(2) and SH‐SY5Y cells stably transfected with scramble shRNA (sh‐Scb), sh‐LMNA #1 or sh‐LMNA #2. (B) Dual‐luciferase assay indicating the activity of a reporter containing three c‐Myc canonical binding sites in SH‐SY5Y and SK‐N‐AS cells stably transfected with sh‐Scb, sh‐c‐Myc #1, sh‐LMNA #1 or sh‐LMNA #2 (*n *= 4). (C and D) Chromatin immunoprecipitation (ChIP) and quantitative PCR (qPCR) (C, normalised to input DNA) and dual‐luciferase (D) assays showing the c‐Myc enrichment and promoter activity of *EPRS* and *LARS* in SH‐SY5Y cells stably transfected with sh‐Scb, sh‐c‐Myc #1, sh‐LMNA #1 or sh‐LMNA #2 (*n *= 5). (E and F) Real‐time quantitative RT‐PCR (qRT‐PCR) (E, normalised to *β‐actin*) and western blot (F) assays indicating the transcript and protein levels of *EPRS* and *LARS* in SH‐SY5Y cells stably transfected with sh‐Scb, sh‐c‐Myc #1, sh‐LMNA #1 or sh‐LMNA #2 (*n *= 5). (G) Western blot assay showing the expression of c‐Myc, LMNA, EPRS and LARS in HEK293T cells with wild‐type or *c‐Myc* knockout (*c‐Myc* ^−/−^), and those transfected with *c‐Myc* or *LMNA* construct. (H) Relative mitochondrial and cytoplasmic nicotinamide adenine dinucleotide hydrogen (NADH) levels in SH‐SY5Y cells stably transfected with sh‐Scb, sh‐c‐Myc #1 or sh‐LMNA #1 (*n *= 4). (I) Representative images (left panel), weight at the end points (left panel), mitochondrial NADH/nicotinamide adenine dinucleotide (NAD^+^) ratio, lactic acid generation and ATP levels (right panel) of xenografts in nude mice formed by hypodermic injection of SH‐SY5Y cells stably transfected with sh‐Scb, sh‐c‐Myc #1 or sh‐LMNA #1 (*n *= 5 for each group). (J) Representative images (left panel), haematoxylin and eosin (HE) staining and quantification of lung metastatic colonisation (arrowheads), and survival curve (right panel) of nude mice treated with tail vein injection of SH‐SY5Y cells stably transfected with sh‐Scb, sh‐c‐Myc #1 or sh‐LMNA #1 (*n *= 5 for each group). Scale bars: 100 μm. Analysis of variance (ANOVA) compared the difference in (B‒E) and (H‒J). Log‐rank test for survival comparison in (J). ^**^ *p *< .01. Data are shown as mean ± standard error of the mean (s.e.m.) (error bars) and representative of three independent experiments in (A‒H).

We further explored the significance of interaction between LMNA and c‐Myc. Excessive expression or knockdown of *LMNA* enhanced or decreased its interaction with p‐c‐Myc^T58^, p‐c‐Myc^S62^ or c‐Myc without alteration in total or phosphated c‐Myc levels (Figures 5A and S5D). Of note, knockdown of *LMNA* with two *LMNA*‐specific short hairpin RNAs (shRNAs; sh‐LMNA #1 and sh‐LMNA #2) increased the transactivation of c‐Myc in SH‐SY5Y and SK‐N‐SH that was prevented by *c‐Myc* silencing (Figure 5B). There was increase in c‐Myc enrichment on *EPRS* and *LARS* promoter regions in *LMNA* stably knocking down NB cells, which was rescued via *c‐Myc* silencing (Figure 5C). In addition, the promoter activation and transcript or protein levels of *EPRS* and *LARS* were increased by stable knockdown of *LMNA*, while silencing of *c‐Myc* rescued these alterations (Figure 5D‒F). To further validate these findings, exogenous *c‐Myc* was transfected into *c‐Myc* knockout (*c‐Myc* ^−/−^) HEK293T cells, leading to up‐regulation of *EPRS* and *LARS*, which was abolished by transfection of *LMNA* (Figure 5G). In addition, the MAS, mitochondrial NADH/NAD^+^ ratio, lactic acid generation, ATP abundance, growth, as well as invasiveness were elevated in SH‐SY5Y cells upon stable *LMNA* silencing, which were partially mitigated after *c‐Myc* knockdown (Figures 5H and S6A,B). Moreover, a significant increase in weight, mitochondrial NADH/NAD^+^ ratio, lactic acid generation, as well as ATP abundance was noted in hypodermically xenografted tumours in athymic mice generated by SH‐SY5Y cells steadily silencing of *LMNA*, which were prevented by silencing of *c‐Myc* (Figure 5I). Athymic nude mice given injections of SH‐SY5Y cells stably silencing of *LMNA* showed more lung metastatic colonies and less survival likelihood, and these effects were partially mitigated by *c‐Myc* knockdown (Figure 5J). Above data suggested that *LMNA* inhibited c‐Myc transactivation essential for *EPRS* or *LARS* expression and MAS in NB.

### 2.6 Lobeline enhances interaction of LMNA with c‐Myc to repress NB progression

**FIGURE 6:**
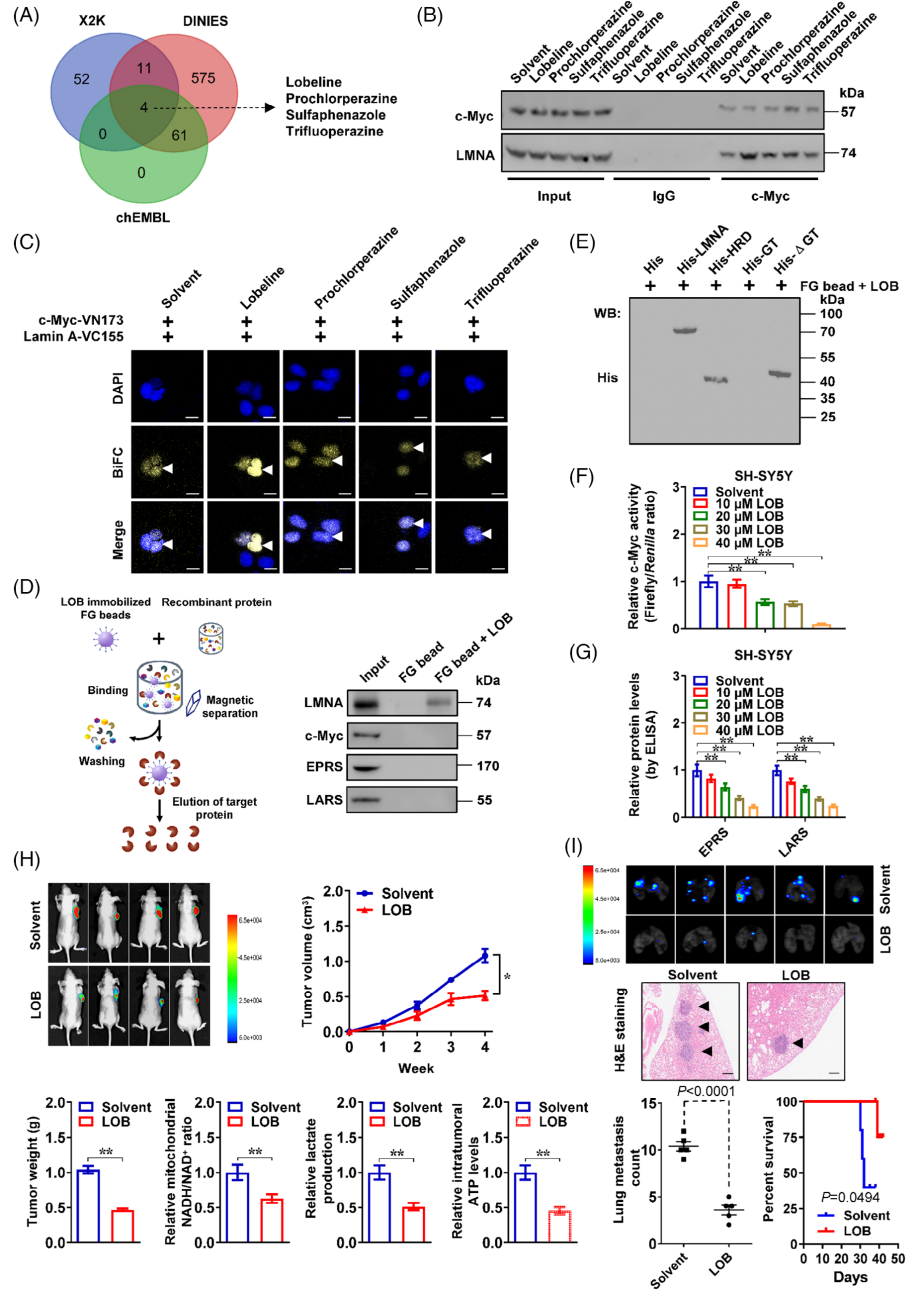
Lobeline (LOB) enhances interaction between lamin A (LMNA) and c‐Myc to repress neuroblastoma (NB) progression. (A) Venn diagram indicating identification of 651 potential LMNA‐interacting compounds derived from DINIES database (https://www.genome.jp/tools/dinies), and overlapping analysis with chemicals affecting expression of target genes (*EPRS* and *LARS*) from X2K database (https://maayanlab.cloud/ X2K), and those influencing LMNA activity (according to Lipinski's Rule of Five) derived from chEMBL database (https://www.ebi.ac.uk/chembl). (B) Co‐immunoprecipitation (co‐IP) and western blot assays showing the interaction of LMNA with c‐Myc in SH‐SY5Y cells treated with four potential compounds (20 μmol/L) for 24 h. (C) Bimolecular fluorescent complimentary (BiFC) assay revealing physical interaction between c‐Myc and LMNA (arrowheads) in SK‐N‐BE(2) cells co‐transfected with pBiFC‐c‐Myc‐VN173 and pBiFC‐Lamin A‐VC155, and those treated with chemicals (20 μmol/L) for 24 h. Scale bars: 10 μm. (D) Schematic illustration (left panel) and western blot (right panel) assays indicating recombinant proteins with affinity to ferriteglycidyl methacrylate (FG) beads covalently conjugated with LOB (10 mmol/L). (E) Western blot assay showing the affinity of recombinant His‐tagged LMNA truncations to FG beads covalently conjugated with LOB (10 mmol/L). (F) Dual‐luciferase assay revealing the c‐Myc transactivation in SH‐SY5Y cells treated with different dosage of LOB as indicated (*n *= 5). (G) Enzyme‐linked immunosorbent assay (ELISA) assay reflecting the levels of EPRS and LARS in SH‐SY5Y cells treated with different dosage of LOB as indicated (*n *= 5). (H) Representative images (upper panel), growth curve (upper panel), weight at the end points (lower panel), mitochondrial nicotinamide adenine dinucleotide hydrogen/nicotinamide adenine dinucleotide (NADH/NAD^+^) ratio, lactic acid generation and ATP levels (lower panel) of hypodermic xenografts formed by SH‐SY5Y cells in nude mice that that received intraperitoneal administration of LOB (5 mg/kg, *n *= 4 for each group). Scale bars: 100 μm. (I) Representative images (upper panel), haematoxylin and eosin (HE) staining (middle panel, arrowheads) and quantification (lower panel) of lung metastatic colonisation, and survival curve (lower panel) of nude mice treated with tail vein injection of SH‐SY5Y cells and dimethyl sulphoxide (DMSO) or LOB (5 mg/kg, *n *= 5 for each group). Analysis of variance (ANOVA) compared the difference in (F‒I). Log‐rank test for survival comparison in (I). ^*^ *p *< .05, ^**^ *p *< .01. Data are shown as mean ± standard error of the mean (s.e.m.) (error bars) and representative of three independent experiments in (B‒G).

By screening public DINIES database,^14^ we discovered 651 compounds potentially binding with LMNA. There were 67 and 65 chemicals affecting target gene (*EPRS* and *LARS*) expression or LMNA activity in X2K (https://maayanlab.cloud/X2K) and chEMBL^15^ databases, respectively. Overlapping analysis revealed four compounds with potential impact on interaction of LMNA with c‐Myc (Figure 6A). Lobeline, but either prochlorperazine, sulphaphenazole or trifluoperazine, was able to facilitate interaction of LMNA with c‐Myc in NB cells (Figure 6B,C). Meanwhile, lobeline did not affect the binding of LMNA to MYCN in SK‐N‐BE(2) cells (Figure S7A). Affinity purification using ferriteglycidyl methacrylate (FG) beads^16^ revealed the direct binding of lobeline to recombinant LMNA protein, but not to that of c‐Myc, EPRS or LARS (Figure 6D), while α‐helical rod domain of LMNA was essential for its affinity with lobeline (Figure 6E). Lobeline treatment reduced the c‐Myc transactivation (Figure 6F) and target gene (*EPRS* and *LARS*) or MAS protein (GOT1 and MDH1) expression, without impact on LMNA or c‐Myc levels (Figures 6G and S7B), which suppressed the viability of NB cell lines, rather than of non‐transformed cells (Figure S7C). Following intraperitoneal delivery of lobeline (5 mg/kg) to nude mice, there was significant reduction in the growing curve, weight, Ki‐67 (proliferation marker) or CD31 (microvessel marker) levels, mitochondrial NADH/NAD^+^ ratio, lactic acid amount, as well as ATP abundance of hypodermically xenografted tumours formed by SH‐SY5Y cell line, along with increase in body weight and down‐regulation of *c‐Myc* downstream genes (Figures 6H and S7D‒F). In addition, lobeline strongly decreased the number of lung metastases and lengthened the alive period of athymic mice subjected to injection of SH‐SY5Y cells via their tail veins (Figure 6I).

**FIGURE 7:**
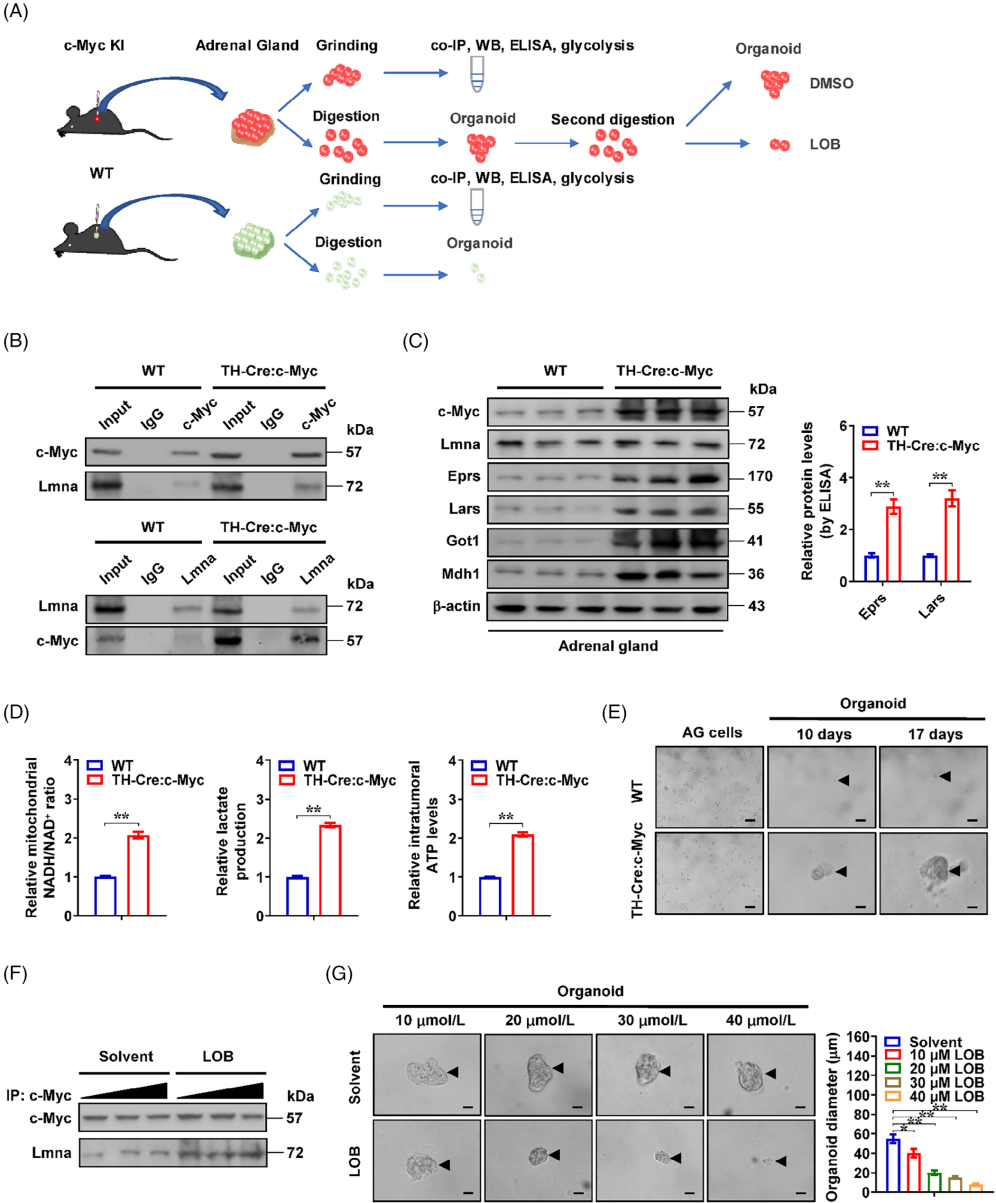
Lobeline (LOB) suppresses organoid growth via facilitating lamin A (LMNA)‒c‐Myc interaction. (A) Schematic illustration of culture and treatment of organoids derived from adrenal glands of wild‐type (WT) or *TH‐Cre*:*c‐Myc* knock‐in (KI) C57BL/6J mice. (B) Co‐immunoprecipitation (co‐IP) and western blot assays showing endogenous interaction between Lmna and c‐Myc within adrenal glands of WT or *TH‐Cre*:*c‐Myc* C57BL/6J mice. (C) Western blot (left panel) and enzyme‐linked immunosorbent assay (ELISA) (right panel, *n *= 5) assays indicating the protein levels of Eprs and Lars in adrenal tissues of WT or *TH‐Cre:c‐Myc* knock‐in mice. (D) Relative mitochondrial nicotinamide adenine dinucleotide hydrogen/nicotinamide adenine dinucleotide (NADH/NAD^+^) ratio, lactic acid generation and ATP levels in adrenal tissues of WT or *TH‐Cre:c‐Myc* knock‐in mice. (E) Representative images of cultured organoids (arrowheads) derived from adrenal tissues of WT or *TH‐Cre:c‐Myc* mice for duration as indicated. Scale bars: 10 μm. (F and G) Co‐IP and western blot assays (F), representative images and quantification (G) indicating the interaction between Lmna and c‐Myc, as well as growth of organoids (arrowheads) derived from adrenal tissues of *TH‐Cre:c‐Myc* mice, and those treated with LOB as indicated. Scale bars: 10 μm. Student's *t* test and analysis of variance (ANOVA) compared the difference in (C), (D) and (G). ^*^ *p *< .05, ^**^ *p *< .01. Data are shown as mean ± standard error of the mean (s.e.m.) (error bars) or representative of three independent experiments in (B‒G).

In established *TH‐Cre:c‐Myc* knock‐in mice (Figure 7A), there was increase in Lmna‒c‐Myc interaction, *Eprs*, *Lars*, *Got1* and *Mdh1* levels, mitochondrial NADH/NAD^+^ ratio, lactic acid generation, ATP abundance and organoid formation of adrenal gland cells, than those in wild‐type mice (Figure 7B‒E). Meanwhile, lobeline significantly increased the interaction between Lmna and c‐Myc, and suppressed the growth of organoids derived from adrenal gland cells of *c‐Myc* knock‐in mice (Figure 7F,G). Taken together, these data indicated that lobeline enhanced interaction of LMNA with c‐Myc to repress NB progression.

### 2.7 Lobeline inhibits tumour progression by facilitating LMNA‒c‐Myc interaction

We further determined the involvement of LMNA and c‐Myc in lobeline‐repressed tumourigenesis and aggressiveness. Over‐expression of *c‐Myc* or knockdown of *LMNA* prevented SH‐EP and SH‐SY5Y cells from reduction in c‐Myc transactivation and promoter activity or expression of target genes (*EPRS* and *LARS*) induced by lobeline (Figure S8A‒C). The lobeline‐induced alteration in mitochondrial or cytoplasmic NADH levels was partially restored by over‐expression of *c‐Myc* or silencing of *LMNA* (Figure S8D), accompanied by rescue in mitochondrial NADH/NAD^+^ ratio, lactic acid generation and ATP abundance (Figure S9A,B). Ectopic expression of *c‐Myc* or knockdown of *LMNA* partially prevented SH‐EP and SH‐SY5Y cells from decrease in growth or invasive capabilities following lobeline treatment (Figure S9C). These results indicated that lobeline inhibited NB progression by facilitating interaction of LMNA and c‐Myc.

### 2.8 *LMNA*, *c‐Myc* and downstream targets are linked to prognosis of patients with tumours

**FIGURE 8:**
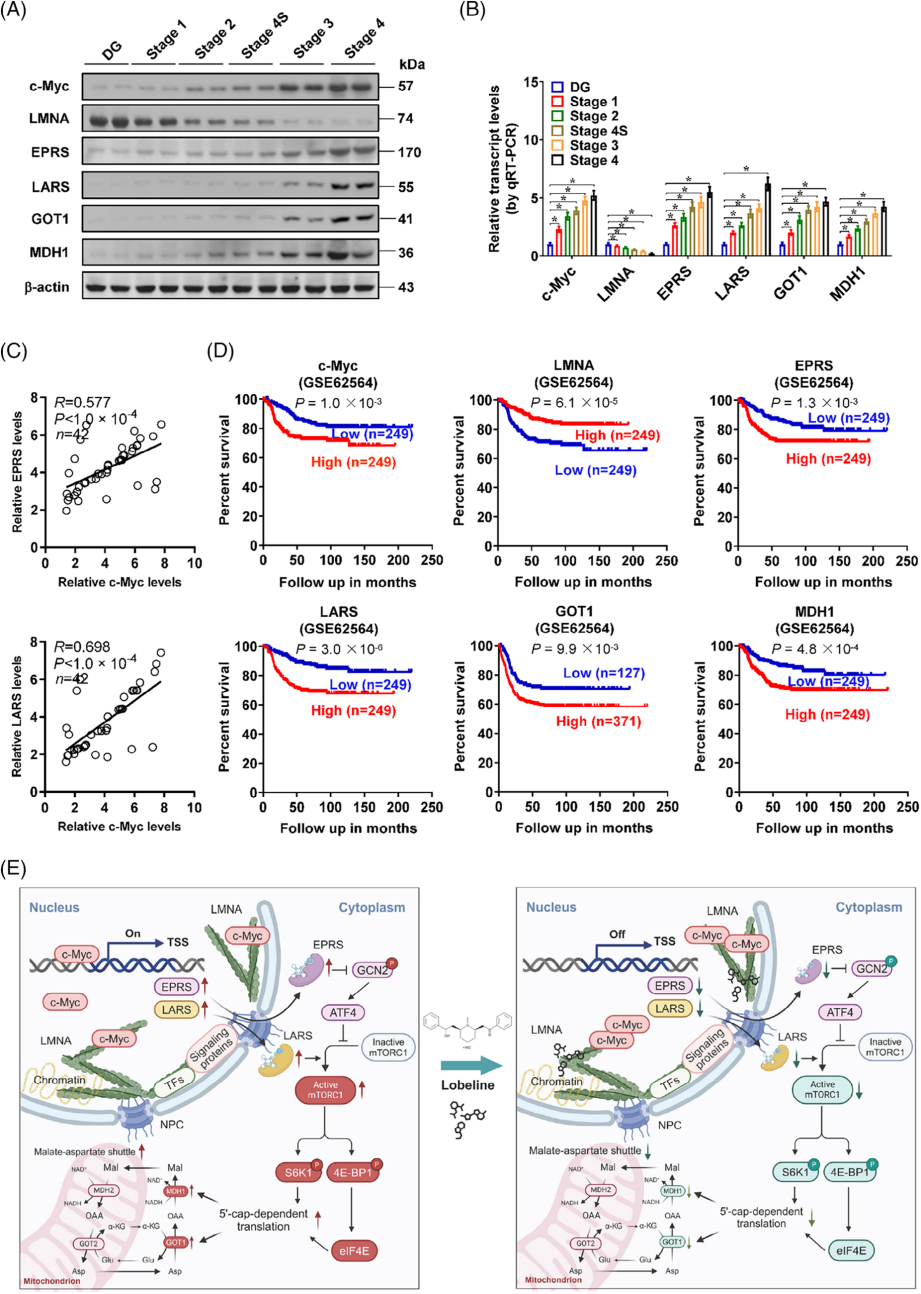
*LMNA*, *c‐Myc* and target genes are associated with outcome of neuroblastoma (NB) patients. (A and B) Western blot (A) and real‐time quantitative RT‐PCR (qRT‐PCR) (B, normalised to *β‐actin*) assays showing the expression of *LMNA*, *c‐Myc*, transfer RNA (tRNA) processing genes (*EPRS* and *LARS*) and malate‐aspartate shuttle (MAS) genes (*GOT1* and *MDH1*) in normal dorsal ganglia (DG) and NB tissues (*n *= 30) with different international neuroblastoma staging system (INSS) stages. (C) The expression correlation of *c‐Myc* with *EPRS* or *LARS* in 42 NB tissues. (D) Kaplan‒Meier curves indicating overall survival of well‐defined 498 NB cases (GSE62564) with high or low expression of *c‐Myc* (cutoff value = 4.6), *LMNA* (cutoff value = 6.2), *EPRS* (cutoff value = 6.9), *LARS* (cutoff value = 6.9), *GOT1* (cutoff value = 4.9) or *MDH1* (cutoff value = 6.1). (E) The mechanisms underlying LMNA‐inhibited c‐Myc activity in tRNA processing, MAS and NB progression: LMNA interacts with c‐Myc to suppress its activity, resulting in transcriptional repression of *EPRS* and *LARS*, translational down‐regulation of glutamic‐oxaloacetic transaminase 1 (GOT1) and malate dehydrogenase 1 (MDH1) via activating general control nonrepressed 2 (GCN2) or inhibiting mechanistic target of rapamycin (mTOR) signalling, reduction of MAS and decrease in tumourigenesis and aggressiveness. As a LMNA‐binding compound, lobeline facilitates the interaction of LMNA with c‐Myc, resulting in inhibition of c‐Myc activity and tumour progression. Meanwhile, the other potential roles of LMNA in NB progression, such as anchoring heterochromatin and binding with transcription factors (TFs) or signalling proteins, warrant further studies. Analysis of variance (ANOVA) compared the difference in (B). Pearson's correlation coefficient analysis for gene expression in (C). Log‐rank test for survival comparison in (D). ^*^ *p *< .05. Data are shown as mean ± standard error of the mean (s.e.m.) (error bars) in (B).

Higher levels of *c‐Myc*, *EPRS*, *LARS*, *GOT1* and *MDH1*, as well as lower expression of *LMNA* were detected in NB specimens derived from late stages of INSS (Figure 8A,B). In 42 cases of NB, positive tendencies were noted among the expression of c‐Myc and its targets (*EPRS* or *LARS*; Figure 8C). In Kaplan‒Meier survival analysis, higher levels of *c‐Myc* (*p *= 1.0 × 10^−3^), *EPRS* (*p *= 1.3 × 10^−3^), *LARS* (*p *= 3.0 × 10^−6^), *GOT1* (*p *= 9.9 × 10^−3^) or *MDH1* (*p *= 4.8 × 10^−4^), and lower levels of *LMNA* (*p *= 6.1 × 10^−5^) were linked to worse outcome of 498 NB cases (GSE62564; Figure 8D). Higher expression of *c‐Myc* or low levels of *LMNA* were also correlated with worse prognosis of clinical cases suffering from B‐cell lymphoma, colon carcinoma, diffuse large B‐cell lymphoma, glioma, melanoma, osteosarcoma or sarcoma (Figure S10). These results indicated that *LMNA*, *c‐Myc* and downstream targets were linked to prognosis of patients with tumours.

## 3 DISCUSSION

c‐Myc modulates expression of around 15% of human genes through binding to E‐box element within their promoters.^17^ As an independent indicator of high risk, *c‐Myc* expression is elevated in 11% of NB cases.^18^ In zebrafish model, dopamine beta‐hydroxylase (*Dbh*) promoter‐driven *c‐Myc* transgene is even more potent than that of *MYCN* in inducing NB formation, indicating the oncogenic role of *c‐Myc* in NB development.^19^ Recent evidence shows that ARSs are potential mediators of c‐Myc‐facilitated growth and survival in *Drosophila*.^20^ In this study, as an oncogene amplified in 25% NB cases, *MYCN* did not affect expression of *EPRS* and *LARS*. Instead, *c‐Myc* drove the expression of *EPRS* and *LARS* at transcriptional levels, leading to translational up‐regulation of MAS proteins (GOT1 and MDH1) via inhibiting GCN2 or activating mTOR signalling. Meanwhile, LMNA inhibited c‐Myc transactivation to repress expression of these ARSs and downstream MAS proteins (Figure 8E). Notably, *c‐Myc* possessed oncogenic properties in tRNA aminoacylation and MAS, extending current knowledge regarding its acting mechanisms in tumour progression.

In human cells, there are two major regulatory mechanisms for amino acid sensing, including GCN2 and mTOR signalling pathways.^21^ In response to decreased amino acid levels or inhibited ARSs, GCN2 kinase binds tightly to uncharged tRNA to stimulate its autophosphorylation, resulting in up‐regulation of ATF4 to induce gene expression essential for amino acid metabolism.^22^ Inhibition of EPRS significantly stimulates GCN2 phosphorylation, which is abrogated by proline.^11^ Through direct sensing amino acid levels, mTOR phosphorylates downstream effectors S6K1 and 4EBP1, leading to eIF4E activation and 5′ cap‐dependent protein translation.^23^ As a leucine sensor, LARS activates mTOR signalling for protein synthesis,^24^ and is up‐regulated in lung cancer.^25^ In this study, our results indicated that *c‐Myc*‐facilitated MAS via regulating *EPRS* and *LARS* expression, revealing a novel axis for therapeutic vulnerability. Notably, knockdown of *EPRS* or *LARS* did not affect global protein synthesis, while their effects on MAS and tumour progression are exerted, at least in part, through GOT1 or MDH1. In line with previous studies,^21^ activated GCN2 repressed the mTOR kinase activity in NB cells, suggesting that specific protein synthesis rather than global translation contributes to the roles of *EPRS* or *LARS* in NB progression.

As nucleoplasmic scaffolds, LMNA/C anchor heterochromatin and are involved in epigenetic regulation of gene transcription.^26^ LMNA binds with retinoblastoma protein to protect its proteasomal degradation,^27^ and cooperates with c‐Fos to suppress the functions of activator protein 1.^28^ Through protein interaction, LMNA is also able to regulate the activity of proteins involved in signalling cascades, such as β‐catenin^29^ and SMAD family member 2.^30^ Importantly, LMNA expression is reduced in multiple malignancies, such as gastric or ovarian cancers, while abnormal fusion of *LMNA*‐*NTRK1* gene may cause tumourigenesis.^31^ In NB cells, *LMNA* is down‐regulated probably due to promoter hypermethylation.^32^ In response to neurotrophic receptor tyrosine kinase 1 activation, LMNA is accumulated within nuclear foci and associated with differentiation of NB cells in vitro.^33^ Our results revealed low expression of *LMNA* within NB tissues that was related to worse prognostic outcome of clinical cases. Importantly, LMNA interacted with and inhibited c‐Myc transactivation essential for ARSs expression. Previous studies show that hierarchical phosphorylation at S62 and T58 is essential for the transactivation and stability of c‐Myc protein,^34^ while S62 phosphorylation enhances the retention of c‐Myc within LMNA/C‐related nuclear foci during regenerative proliferation.^35^ In this study, we found that LMNA bound to total and phosphorylated (S62, T58) forms of c‐Myc at nuclear periphery or intranuclear foci, and repressed the roles of c‐Myc in increasing expression of *EPRS* and *LARS*, providing a therapeutic approach targeting c‐Myc within tumours.

In recent years, it is an urgent duty to identify small molecule compounds or inhibitory approaches targeting c‐Myc activity. In this study, we identified lobeline as a LMNA‐binding inhibitor of c‐Myc activity in NB cells. Lobeline is a natural alkaloid derived from *Lobelia inflate*, and has beneficial actions via affinities for nicotinic acetylcholine receptor, vesicular monoamine transporter, opioid receptor^36^ or N‐methyl‐D‐aspartate receptor.^37^ Thus, it is clinically applied as a therapeutic agent for psychostimulant abuse^38^ or tobacco smoking cessation.^39^ Of note, lobeline is able to reverse multidrug resistance of tumour cells via inhibiting P‐glycoprotein activity.^40^ However, the targets, action mode and biological activities of lobeline in tumour cells still remain elusive. Our findings indicated that lobeline was potent in suppressing carcinogenesis, tumourigenesis and aggressiveness, and improving survival time of nude mice bearing xenografts, which provided an important therapeutic agent for *c‐Myc*‐driven tumours.

## 4 CONCLUSIONS

In conclusion, we demonstrate that c‐Myc and LMNA exert crucial roles in tRNA processing‐facilitated MAS during NB progression. Mechanistically, c‐Myc facilitates MAS by up‐regulating *EPRS* and *LARS*, while LMNA suppresses c‐Myc transactivation via physical interaction, leading to repressed MAS, aerobic glycolysis, growth, invasion and metastasis of NB cell lines. Lobeline, a compound facilitating LMNA‒c‐Myc interaction, suppresses MAS and tumour progression of NB, while the underlying structural mechanisms warrant further studies. Our study is helpful for extending current findings regarding transcriptional activation of ARSs and involvement of tRNA processing in MAS, and suggests the *LMNA*/*c‐Myc* axis as a prospective vulnerability for tumour treatment. Meanwhile, other protein partners and fundamental mechanisms of LMNA during NB progression warrant further investigation. Considering the multiple actions of lobeline, its derivates or analogues with structural modification warrant further studies to facilitate the interaction of LMNA with c‐Myc for tumour treatment, especially by using *c‐Myc* knock‐in mice.

## 5 METHODS

### 5.1 Cell lines

Human NB (SK‐N‐BE(2), SK‐N‐AS, SK‐N‐SH, SH‐EP, SH‐SY5Y) and mammary epithelial MCF 10A (non‐transformed) cells were acquired from the American Type Culture Collection. Human *c‐Myc* ^−/−^ HEK293T cell line was acquired from EdiGene Biotechnology Inc. The cell lines were verified for authenticity by short tandem repeat loci, and were utilised for less than 6 months after being revived from frozen samples. Mycoplasma detection was routinely assessed with the MycoAlert Kit (Promega). The cells were cultivated in Dulbecco's modified Eagle's medium, which was supplemented with 10% foetal bovine serum from Gibco. Additionally, the cells were subjected to treatment with L‐proline, glutamate, leucine or lobeline (Sigma).

### 5.2 Real‐time quantitative PCR

For total RNA isolation, MagneSil Total RNA Mini‐Isolation System (Promega) was applied. The process of reverse transcription was carried out using a GoScript Reverse Transcription System from Promega. PCR amplification was conducted using GoTaq Green Master Mix (Promega), along with primers listed in Table S4.

### 5.3 Western blotting

For protein extraction, cell lysis buffer (ab288311) was used. Western blotting was conducted according to previously methods,^41^, ^42^, ^43^, ^44^, ^45^ with antibodies against c‐Myc (ab32072), p‐c‐Myc^S62^ (ab51156), p‐c‐Myc^T58^ (ab28842), MYCN (ab227822), LMNA (ab108595, ab8980), EPRS (PA5‐56323, Invitrogen), LARS (ab96221), GOT1 (ab221939), GOT2 (A19245, Abclonal), MDH1 (ab180152), MDH2 (ab96193), DDX17 (ab24601), XRCC5 (ab119935), XRCC6 (ab92450), phosphorylated GCN2 (T899, ab75836), GCN2 (ab68427), ATF4 (ab184909), phosphorylated mTOR (S2448, ab109268), mTOR (ab32028), phosphorylated S6K1 (T389/T412, ab60948), S6K1 (ab32359), phosphorylated 4EBP1 (T37/T46, ab278686), 4EBP1 (ab32024), HA‐tag (ab9110), Flag‐tag (ab125243), His‐tag (ab18184), GST (ab19256) or β‐actin (ab8226, Abcam Inc.).

### 5.4 Affinity purification

The FG beads (Nacalai Tesque, Inc.) were incubated with 20 mmol/L lobeline in N,N‐dimethylformamide. Lobeline‐immobilised beads (.5 mg) were incubated with recombinant protein or cell lysates for 2 h at 4°C, and rinsed using .5% NP‐40 lysis buffer, while recovered proteins were analysed by western blotting.^16^

### 5.5 Dual‐luciferase reporter assay

The promoter region of either *EPRS* (−398/+68) or *LARS* (−801/+91) was obtained by PCR with primers (Table S5) and inserted into the pGL3‐Basic vector (Promega). The c‐Myc binding site was mutated using QuikChange Multi Site‐Directed Mutagenesis Kit (Agilent) and primer sets listed in Table S5. A luciferase reporter for c‐Myc transactivation was created by introducing oligonucleotides (Table S5) containing three standard motifs into the pGL4.23 vector (Promega). The dual‐luciferase assay was conducted following the previously established protocol.^41^, ^42^, ^44^, ^45^

### 5.6 ChIP assay

ChIP experiment was conducted using the High‐Sensitivity ChIP kit (ab185913).^41^, ^42^, ^44^, ^45^ Antibodies targeting c‐Myc (ab32072) or LMNA (ab108595, Abcam Inc.) were utilised. The SYBR Green Quantitative RT‐qPCR Kit (Sigma) and primers (Table S4) were used to conduct real‐time quantitative PCR (qPCR).

### 5.7 RNA immunoprecipitation

Tumour cells (1 × 10^8^) were subjective to ultraviolet light (wavelength of 254 nm, energy dose of 200 J/cm^2^).^41^, ^45^ RNA immunoprecipitation was conducted using the Imprint RNA Immunoprecipitation Kit (Sigma). Antibodies for 4EBP1 (ab32024) or eIF4E (ab33766, Abcam Inc.) and primers (Table S4) were used.

### 5.8 Northern blot

Northern blot was undertaken with 5′‐biotinylated oligonucleotide probes (3 ng/μL, Table S5),^46^ incubated by streptavidin‒horseradish peroxidase conjugate, and detected on X‐ray films using CSPD substrate (Sigma).

### 5.9 Constructs

Human *c‐Myc* cDNA (1365 bp) and *MYCN* construct were kind gifts from Drs. William P. Tansey and Arturo Sala. The *LMNA* cDNA (1995 bp) was introduced into lentiviral construct from GeneChem Co., Ltd. Their truncated fragments were prepared by PCR assay with primer sets (Table S5), and introduced into pCMV‐HA, pCMV‐3Tag‐1C, pET28‐A or pGEX‐6P‐1 (Addgene). Oligonucleotides specific for shRNAs or small guide RNAs (Table S6) were introduced into GV298 (GeneChem Co., Ltd.) or dCas9‐BFP‐KRAB (Addgene).

### 5.10 Lentivirus preparation

HEK293T cell line was co‐transfected with lentiviral construct along with packaging vectors (psPAX2 and pMD2G, Addgene). The infectious lentivirus was passed through .45 μm PVDF filters, and collected using ultracentrifugation at 120 000 ×*g* for 2 h.

### 5.11 Immunofluorescence assay

Tumour cells or tissues were fixed with 4% paraformaldehyde, subjected to antibodies (1:100 dilution) against c‐Myc (ab32072) or LMNA (ab108595, Abcam Inc.), and treated by Alexa Fluor 488 (A31627), Alexa Fluor 594 (A11012) or Alexa Fluor 532 (A11009, Thermo Fisher Scientific, Inc.) goat anti‐rabbit immunoglobulin G. Then, samples were stained by 300 nmol/L of 4′,6‐diamidino‐2‐phenylindole.^44^, ^45^ For each section, more than five areas were randomly chosen, while co‐localisation of LMNA and c‐Myc signals was analysed via Person's correlation coefficient by using ImageJ program (https://imagej.nih.gov/ij).

### 5.12 Co‐immunoprecipitation, GST pull‐down and mass spectrometry

Co‐immunoprecipitation and GST pull‐down were carried out according to previously established methods,^44^, ^45^ using specific antibodies for c‐Myc (ab32072), LMNA (ab108595), HA‐tag (ab9110), Flag‐tag (ab125243), His‐tag (ab18184) or GST (ab19256, Abcam Inc.). Immunoprecipitated proteins were then separated by sodium dodecyl sulphate‒polyacrylamide gel electrophoresis, and subjected to Coomassie blue staining, western blotting or mass spectrometry at SpecAlly Life Technology Co., Ltd.^41^, ^42^, ^44^, ^45^

### 5.13 Bimolecular fluorescent complimentary detection

Human cDNAs of *c‐Myc* (1365 bp), *LMNA* (1995 bp) and *LMNC* (1719 bp) were inserted into pBiFC‐VN173 (containing Flag‐tag) or pBiFC‐VC155 (containing HA‐tag) from Addgene. The *c‐Myc* and *LMNA* constructs were mutated by QuikChange Multi Site‐Directed Mutagenesis Kit (Agilent) using primer sets (Table S5). Constructs were co‐transfected into tumour cells by using NeuroPorter Transfection Kit (Sigma) for a 24‐h period, while fluorescence imaging was detected using a confocal microscope.^41^, ^42^, ^44^, ^45^

### 5.14 Enzyme‐linked immunosorbent assay

The EPRS or LARS levels were determined by enzyme‐linked immunosorbent assay (MyBioSource, Inc.). The absorbance was observed at 450 nm of wavelength, using a Varioskan LUX spectrophotometer obtained from Thermo Fisher Scientific, Inc.

### 5.15 Sucrose gradient sedimentation

Polysomal fractions were prepared according to previously mentioned procedure.^44^, ^47^ Using *β‐actin* as a housekeeping gene, the quantities of polysome‐bound transcripts were normalised to free RNAs using real‐time quantitative RT‐PCR.

### 5.16 Amino acid incorporation assay

Cell lysates were resuspended with reaction buffer (5 mmol/L ATP, 1 mmol/L spermine, 25 mmol/L KCl, 5 mmol/L MgCl_2_, 1 μmol/L ^3^H proline, 50 mmol/L HEPES‒KOH [pH 7.6], glutamate or leucine [60 Ci/mmol, Perkin‐Elmer]), and incubated with GOT1 (ab221939) or MDH1 (ab180152, Abcam Inc.) antibody. Aliquots (15 μL) of immunoprecipitated proteins were quenched on Whatman filter paper, while radioactivity was quantified using Beckman Coulter LS6500 (Beckman Conlter, Inc.).

### 5.17 Protein synthesis assay

Cells were incubated with puromycin (10 μg/mL), an aminoacyl‐tRNA analogue incorporating into nascent polypeptides during translation.^48^ Puromycylated peptides were measured via western blotting and puromycin‐specific antibody (ab315887, Abcam Inc.; 1:1000).

### 5.18 MAS detection

Subcellular or tissue fractions were isolated by using Mitochondria/Cytosol Fractionation Kit (ab65320) or Mitochondria Isolation Kit for Tissue (ab110168, Abcam Inc.). The amount of NADH or NAD^+^ was detected using a NAD^+^/NADH Assay Kit (ab221821) from Abcam Inc.^49^ Measurement of lactic acid generation as well as ATP abundance was undertaken as documented.^41^, ^42^

### 5.19 Measurement of tumour cell aggressiveness

Cellular viability, growth, as well as invasive features were evaluated by methylthialazole tetrazolium (Thermo Fisher Scientific, Inc.) colorimetric,^41^ soft agar colony formation^41^, ^42^, ^44^, ^45^, ^50^ and Matrigel invasion^41^, ^42^, ^44^, ^45^, ^50^ methods.

### 5.20 Organoid culture

After being split into pieces with 2 mm in diameter, adrenal glands of 1‐month‐old mice were trypsinised in Trypsin‐ethylene diamine tetraacetic acid, and filtered using 70‐μm cell strainers (Sigma). The single‐cell suspensions were combined with 10 μg/mL of cold 3D Matrigel (Corning) and cultivated in media supplemented with 1% B‐27 (Gibco), 1% N2 (Gibco), 20 ng/mL epidermal growth factor (Peprotech) and 40 ng/mL basic fibroblast growth factor (Sigma).^51^

### 5.21 *Myc* knock‐in mice

To generate *Myc* knock‐in mice, the CAG‐loxp‐PGK‐Neo‐pA‐loxp‐*Myc*‐WPRE‐pA expression frame was inserted at Hipp11 gene site via CRISPR/Cas9 technology. The mRNA of *Cas9* and gRNA were in vitro transcribed, and donor construct was created using the In‐Fusion cloning technique. Experimental mice were obtained by hybridising with *TH‐Cre* tool mice at Shanghai Biomodel Organism Science & Technology Development Co., Ltd.

### 5.22 Nude mice studies

The Huazhong University of Science and Technology's Experimental Animal Ethics approved all mice researches that were undertaken in conformity with NIH Guidelines for the Care and Use of Laboratory Animals. Using male BALB/c nude mice (aged 4 weeks old), hypodermic or tail vein injection of tumour cells was conducted at random.^42^, ^44^, ^45^ For therapeutic investigation, seven days after hypodermic or tail vein injection of 5 × 10^6^ tumour cells, randomly grouped male BALB/c nude mice were treated with dimethyl sulphoxide or lobeline (5 mg/kg).^41^, ^42^, ^44^, ^45^ Fluorescence values of mice were obtained by In‐Vivo Xtreme II (Bruker Corporation) or Lago X Imaging (Spectral Instruments Imaging) system.

### 5.23 Positron emission tomography/computed tomography imaging

After a 12‐h fasting, mice received a 60 min intravenous infusion of 18‐fluoro‐deoxy‐glucose (^18^F‐FDG, 200 ± 10 μCi). The TransPET Discoverist 180 system (RAYCAN Technology Co., Ltd.) was used to acquire the positron emission tomography/computed tomography images. The formula (mean pixel value with decay‐corrected region‐of‐interest activity), calculated as (μCi/kg)/(injected dosage [μCi]/weight [kg]), was applied for calculating standardised uptake value.

### 5.24 Immunohistochemical staining

As previously mentioned,^41^, ^42^, ^44^, ^45^ immunohistochemistry and quantification were carried out using antibodies (1:200 or 1:100 dilution) specific for either Ki‐67 (ab15580) or CD31 (ab9498, Abcam Inc.).

### 5.25 Tumour specimens

Human specimen research was granted by the Institutional Review Board of Union Hospital, Tongji Medical College. Every research was adherent to the Declaration of Helsinki's recommendations. Written permission was acquired from each patient's legal guardian. Individuals who had undertaken radiation therapy or chemotherapy prior to surgery were not included. Normal human dorsal root ganglia were taken from prematurely ended pregnancies. All pathologically verified tissues were solidified via liquid nitrogen, and preserved at −80°C.

### 5.26 Statistical analysis

The data are presented as mean ± standard error of the mean. Statistical tests were two‐sided and included the following: Fisher's exact test for overlap analysis, Student's *t* test, analysis of variance and chi‐squared analysis for comparing difference, Pearson's correlation coefficient for gene expression association, and log‐rank test for survival difference assessment (average expression levels as cutoff values).

## AUTHOR CONTRIBUTIONS

Jianqun Wang and Mei Hong conceived and performed most of the experiments. Yang Cheng, Xiaojing Wang, Dan Li and Guo Chen accomplished some of in vitro experiments. Banghe Bao, Jiyu Song, Xinyi Du and Chunhui Yang accomplished in vivo studies. Jianqun Wang and Mei Hong undertook the mining of publicly available datasets. Qiangsong Tong and Liduan Zheng wrote the manuscript. All authors read and approved the final manuscript.

## CONFLICT OF INTEREST STATEMENT

The authors declare they have no conflicts of interest.

## ETHICS STATEMENT

Human tissue study was approved by the Institutional Review Board of Union Hospital, Tongji Medical College. All procedures were carried out in accordance with guidelines set forth by Declaration of Helsinki. All animal experiments were conducted according to the protocols approved by the Experimental Animal Ethics, Huazhong University of Science and Technology.

## CONSENT FOR PUBLICATION

All authors have agreed to publish this manuscript.

## Supporting information

Supporting Information

Supporting Information

Supporting Information

## Data Availability

Public datasets are available from Gene Expression Omnibus database (https://www.ncbi.nlm.nih.gov/geo, under accession numbers GSE62564, GSE138295, GSE10846, GSE31312, GSE16011, GSE42352) or The Cancer Genome Atlas database (https://www.cancer.gov/ccg/research/genome‐sequencing/tcga). All remaining data are presented within the article and Information Files, and available from corresponding author upon request.
