# Supplementary material for: Targeting c‐Myc transactivation by LMNA inhibits tRNA processing essential for malate‐aspartate shuttle and tumour progression
Source: Clin Transl Med. 2024 May 20;14(5):e1680. doi: 10.1002/ctm2.1680 (PMC11106511; doi:10.1002/ctm2.1680)
Supplement: Supplementary file 1 — Supporting Information [file CTM2-14-e1680-s002.docx]

**Supplementary Information**

**Targeting c-Myc transactivation by LMNA inhibits tRNA processing essential for malate-aspartate shuttle and tumor progression**

Wang et al.

**Figure S1.  *c-Myc* promotes tRNA processing gene expression in NB cells.** (**A**) Real-time qRT-PCR (normalized to *β-actin*) assay showing the relative transcript levels of *c-Myc* in normal dorsal ganglia (DG) and NB cell lines (*n*=4). (**B**) Western blot assay indicating the levels of c-Myc or MYCN in normal dorsal ganglia (DG) and NB cell lines SH-EP, SK-N-BE(2), SH-SY5Y, SK-N-SH, and SK-N-AS. (**C**) U-map of scRNA-seq results revealing the expression profiling of *EPRS* and *LARS* in varied types of cells (*n*=6442) within NB tissues. (**D**) ChIP and qPCR assays showing endogenous enrichment of c-Myc (normalized to input) on promoters of *EPRS* and *LARS* in SH-EP and SK-N-AS cells stably transfected with empty vector (mock), *c-Myc*, scramble shRNA (sh-Scb), or sh-c-Myc (*n*=5). (**E**) Dual-luciferase assay using reporters with wild-type (WT) or mutant (Mut) c-Myc binding site indicating the promoter activity of *EPRS* and *LARS* in SH-EP and SK-N-AS cells stably transfected with mock, *c-Myc*, sh-Scb, or sh-c-Myc (*n*=5). (**F**) Western blot assay showing the expression of *EPRS* and *LARS* in SH-EP and SK-N-AS cells stably transfected with mock, *c-Myc*, sh-Scb, or sh-c-Myc. (**G**) Western blot assay indicating the levels of *EPRS* and *LARS* in SH-SY5Y and SK-N-BE(2) cells stably transfected with mock, *MYCN*, sh-Scb, or sh-MYCN. ANOVA and Student’s *t* test compared the difference in **A**, **D** and **E**. ***P<*0.01, ****P<*0.001. Data are shown as mean ± s.e.m. (error bars) or representative of three independent experiments in **A**, **B** and **D**-**G**.

**Figure S2. *EPRS* and *LARS* facilitate GOT2 or MDH2 translation and MAS in NB cells.** (**A**) Expression correlation of EPRS or LARS with GOT1 and MDH1 in cancer cells (*n*=375) derived from DepMap database (https://depmap.org/portal). (**B**) Western blot assay indicating the levels of GCN2 in SH-SY5Y cells stably transfected with scramble shRNA (sh-Scb), sh-GCN2 #1, or sh-GCN2 #2. (**C**) Western blot assay showing the expression of p-GCN2, GCN2, ATF4, p-mTOR, mTOR, p-70S6K1, 70S6K1, p-4EBP1, 4EBP1, GOT1, or MDH1 in SH-SY5Y cells transfected with sh-Scb or sh-GCN2 #1, and those co-transfected with dCas9i control (dCas9i-CTL) or dCas9i-EPRS #1. (**D**) Incorporation of ^3^H-labedled proline, glutamate, or leucine into GOT1 or MDH1 protein (C) of SH-SY5Y cells stably transfected with dCas9i-CTL, dCas9i-EPRS, or dCas9i-LARS (*n*=4). (**E**) Relative mitochondrial and cytoplasmic NADH levels in SK-N-AS cells stably transfected with dCas9i-CTL, dCas9i-EPRS, or dCas9i-LARS (*n*=4). Pearson’s correlation coefficient analysis for gene expression in **A**. ANOVA compared the difference in **D** and **E**. **P<*0.05, ***P<*0.01. Data are shown as mean ± s.e.m. (error bars) or representative of three independent experiments in **B**-**E**.

**Figure S3. *c-Myc* facilitates MAS, growth, and invasion of NB cells via tRNA processing genes.** (**A**) Relative mitochondrial NADH/NAD^+^ ratio, lactate production, and ATP levels in SK-N-BE(2) cells stably transfected with empty vector (mock) or *c-Myc*, and those co-transfected with dCas9i control (dCas9i-CTL), dCas9i-EPRS #1, or dCas9i-LARS #1 (*n*=4). (**B** and **C**) Representative images of soft agar (B) and matrigel invasion (C) assays showing anchorage-independent growth and invasion of SK-N-BE(2) cells stably transfected with mock or *c-Myc*, and those co-transfected with dCas9i-CTL, dCas9i-EPRS #1, or dCas9i-LARS #1 (*n*=4). ANOVA compared the difference in **A**. ***P<*0.01. Data are shown as mean ± s.e.m. (error bars) or representative of three independent experiments in **A**-**C**.

**Figure S4. *c-Myc* promotes NB progression via tRNA processing genes *in vivo.*** (**A**) Immunohistochemical staining (upper panel, arrowheads) and quantification (lower panel) revealing the expression of Ki-67 and CD31 within xenografts in nude mice formed by subcutaneous injection of SK-N-BE(2) cells stably transfected with empty vector (mock) or *c-Myc*, and those co-transfected with dCas9i control (dCas9i-CTL), dCas9i-EPRS #1, or dCas9i-LARS #1. Scale bars: 100 μm. (**B**) Relative mitochondrial NADH/NAD^+^ ratio, lactate production, and ATP levels of xenografts in nude mice formed by subcutaneous injection of SK-N-BE(2) cells stably transfected with mock or *c-Myc*, and those co-transfected with dCas9i-CTL, dCas9i-EPRS #1, or dCas9i-LARS #1. (**C**) HE staining of lung metastatic colonies (arrowheads) of nude mice treated with tail vein injection of SK-N-BE(2) cells stably transfected with mock or *c-Myc*, and those co-transfected with dCas9i-CTL, dCas9i-EPRS #1, or dCas9i-LARS #1. Scale bars: 100 μm. ANOVA compared the difference in **A** and **B**. ***P<*0.01. Data are shown as mean ± s.e.m. (error bars) in **A** and **B**.

**Figure S5. LMNA directly interacts with c-Myc in NB cells.** (**A**) Co-IP and western blot assays showing the interaction between c-Myc and LMNA in SH-SY5Y cells transfected with full-length or truncations of HA-tagged *c-Myc* and Flag-tagged *LMNA* constructs. (**B**) Schematic illustration (upper panel) of mutation sites of *c-Myc* and *LMNA* within their corresponding BiFC constructs. Western blot assay (lower panel) showing the expression of Flag-tagged VN173 or HA-tagged VC155 in SH-SY5Y cells transfected with wild-type (WT) or mutant (Mut) BiFC constructs. (**C**) Representative images of BiFC assay indicating physical interaction of c-Myc with LMNA (arrowheads) in SK-N-BE(2) cells transfected with WT or Mut BiFC constructs, with nuclei staining with DAPI. Scale bars: 10 μm. (**D**) Co-IP and western blot assays showing the interaction between LMNA and c-Myc, p-c-Myc^S62^, or p-c-Myc^T58^ in SK-N-BE(2) and SH-SY5Y cells stably transfected with empty vector (mock), *LMNA*, scramble shRNA (sh-Scb), sh-LMNA #1, or sh-LMNA #2. Data are representative of three independent experiments in **A**-**D**.

**Figure S6.** ***LMNA* inhibits tumorigenesis and aggressiveness via repressing c-Myc activity.** (**A**) Relative mitochondrial NADH/NAD^+^ ratio, lactate production, and ATP levels of SH-SY5Y cells stably transfected with scramble shRNA (sh-Scb), sh-LMNA #1, sh-LMNA #2, or sh-c-Myc #1 (*n*=5). (**B**) Representative images (left panel) and quantification (right panel) of soft agar and matrigel invasion assays showing anchorage-independent growth and invasion of SH-SY5Y cells stably transfected with sh-Scb, sh-LMNA #1, or sh-c-Myc #1 (*n*=4). ANOVA compared the difference in **A** and **B**. ***P<*0.01. Data are shown as mean ± s.e.m. (error bars) and representative of three independent experiments in **A** and **B**.

**Figure S7. Lobeline suppresses tumorigenesis and aggressiveness of NB cells.** (**A**) Co-IP and western blot assays showing the interaction of LMNA with MYCN in SK-N-BE(2) cells treated with solvent or lobeline (LOB, 20 μmol/L) for 24 hours. (**B**) Western blot assay showing levels of LMNA, c-Myc, EPRS, LARS, GOT1, and MDH1 in SH-SY5Y cells treated with solvent or LOB as indicated. (**C**) MTT colorimetric assay indicating the viabilities of SH-SY5Y and MCF 10A cells treated with solvent or LOB as indicated. (**D**) Body weight of nude mice that received subcutaneous injection of SH-SY5Y cells and intraperitoneal administration of LOB (5 mg/kg, *n*=5 for each group). (**E**) Western blot assay showing expression of LMNA, c-Myc, EPRS, LARS, GOT1, and MDH1 within subcutaneous xenografts formed by SH-SY5Y cells in nude mice that received intraperitoneal administration of LOB (5 mg/kg, *n*=5 for each group). (**F**) Representative images (left panel) and quantification (right panel) of immunohistochemical staining indicating the expression of Ki-67 and CD31 (arrowheads) within subcutaneous xenografts formed by SH-SY5Y cells in nude mice that received intraperitoneal administration of LOB (5 mg/kg, *n*=5 for each group). Scale bars: 100 μm. ANOVA and Student’s *t* test compared the difference in **C**, **D** and **F**. **P<*0.05, ***P<*0.01, ****P<*0.001. NS, non-significant. Data are shown as mean ± s.e.m. (error bars) or representative of three independent experiments in **A**-**C**.

**Figure S8. Lobeline inhibits MAS by facilitating interaction of LMNA with c-Myc.** (**A** and **B**) ChIP and qPCR (A, normalized to input) and dual-luciferase (B) assays showing the c-Myc enrichment and promoter activity of tRNA processing genes *EPRS* and *LARS* in SH-EP and SH-SY5Y cells stably transfected with empty vector (mock), *c-Myc*, scramble shRNA (sh-Scb), or sh-LMNA #1, and those treated with lobeline (LOB, 20 μmol/L) for 24 hrs (*n*=5). (**C**) Western blot assay indicating the expression of *EPRS* and *LARS* in SH-EP and SH-SY5Y cells stably transfected with mock, *c-Myc*, sh-Scb, or sh-LMNA #1, and those treated with LOB (20 μmol/L) for 24 hrs (*n*=5). (**D**) Relative mitochondrial and cytoplasmic NADH levels in SH-EP and SH-SY5Y cells stably transfected with mock, *c-Myc*, sh-Scb, or sh-LMNA #1, and those treated with LOB (20 μmol/L) for 24 hrs (*n*=5). ANOVA compared the difference in **A**, **B**, and **D**. * *P*<0.05, ** *P*<0.01. Data are shown as mean ± s.e.m. (error bars) or representative of three independent experiments in **A**-**D**.

**Figure S9. Lobeline inhibits MAS and tumor progression via *c-Myc* and *LMNA*.** (**A** and **B**) Relative mitochondrial NADH/NAD^+^ ratio, lactate production, and ATP levels in SH-EP (A) and SH-SY5Y (B) cells stably transfected with empty vector (mock), *c-Myc*, scramble shRNA (sh-Scb), or sh-LMNA #1, and those treated with lobeline (LOB, 20 μmol/L) for 24 hrs (*n*=5). (**C**) Representative images (upper panel) and quantification (lower panel) of soft agar and matrigel invasion assays showing anchorage-independent growth and invasion of SH-EP and SH-SY5Y cells stably transfected with mock, *c-Myc*, sh-Scb, or sh-LMNA #1, and those treated with LOB (20 μmol/L) for 24 hrs (*n*=5). ANOVA compared the difference in **A**-**C**. ***P<*0.01. Data are shown as mean ± s.e.m. (error bars) or representative of three independent experiments in **A**-**C**.

**Figure S10. Kaplan-Meier curves of *c-Myc* and *LMNA* in multiple cancers.** Kaplan-Meier curves indicating the survival of patients with low or high expression of *c-Myc* or *LMNA* in B-cell lymphoma, colon carcinoma, diffuse large B-cell lymphoma, glioma, melanoma, osteosarcoma, or sarcoma derived from GEO database (GSE10846, GSE31312, GSE16011, GSE42352) or TCGA database.
