# Supplementary material for: Targeting c‐Myc transactivation by LMNA inhibits tRNA processing essential for malate‐aspartate shuttle and tumour progression
Source: Clin Transl Med. 2024 May 20;14(5):e1680. doi: 10.1002/ctm2.1680 (PMC11106511; doi:10.1002/ctm2.1680)
Supplement: Supplementary file 2 — Supporting Information [file CTM2-14-e1680-s001.docx]

**Table S1 Identification of tRNA processing genes essential for NB progression**

| ADAT1 |  | MARS2 |  | TRMT5 |  |
| --- | --- | --- | --- | --- | --- |
| AIMP1 |  | METTL1 |  | TRMT6 |  |
| ALKBH1 |  | METTL6 |  | TRMT61A |  |
| CARS |  | MOCS3 |  | TRNT1 |  |
| CDK5RAP1 |  | NARS |  | TRPT1 |  |
| CLP1 |  | NAT10 |  | TRUB2 |  |
| CTU1 |  | NSUN3 |  | TSEN15 |  |
| CTU2 |  | NSUN4 |  | TSEN2 |  |
| DARS |  | POLR3K |  | TSEN34 |  |
| DARS2 |  | POP1 |  | TSEN54 |  |
| DDX1 |  | POP4 |  | TYW5 |  |
| DTD1 |  | POP7 |  | URM1 |  |
| DTD2 |  | PUS1 |  | VARS |  |
| DUS1L |  | PUS3 |  | WDR4 |  |
| DUS3L |  | PUS7 |  | YARS2 |  |
| EARS2 |  | PUS7L |  |  |  |
| ELP2 |  | PUSL1 |  |  |  |
| ELP4 |  | QARS |  |  |  |
| ELP6 |  | QRSL1 |  |  |  |
| EPRS |  | RARS |  |  |  |
| EXOSC2 |  | RPP14 |  |  |  |
| EXOSC3 |  | RPP21 |  |  |  |
| EXOSC7 |  | RPP40 |  |  |  |
| EXOSC9 |  | RPUSD1 |  |  |  |
| FARS2 |  | RPUSD4 |  |  |  |
| FARSA |  | SARS2 |  |  |  |
| FARSB |  | SSB |  |  |  |
| FTSJ1 |  | TARBP1 |  |  |  |
| GATC |  | TARS |  |  |  |
| GRSF1 |  | THG1L |  |  |  |
| GTPBP3 |  | THUMPD1 |  |  |  |
| HSD17B10 |  | THUMPD3 |  |  |  |
| IARS |  | TPRKB |  |  |  |
| IARS2 |  | TRIT1 |  |  |  |
| KARS |  | TRMT1 |  |  |  |
| LAGE3 |  | TRMT10A |  |  |  |
| LARS |  | TRMT10C |  |  |  |
| LARS2 |  | TRMT112 |  |  |  |
| LCMT2 |  | TRMT12 |  |  |  |
| LSM6 |  | TRMT44 |  |  |  |

**Table S2 Identification of EPRS/LARS-correlated MAS proteins**

| **MAS protein** |  | **EPRS-correlated MAS proteins** |  | **LARS-correlated MAS proteins** |
| --- | --- | --- | --- | --- |
| GOT1 |  | GOT1 |  | GOT1 |
| GOT2 |  | GOT2 |  | GOT2 |
| MDH1 |  | MDH1 |  | MDH1 |
| MDH2 |  | MDH2 |  | MDH2 |
| SLC25A12 |  |  |  |  |
| SLC25A13 |  |  |  |  |
| SLC25A18 |  |  |  |  |
| SLC25A22 |  |  |  |  |

**Table S3 Mass spectrometry analysis of c-Myc-interacting proteins**

| 43350 | ANXA2 | DHX9 | F6UVQ4 | H0YN42 | KPYM | PSMD5 | ST134 |
| --- | --- | --- | --- | --- | --- | --- | --- |
| 1433B | ANXA5 | DLDH | F6VDH7 | H0YNW5 | LAP2A | PTBP1 | STIP1 |
| 1433E | ARF1 | DNJA1 | F8VNW4 | H2B1C | MDH2 | PUR2 | STML2 |
| 1433F | ARF3 | DUT | F8VPF3 | H2B1D | LDHB | PUR6 | SYEP |
| 1433G | AT1A1 | DX39A | F8VQQ4 | H2B1H | LEG1 | Q32Q12 | SYNC |
| 1433S | ATPA | DX39B | F8VVM2 | H2B1K | LGUL | Q3BDU5 | SYVC |
| 1433T | ATPB | DYHC1 | F8VWS0 | H2B1L | LKHA4 | Q3KNR6 | TADBP |
| 1433Z | ATPG | E2QRB9 | F8VXJ7 | H2B1M | LMNA | Q51\|A0A1W2PQ51 | TAGL2 |
| 1A01 | ATPO | E5RFV2 | F8VY04 | H2B1N | LMNB2 | Q5JP53 | TALDO |
| 1A03 | B0QYK0 | E5RH77 | F8VYE8 | H2B2F | LPPRC | Q5JR05 | TBA1A |
| 1A11 | B1AHC9 | E5RI99 | F8VZJ2 | H2BFS | LRC59 | Q5JR07 | TBA1B |
| 1A36 | B2R5W2 | E5RJD2 | F8VZX2 | H3BPE7 | M0QXS5 | Q5JR08 | TBA1C |
| 2AAA | B3KQV6 | E5RJG7 | F8W031 | H3BR70 | M0R0P7 | Q5SRN5 | TBA3C |
| A0A024R4M0 | B4DJV2 | E7EMB3 | F8W0W4 | H3BRG4 | M0R117 | Q5SRN7 | TBA3E |
| A0A024RA52 | B4DT28 | E7EN95 | F8W0W8 | H3BRU6 | M0R1A7 | Q5STU3 | TBA4A |
| A0A087WSW9 | B4DUC8 | E7ENZ3 | F8W1A4 | H3BSJ9 | M0R3D6 | Q5T6W2 | TBB4B |
| A0A087WSY9 | B4DUR8 | E7EPK1 | F8W1N5 | H3BT71 | MAP4 | Q5T7C4 | TBB5 |
| A0A087WTP3 | B4DY08 | E7EQR4 | F8W1R7 | H3BTN5 | MATR3 | QCR2 | TCPA |
| A0A087WTT1 | B4DY09 | E7EQV3 | F8W6I7 | H7BZ94 | MCM4 | R4GNH3 | TCPB |
| A0A087WUK2 | B4E3S0 | E7ERJ7 | F8W809 | H7C2I1 | MDHC | RAB10 | TCPD |
| A0A087WUZ3 | B5MCP9 | E7ES33 | F8W914 | H7C3I1 | MDHM | RAB1B | TCPE |
| A0A087WVQ6 | B5MD38 | E7ETZ0 | F8WB63 | H7C540 | MIC60 | RAB1C | TCPG |
| A0A087WX29 | B5MDF5 | E7EVA0 | FABP5 | H9KV75 | ML12A | RAB7A | TCPH |
| A0A087WYC1 | B7Z645 | E7EX29 | FAS | HCD2 | ML12B | RACK1 | TCPQ |
| A0A087WYT3 | B7Z6Z4 | E9PBS1 | FKBP4 | HMGB1 | MPCP | RAN | TCPZ |
| A0A087X0X3 | B7ZAR1 | E9PCA1 | FLNA | HMGB2 | MPPB | RAP1A | TEBP |
| A0A087X1X7 | B8ZZJ2 | E9PCY7 | FLNB | HNRDL | MTAP | RAP1B | TERA |
| A0A087X2I1 | B8ZZL8 | E9PDE8 | FSCN1 | HNRH1 | MTPN | RBMX | TFR1 |
| A0A0A0MR02 | B9A067 | E9PEB5 | FUBP1 | HNRPC | MYH9 | RHOA | THIL |
| A0A0A0MRM9 | BASP1 | E9PEX6 | FUBP2 | HNRPD | MYL6 | RHOC | TIF1B |
| A0A0A0MSI0 | C1QBP | E9PIR7 | FUMH | HNRPF | NACA | RINI | TKT |
| A0A0A0MTS2 | C1TC | E9PIX6 | FUS | HNRPK | NACAM | RL12 | TLN1 |
| A0A0B4J1R6 | C9J592 | E9PK01 | G3P | HNRPL | NASP | RL13 | TOM34 |
| A0A0C4DG17 | C9J9K3 | E9PK25 | G3V0E4 | HNRPM | NOLC1 | RL18A | TPIS |
| A0A0C4DGI3 | C9JDL2 | E9PKD5 | G3V0E5 | HNRPQ | NPM | RL28 | TPM4 |
| A0A0C4DGL3 | C9JEV8 | E9PKE3 | G3V1A4 | HNRPR | NUCL | RL30 | TPR |
| A0A0G2JI36 | C9JFL5 | E9PKG1 | G3V1Q4 | HNRPU | NUDT5 | RL4 | TRAP1 |
| A0A0G2JIF2 | C9JIS1 | E9PKZ0 | G3V1V0 | HS71A | OAT | RL5 | TRXR1 |
| A0A0G2JIW1 | C9JJQ8 | E9PM69 | G3V2Q1 | HS71B | ODO2 | RL6 | TWF2 |
| A0A0G2JL56 | C9JNR4 | E9PMD8 | G3V4C1 | HS74L | P5CS | RL7A | TXD17 |
| A0A0R4J2E8 | C9JQ00 | E9PP50 | G3V4W0 | HS90A | PA2G4 | RL8 | U3KQK0 |
| A0A0U1RQF0 | C9JX21 | E9PQ98 | G3V576 | HS90B | PABP1 | RL9 | U520 |
| A0A0U1RRM4 | C9JXA5 | E9PQD7 | G6PI | HSP74 | PARK7 | RLA0 | UAP1 |
| A0A140T936 | CALM1 | E9PQH6 | G8JLA2 | HSP7C | PCBP1 | RLA2 | UBA1 |
| A0A182DWI3 | CALM2 | E9PRY8 | G8JLB6 | I3L0K7 | PCBP2 | ROA1 | UBP14 |
| A0A1W2PQD0 | CALM3 | ECHA | GARS | I3L397 | PCNA | ROA2 | V9GYZ6 |
| A0A1W2PQM2 | CALR | ECHB | GBB2 | I3L3B0 | PDIA1 | ROA3 | VAT1 |
| A0A1W2PS24 | CALX | ECHM | GDIB | I3L3Q7 | PDIA3 | ROAA | VDAC1 |
| A0A1W2PSE7 | CAND1 | EF1A1 | GLRX1 | I3L504 | PDIA4 | RPN1 | VDAC2 |
| A6NCQ0 | CAP1 | EF1B | GLYM | IF4A1 | PDIA6 | RPN2 | VDAC3 |
| A6NFX8 | CBR1 | EF1D | GRP75 | IF5A1 | PEBP1 | RS14 | X6RJP6 |
| A6NIW5 | CDC37 | EF1G | GRP78 | ILF2 | PGAM1 | RS19 | XPO2 |
| A6NJA2 | CH10 | EF2 | GSTO1 | ILF3 | PGK1 | RS2 | XRCC5 |
| A6NJU6 | CH60 | EFTU | GSTP1 | IMB1 | PHB | RS3 | XRCC6 |
| A6NL76 | CISY | EIF3A | H0Y2Y8 | IPYR | PHB2 | RS4X | YBOX1 |
| A6NLN1 | CLH1 | EIF3B | H0Y449 | ITB1 | PLIN3 | RS6 | ZYX |
| A8MVZ9 | CLIC1 | EIF3E | H0Y6E7 | J3KND3 | PLSL | RS7 |  |
| A8MXP9 | CLIC4 | ENOA | H0Y7A7 | J3KPE3 | PNPH | RS8 |  |
| AATM | CNPY2 | ENOG | H0Y8G5 | J3KPX7 | PP1A | RS9 |  |
| ACLY | COF1 | ENPL | H0Y9V9 | J3KQE5 | PP1B | RSSA |  |
| ACTA | COR1C | ERO1A | H0YA96 | J3KSV6 | PP1G | RTN4 |  |
| ACTC | D6R991 | ETFB | H0YB22 | J3QKP5 | PPIA | RUVB2 |  |
| ACTG | D6R9A6 | EWS | H0YEN5 | J3QRS3 | PPIB | SAHH |  |
| ACTH | D6R9P3 | EZRI | H0YH81 | K1C10 | PPIF | SCOT1 |  |
| ACTN1 | D6RAC2 | F10A1 | H0YHX9 | K1C9 | PRDX2 | SERPH |  |
| ACTN4 | D6RAF8 | F2Z393 | H0YKC5 | K22E | PRDX3 | SF3B1 |  |
| ACTS | D6RAN4 | F5GWA7 | H0YKD8 | K2C1 | PRDX5 | SF3B3 |  |
| ADT2 | D6RBZ0 | F5GWF6 | H0YL12 | K2C6B | PRDX6 | SFPQ |  |
| AHNK | D6RD18 | F5GY37 | H0YLP6 | K7EK33 | PROF1 | SMD1 |  |
| AL1B1 | D6REE5 | F5H018 | H0YLU7 | K7EKH5 | PRPS2 | SMD3 |  |
| ALDOA | D6REM6 | F5H0C8 | H0YMD0 | K7ELW0 | PRS10 | SPRE |  |
| ALDOC | D6RHH4 | F5H2F4 | H0YMF4 | K7EQ02 | PRS6A | SPTB2 |  |
| AN32A | DAZP1 | F5H3X6 | H0YMP1 | K7EQ55 | PSA1 | SRRM2 |  |
| ANM1 | DDX17 | F5H5D3 | H0YMU9 | KAD2 | PSA2 | SRSF3 |  |
| ANXA1 | DECR | F6QUT6 | H0YN26 | KINH | PSB5 | SSBP |  |

**Table S4 Primer sets used for qRT-PCR, ChIP, and RIP**

| **Primer set 1** | **Primers** | **Sequence** | **Product size (bp)** | **Application** |
| --- | --- | --- | --- | --- |
| DARS | Forward | 5'-TGCCTGACCCAAGAAATCCC-3' | 211 | qRT-PCR |
|  | Reverse | 5'-TCCAATCCAATGCCTCCACC-3' |  |  |
| EPRS | Forward | 5'-GAAAGAAGTAGCCAAACACCCA-3' | 120 | qRT-PCR |
|  | Reverse | 5'-GTAACCATCTCACCCTCCGAAA-3' |  |  |
| FARSB | Forward | 5'-CCTGTGCTCCCAAGAAGATA-3' | 119 | qRT-PCR, |
|  | Reverse | 5'-GAAGAAGGGTAGTGCGTGCC-3’ |  |  |
| LARS | Forward | 5'- ACAGACCAGCAAGGGCAAGTA-3' | 151 | qRT-PCR |
|  | Reverse | 5'-CCACCATGTTCTCCACTCGCAC-3' |  |  |
| NARS | Forward | 5'-AAGAACCTGGAAGAAGCAAA-3' | 187 | qRT-PCR |
|  | Reverse | 5'-AACCTGTACCATCTCGCAAC-3' |  |  |
| LMNA | Forward | 5'-GACTGTGGTTGAGGACGACG-3' | 212 | qRT-PCR |
|  | Reverse | 5'-AGGCAGAAGAGCCAGAGGAG-3' |  |  |
| c-Myc | Forward | 5'-TGGTCTTCCCCTACCCTCTCA-3' | 205 | qRT-PCR |
|  | Reverse | 5'-TCTTCCTCATCTTCTTGTTCC-3' |  |  |
| GAPDH | Forward | 5'-AGAAGGCTGGGGCTCATTTG-3' | 258 | qRT-PCR |
|  | Reverse | 5'-AGGGGCCATCCACAGTCTTC-3' |  |  |
| GOT1 | Forward | 5'-CAAGGAGAAGCGGGTAGGAG-3' | 196 | qRT-PCR |
|  | Reverse | 5'-GTAGCGATAGGACCGAATGT-3' |  |  |
| GOT2 | Forward | 5'-GGGGAAACCACACACCCATCTT-3' | 294 | qRT-PCR |
|  | Reverse | 5'-CCCAGGCATCCTTATCACCATC-3' |  |  |
| MDH1 | Forward | 5'-TCGCAACAGATAAAGAAGACG-3' | 191 | qRT-PCR |
|  | Reverse | 5'-TGGATTACCCACAACAATAAC-3' |  |  |
| MDH2 | Forward | 5'-TCGGGCAGCCACTTTCACTT-3' | 193 | qRT-PCR |
|  | Reverse | 5'-GCCGGAATAACTACCACATCACAA-3' |  |  |
| ACTB | Forward | 5'-TGCCCATCTACGAGGGGTATG-3' | 156 | qRT-PCR |
|  | Reverse | 5'-TCTCCTTAATGTCACGCACGATTT-3' |  | RIP |
| c-Myc-KO | Forward | 5'-TGCTCCCTTTATTCCCCCAC-3' | 333 | RT-PCR |
|  | Reverse | 5'-TTTCTTCCAGATATCCTCGC-3' |  |  |
| EPRS ChIP  (-247/-12) | Forward | 5'-CAACTCATCCCCGGGATTTC-3' | 235 | ChIP |
|  | Reverse | 5'-CTCTCTCCCCCATCCTGAGT-3' |  |  |
| LARS ChIP  (-660/-435) | Forward | 5'-CACTCCAGCCTGGAGACAGAAC-3' | 225 | ChIP |
|  | Reverse | 5'-CGCCACCACACCGACTAATTTT-3' |  |  |
| GOT1 | Forward | 5'-AGACCTGACCTGCCAGCTCC-3' | 56 | RIP |
|  | Reverse | 5'-GAGAGACTAGGAATCAAGAG-3' |  |  |
| MDH1 | Forward | 5'-CAGTTCCGCGGTAGAGGTGA-3' | 76 | RIP |
|  | Reverse | 5'-ATTGAAAACTGCGGGGACAA-3' |  |  |

DARS, aspartyl-tRNA synthetase; EPRS, glutamyl-prolyl-tRNA synthetase; FARSB, phenylalanyl-tRNA synthetase subunit beta; LARS, leucyl-tRNA synthetase; NARS, asparaginyl-tRNA synthetase; LMNA, lamin A/C; GAPDH, glyceraldehyde-3-phosphate dehydrogenase; GOT1, glutamic-oxaloacetic transaminase 1; GOT2, glutamic- oxaloacetic transaminase 2; MDH1, malate dehydrogenase 1; MDH2, malate dehydrogenase 2; ACTB, beta-actin.

**Table S5 Oligonucleotide sets used for constructs and probes**

| **Oligo Set** | **Sequences** |
| --- | --- |
| pCMV-HA-c-Myc (1-1368) | 5'-CCGGAATTCGGATGCTGGATTTTTTTCGGGT-3' (sense); |
|  | 5'-GCCGCTCGAGTTACGCACAAGAGTTCCGTA-3' (antisense) |
| pCMV-HA-c-Myc (1-960) | 5'-CCGGAATTCGGATGCTGGATTTTTTTCGGGT-3' (sense); |
|  | 5'-GCCGCTCGAGTGTGGAGACGTGGCACCTCT-3' (antisense) |
| pCMV-HA-c-Myc (1-453) | 5'-CCGGAATTCGGATGCTGGATTTTTTTCGGGT-3' (sense); |
|  | 5'-GCCGCTCGAGCCACATACAGTCCTGGATGA-3' (antisense) |
| pCMV-HA-c-Myc (454-960) | 5'-CCGGAATTCGGAGCGGCTTCTCGGCCGCCGC-3' (sense); |
|  | 5'-GCCGCTCGAGTGTGGAGACGTGGCACCTCT-3' (antisense) |
| pCMV-HA-c-Myc (454-828) | 5’-CCGGAATTCGGAGCGGCTTCTCGGCCGCCGC-3' (sense); |
|  | 5’-GCCGCTCGAGTTCCTCATCTTCTTGTTCCT-3' (antisense) |
| pCMV-HA-c-Myc (454-705) | 5’-CCGGAATTCGGAGCGGCTTCTCGGCCGCCGC-3' (sense); |
|  | 5’-GCCGCTCGAGGGACGGAGAGAAGGCGCTGG-3' (antisense) |
| pCMV-HA-c-Myc (454-573)  pCMV-HA-c-Myc (961-1368) | 5’-CCGGAATTCGGAGCGGCTTCTCGGCCGCCGC-3' (sense); |
|  | 5’-GCCGCTCGAGGCTGGAGGTGGAGCAGACGC-3' (antisense)  5’-CCGGAATTCGGCATCAGCACAACTACGCAGC-3’ (antisense) |
| pGEX-6P-1-c-Myc (1-1368) | 5’-CCGGAATTCATGCTGGATTTTTTTCGGGT-3' (sense) |
|  | 5'-GCCGCTCGAGTTACGCACAAGAGTTCCGTA-3' (antisense); |
| pGEX-6P-1-c-Myc (1-960) | 5’-CCGGAATTCATGCTGGATTTTTTTCGGGT-3' (sense); |
|  | 5'-GCCGCTCGAGTGTGGAGACGTGGCACCTCT-3' (antisense) |
| pGEX-6P-1-c-Myc (1-453) | 5'-CCGGAATTCATGCTGGATTTTTTTCGGGT-3' (sense); |
|  | 5'-GCCGCTCGAGCCACATACAGTCCTGGATGA-3' (antisense) |
| pGEX-6P-1-c-Myc (454-960) | 5'-CCGGAATTCAGCGGCTTCTCGGCCGCCGC-3' (sense); |
|  | 5'-GCCGCTCGAGTGTGGAGACGTGGCACCTCT-3' (antisense) |
| pGEX-6P-1-c-Myc (454-828) | 5'-CCGGAATTCAGCGGCTTCTCGGCCGCCGC-3' (sense); |
|  | 5'-GCCGCTCGAGTTCCTCATCTTCTTGTTCCT-3' (antisense) |
| pGEX-6P-1-c-Myc (454-705) | 5'-CCGGAATTCATGGCCTCGGGCGACACCCTCTA-3' (sense); |
|  | 5'-GCCGCTCGAGGGACGGAGAGAAGGCGCTGG-3' (antisense) |
| pGEX-6P-1-c-Myc (454-573) | 5'-CCGGAATTCATGGCCTCGGGCGACACCCTCTA-3' (sense); |
|  | 5'-GCCGCTCGAGGCTGGAGGTGGAGCAGACGC-3' (antisense) |
| pGEX-6P-1-c-Myc (961-1368) | 5'-CCGGAATTCCATCAGCACAACTACGCAGC-3' (sense); |
|  | 5'-GCCGCTCGAGTTACGCACAAGAGTTCCGTA-3' (antisense) |
| pCMV-3Tag-1-LMNA (1-1992) | 5'-CCGGAATTCATGGAGACCCCGTCCCAGCGGC-3' (sense) |
|  | 5'-CCGCTCGAGCATGATGCTGCAGTTCTGGGGG-3' (antisense) |
| pCMV-3Tag-1-LMNA (100-1149) | 5'-CCGGAATTCGACCTGCAGGAGCTCAATGATC-3' (sense) |
|  | 5'-CCGCTCGAGCTCGCCCTCCAAGAGCTTGCGG-3' (antisense) |
| pCMV-3Tag-1-LMNA (1150-1992) | 5'-CCGGAATTCGAGGAGAGGCTACGCCTGTCCC-3' (sense) |
|  | 5'-CCGCTCGAGCATGATGCTGCAGTTCTGGGGG-3' (antisense) |
| pCMV-3Tag-1-LMNA (1-1149) | 5'-CCGGAATTCATGGAGACCCCGTCCCAGCGGC-3' (sense) |
|  | 5'-CCGCTCGAGCTCGCCCTCCAAGAGCTTGCGG-3' (antisense) |
| pET-28a-LMNA (1-1992) | 5'-CCGGAATTCATGGAGACCCCGTCCCAGCGGC-3' (sense) |
|  | 5'-CCGCTCGAGTTACATGATGCTGCAGTTCTGG-3' (antisense) |
| pET-28a-LMNA (100-1149) | 5'-CCGGAATTCGACCTGCAGGAGCTCAATGATC-3' (sense); |
|  | 5'-CCGCTCGAGCTCGCCCTCCAAGAGCTTGCGG-3' (antisense) |
| pET-28a-LMNA (1150-1992) | 5'-CCGGAATTCGAGGAGAGGCTACGCCTGTCCC-3' (sense) |
|  | 5'-CCGCTCGAGTTACATGATGCTGCAGTTCTGG-3' (antisense); |
| pET-28a-LMNA (1-1149) | 5'-CCGGAATTCATGGAGACCCCGTCCCAGCGGC-3' (sense); |
|  | 5'-CCGCTCGAGCTCGCCCTCCAAGAGCTTGCGG-3' (antisense) |
| pLenti-c-Myc  CV186-LMNA | 5'-GCGCACCGGTCGCACAAGAGTTCCGTAGCTGT-3' (sense); |
|  | 5'-GCCGCTCGAGGCTGGAGGTGGAGCAGACGC-3' (antisense)  5’-GGGTCAATATGTAATTTTCAGTG-3’ (sense) |
| pGL3-c-Myc luc | 5'-CCCACGTGCATAGCACGTGCTATGCACGTGGATACCACGTGGA-3' (sense) |
|  | 5'-CATGGGGTGCACGTATCGTGCACGATACGTGCACCTATGGTGCACCTTCGA-3' (antisense); |
| pGL3-EPRS promoter (-398/+68) WT | 5'-CGGGGTACCGCCCGCCTACACCTTCTAAAGT-3' (sense); |
|  | 5'-CCCAAGCTTGGTTCCAGAGCCTGCTACCCCT-3' (antisense) |
| pGL3-EPRS promoter (-398/+68) Mut | 5'-TGCATGCTTTCTGGGTACTACCCACATAAACGCCTATCCCATCTTAAAGATGG-3' (sense); |
|  | 5'-GCGTTTATGTGGGTAGTACCCAGAAAGCATGCACTGTCTTTATGTAAAAAGAA-3' (antisense) |
| pGL3-LARS promoter (-1037/+71) WT | 5'-GGAAGATCTGCCCACCTCGGCATCCCAAAAT-3' (sense); |
|  | 5'-CCCAAGCTTCAGCAGGCGCTAACTGACTACA-3' (antisense) |
| pGL3-LARS promoter (-1037/+71) Mut | 5'-GAGGGAGGCATCGAGTTCAAGAAGGAAATCGGGATCATCCTGGCTAACAAGGT-3' (sense); |
|  | 5'-CCCGATTTCCTTCTTGAACTCGATGCCTCCCTCGGCCTCCCAAAGTGCTAGGA-3' (antisense) |
| pBiFC-c-Myc-VN173 | 5'-ATGCTGGATTTTTTTCGGGTAGTG-3' (sense) |
|  | 5'-CGCACAAGAGTTCCGTAGCTGTTC-3' (antisense) |
| pBiFC-c-Myc-VN173 Mut-1 | 5'-CACGTCTCCATACATCAGCACAACTACGCAGCGCCTCCCTC-3' (sense) |
|  | 5'-GTGCTGATGTATGGAGACGTGGCACCTCTTGAGGACCAGTG-3' (antisense) |
| pBiFC-c-Myc-VN173 Mut-2 | 5'-ACCAGCCCCAAGTCCTCGGACACCGAGGAGAATGTCAAGAG-3' (sense) |
|  | 5'-GTCCGAGGACTTGGGGCTGGTGCATTTTCGGTTGTTGCTGA-3' (antisense) |
| pBiFC-LMNA-VC155 | 5'-ATGGAGACCCCGTCCCAGCGGCGC-3' (sense) |
|  | 5'-CATGATGCTGCAGTTCTGGGGGCT-3' (antisense) |
| pBiFC-LMNA-VC155 Mut | 5'-TGAGGATGGAAATGACCTGCTCCATCACCACCACGGCTCCC-3' (sense) |
|  | 5'-AGCAGGTCATTTCCATCCTCATCCTCGTCGTCCTCAACCAC-3' (antisense) |
| pBiFC-LMNC-VC155 | 5'-ATGGAGACCCCGTCCCAGCGGCGC-3' (sense) |
|  | 5'-GCGGCGGCTACCACTCACGTGGTGGTGATGGAGCAGGTCAT-3' (antisense) |
| tRNA-Glu-CTC-1 | 5'-AGAGCGCCGAATCCTAACCACTAGACCACCAGGGA-3' |
| tRNA-Pro-TGG-3 | 5'-CCAAAGCGAGAATCATACCCCTAGACCAACGAGCC-3' |
| tRNA-Leu-TAG-1 | 5'-AAATCCAGCGCCTTAGACCGCTCGGCCACGCTACC-3' |

LMNA, lamin A/C; EPRS, glutamyl-prolyl-tRNA synthetase; LARS, leucyl-tRNA synthetase.

**Table S6 Oligonucleotide sets used for short hairpin RNAs and CRISPR-dCas9**

| **Oligo Set** | **Sequences** |
| --- | --- |
| sh-c-Myc #1 | 5'-CCGGCCCAAGGTAGTTATCCTTAAACTCGAGTTTAAGGATAACTACCTTGGGTTTTTG-3' (Sense) |
|  | 5'-AATTCAAAAACCCAAGGTAGTTATCCTTAAACTCGAGTTTAAGGATAACTACCTTGGG-3' (Antisense) |
| sh-c-Myc #2 | 5'-CCGGCAGTTGAAACACAAACTTGAACTCGAGTTCAAGTTTGTGTTTCAACTGTTTTTG-3' (Sense) |
|  | 5'-AATTCAAAAACAGTTGAAACACAAACTTGAACTCGAGTTCAAGTTTGTGTTTCAACTG-3' (Antisense) |
| sh-MYCN #1 | 5'-CCGGGCCAGTATTAGACTGGAAGTTCTCGAGAACTTCCAGTCTAATACTGGCTTTTTG-3' (Sense) |
|  | 5'-AATTCAAAAAGCCAGTATTAGACTGGAAGTTCTCGAGAACTTCCAGTCTAATACTGGC-3' (Antisense) |
| sh-MYCN #2 | 5'-CCGGCAGCAGCAGTTGCTAAAGAAACTCGAGTTTCTTTAGCAACTGCTGCTGTTTTTG-3' (Sense) |
|  | 5'-AATTCAAAAACAGCAGCAGTTGCTAAAGAAACTCGAGTTTCTTTAGCAACTGCTGCTG-3' (Antisense) |
| sh-GCN2 #1 | 5'-CCGGTGGCCTACCGCACCATGATGCTCGAGCATCATGGTGCGGTAGGCCTTTTTG-3' (Sense) |
|  | 5'-GATCCAAAAAGGCCTACCGCACCATGATGCTCGAGCATCATGGTGCGGTAGGCCA-3' (Antisense) |
| sh-GCN2 #2 | 5'-CCGGTGAACTTCTGGAGTGTGCATCTCGAGATGCACACTCCAGAAGTTCTTTTTG-3' (Sense) |
|  | 5'-GATCCAAAAAGAACTTCTGGAGTGTGCATCTCGAGATGCACACTCCAGAAGTTCA-3' (Antisense) |
| sh-LMNA #1 | 5'-CCGGCCCACCAAAGTTCACCCTGAACTCGAGTTCAGGGTGAACTTTGGTGGGTTTTTG-3' (Sense) |
|  | 5'-GATCCAAAAACCCACCAAAGTTCACCCTGAACTCGAGTTCAGGGTGAACTTTGGTGGG-3' (Antisense) |
| sh-LMNA #2 | 5'-CCGGCGACTGGTGGAGATTGACAATCTCGAGATTGTCAATCTCCACCAGTCGTTTTTG-3' (Sense) |
|  | 5'-GATCCAAAAACGACTGGTGGAGATTGACAATCTCGAGATTGTCAATCTCCACCAGTCG-3' (Antisense) |
| dCas9i-LARS #1 | 5'-CACCGTGAGCGAGAAGAATCCGCAG-3' (Sense) |
|  | 5'-AAACCTGCGGATTCTTCTCGCTCAC-3' (Antisense) |
| dCas9i-LARS #2 | 5'-CACCGGATTTGCACAGTCGGCTGGG-3' (Sense) |
|  | 5'-AAACCCCAGCCGACTGTGCAAATCC-3' (Antisense) |
| dCas9i-EPRS #1 | 5'-CACCGCGTACTGACAGGTGGACCAG-3' (Sense) |
|  | 5'-AAACCTGGTCCACCTGTCAGTACGC-3' (Antisense) |
| dCas9i-EPRS #2 | 5'-CACCGCCGAGACCAATGACCTGTGT-3' (Sense) |
|  | 5'-AAACACACAGGTCATTGGTCTCGGC-3' (Antisense) |

GCN2, general control nonrepressed 2; LMNA, lamin A/C; LARS, leucyl-tRNA synthetase; EPRS, glutamyl-prolyl-tRNA synthetase.
