# Supplementary figures and images for: Targeting c‐Myc transactivation by LMNA inhibits tRNA processing essential for malate‐aspartate shuttle and tumour progression
Source: Clin Transl Med. 2024 May 20;14(5):e1680. doi: 10.1002/ctm2.1680 (PMC11106511; doi:10.1002/ctm2.1680)

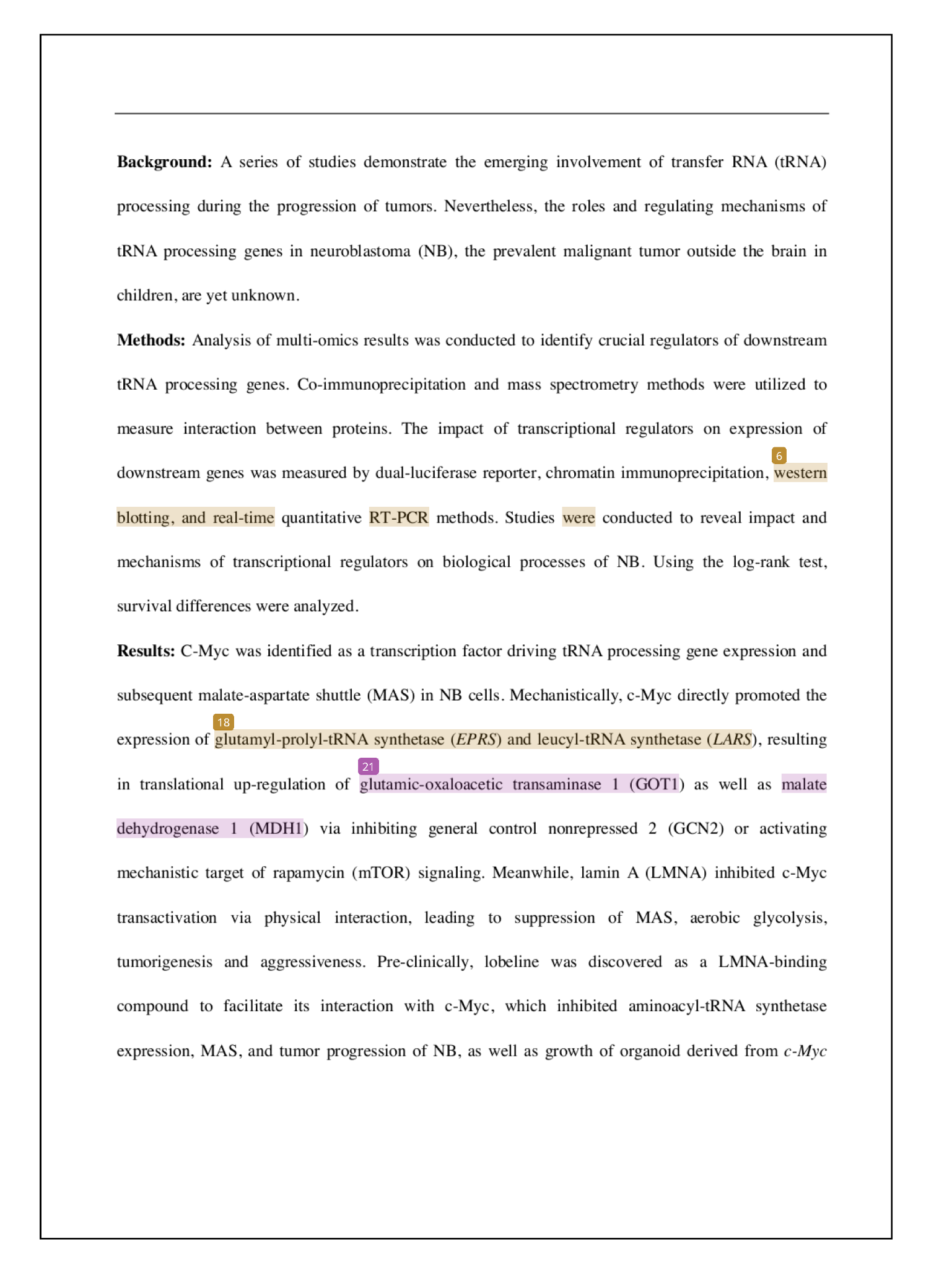


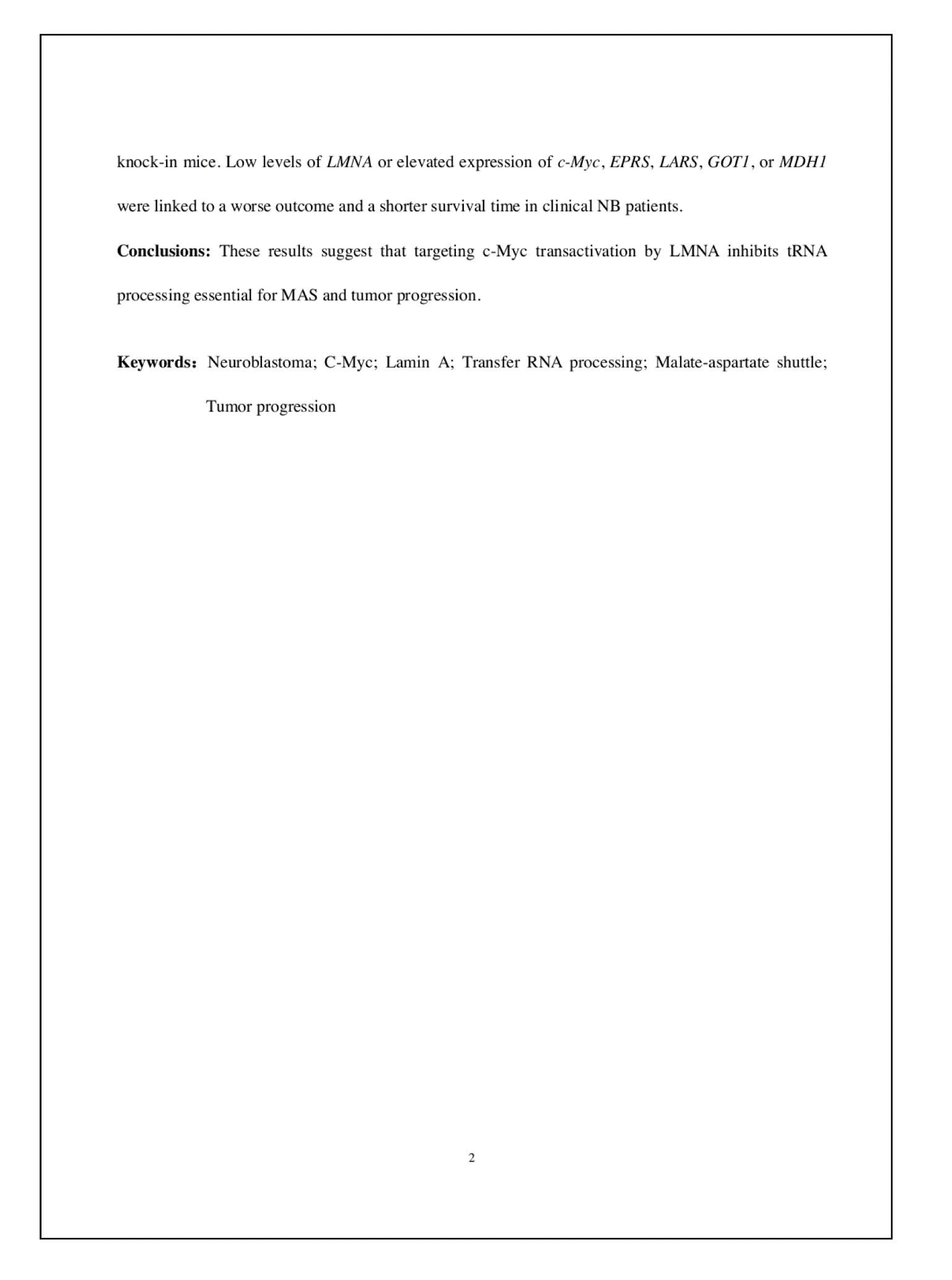


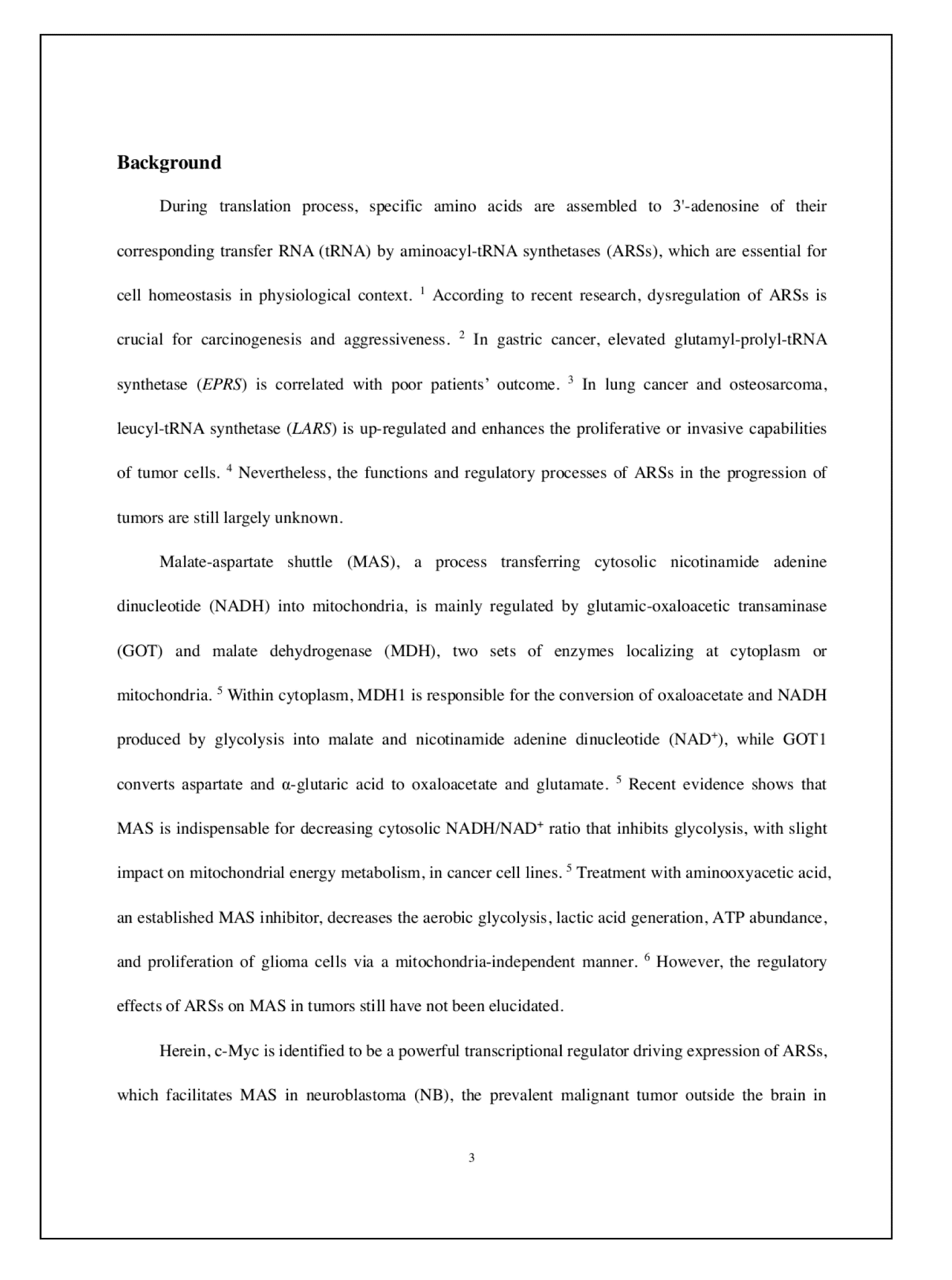


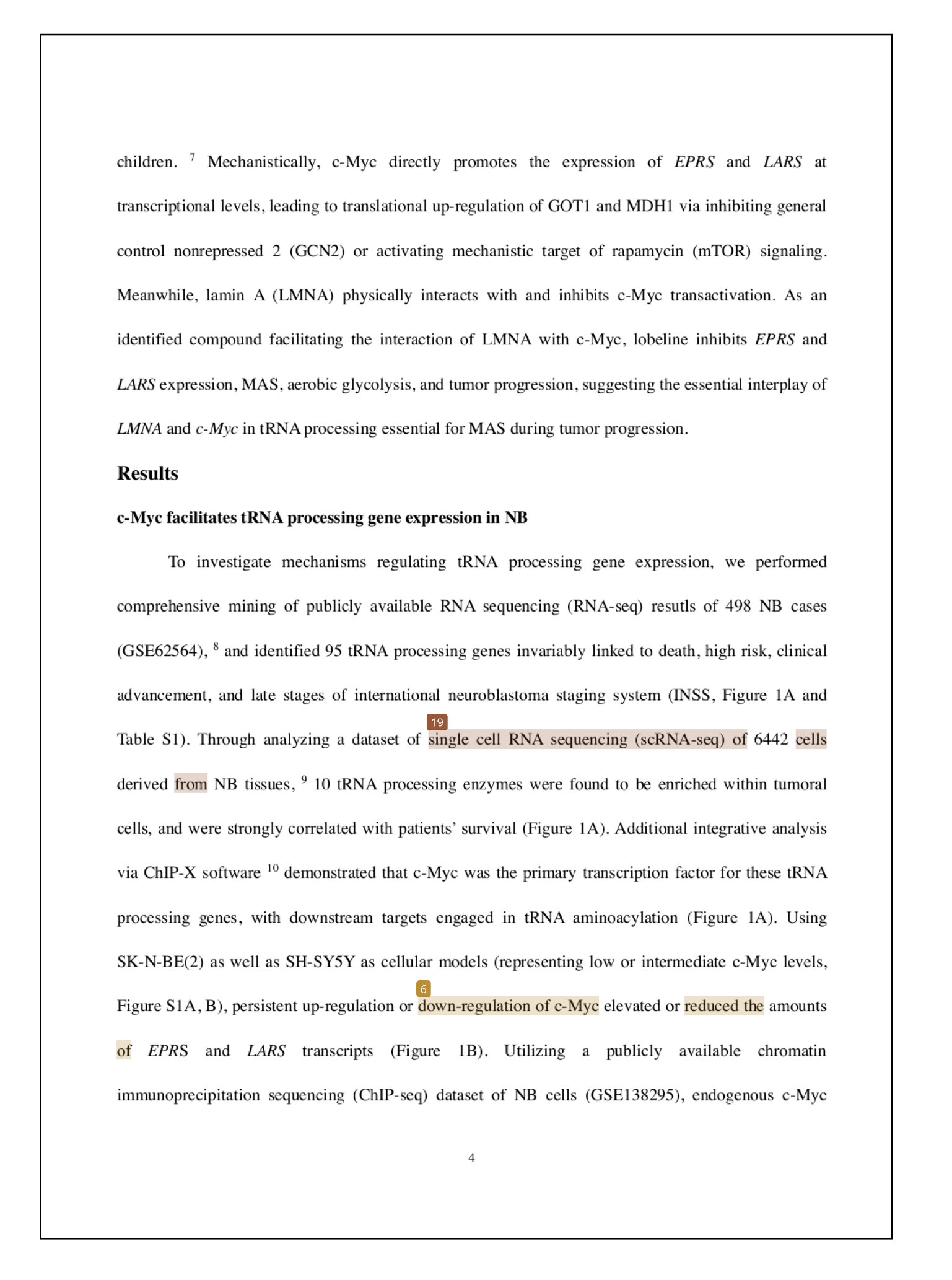


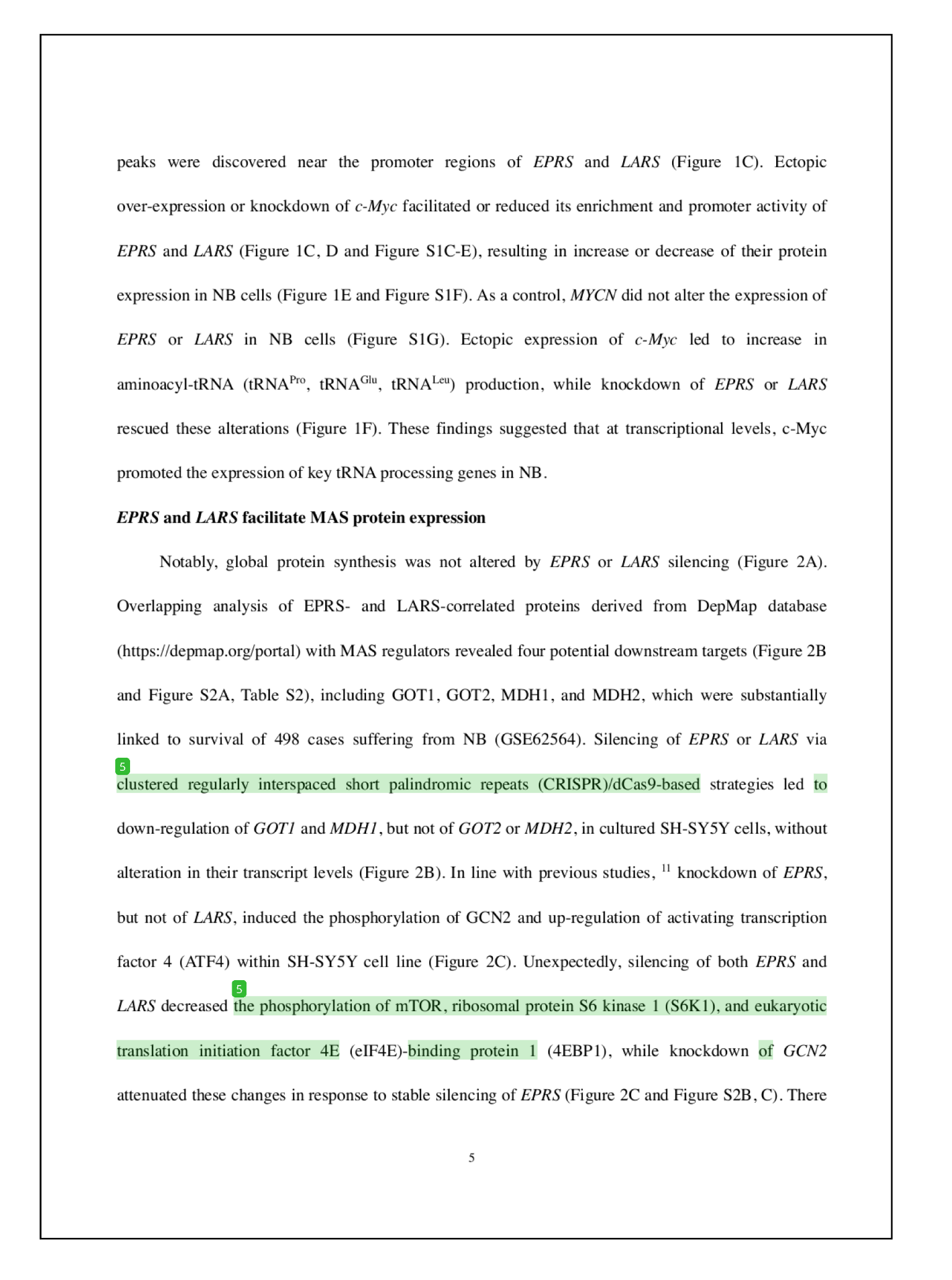


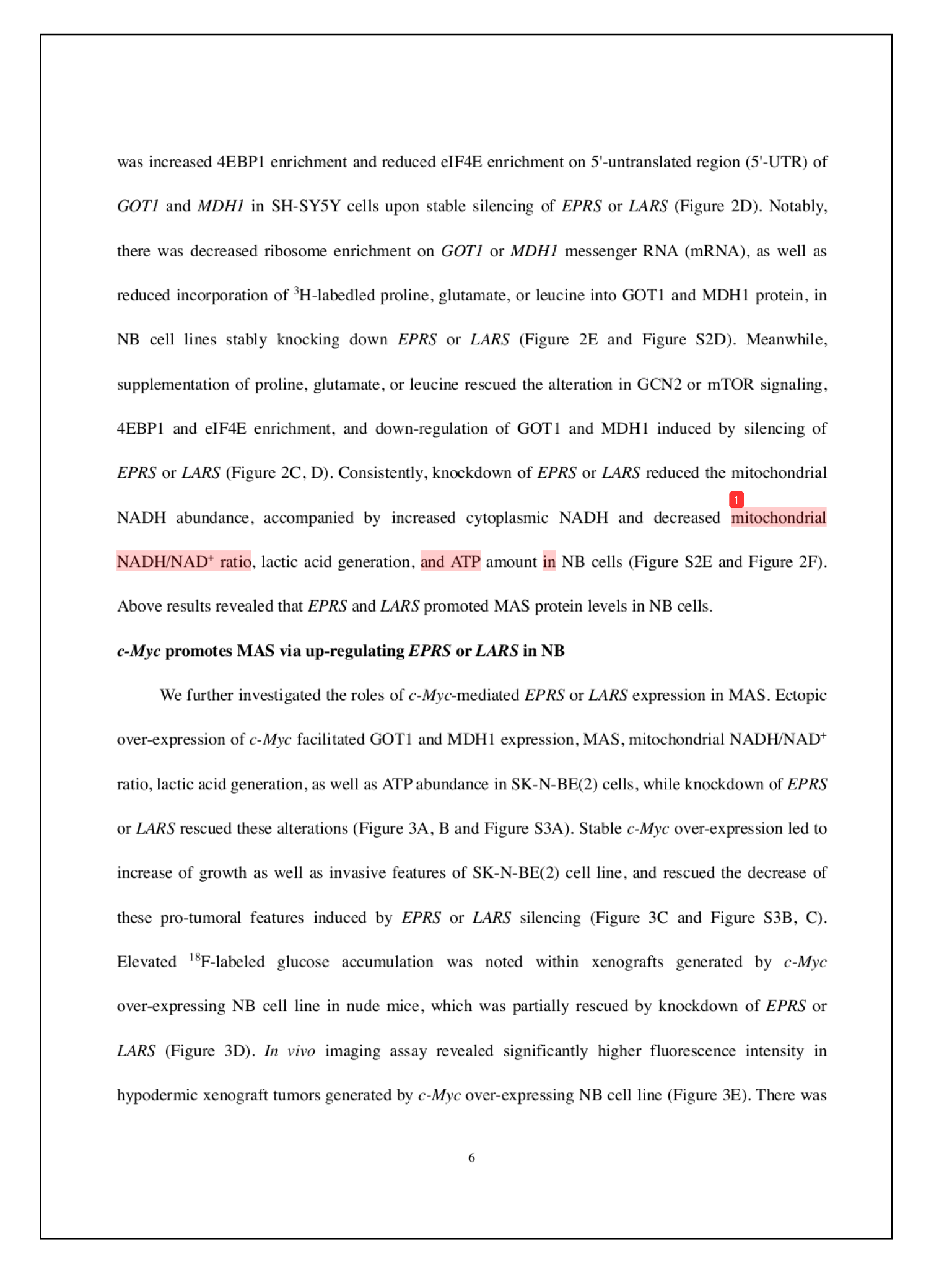


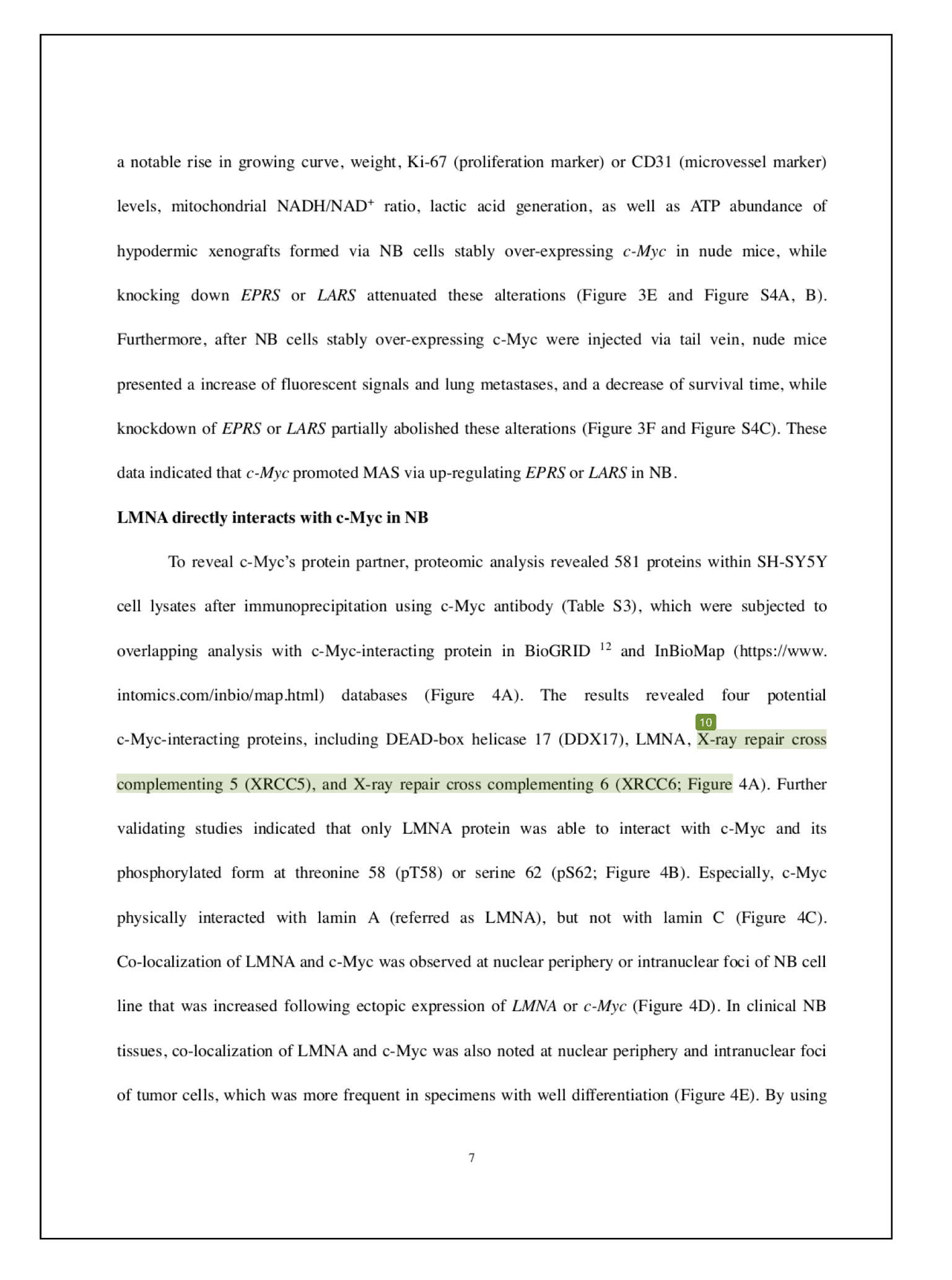


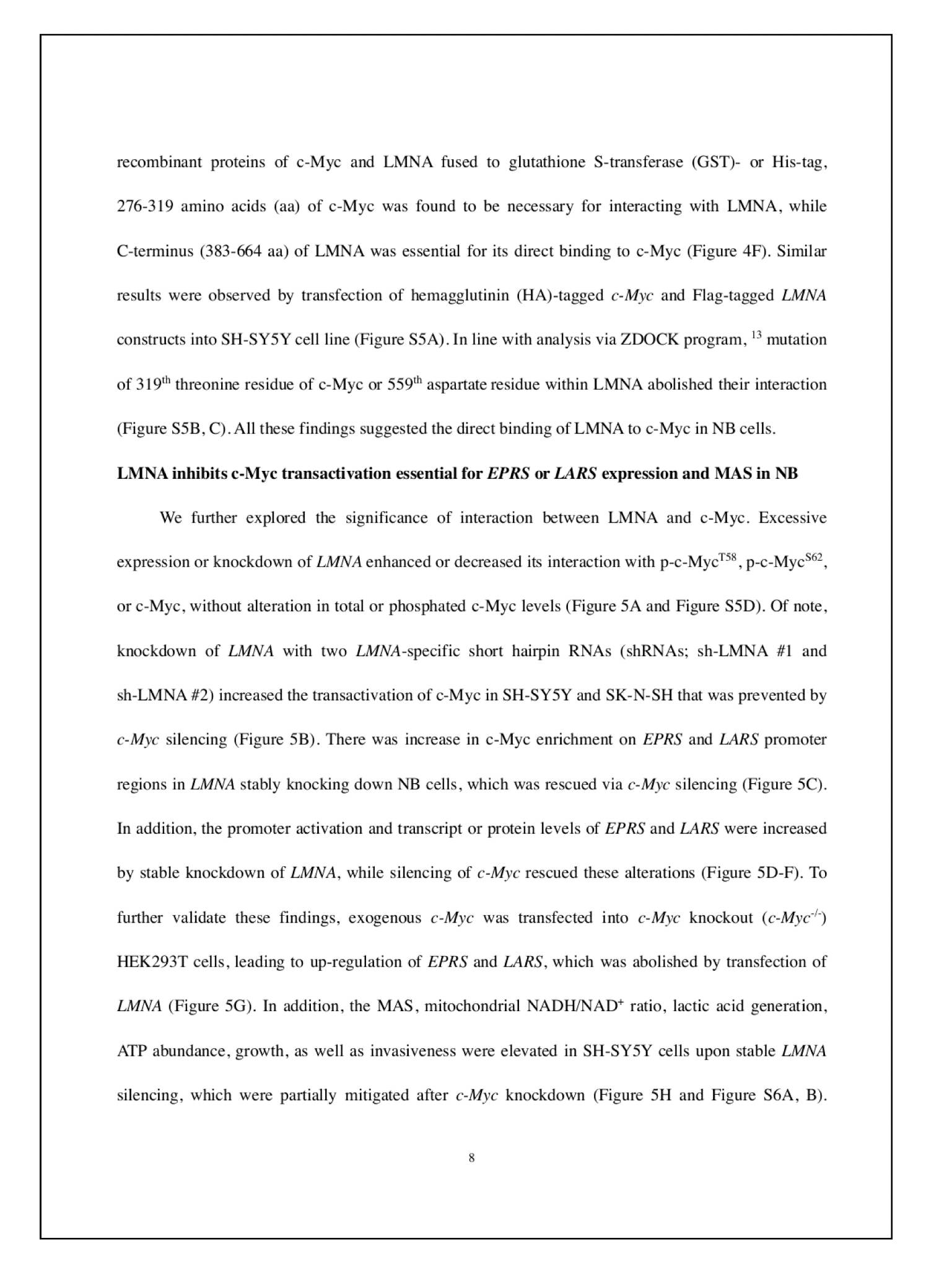


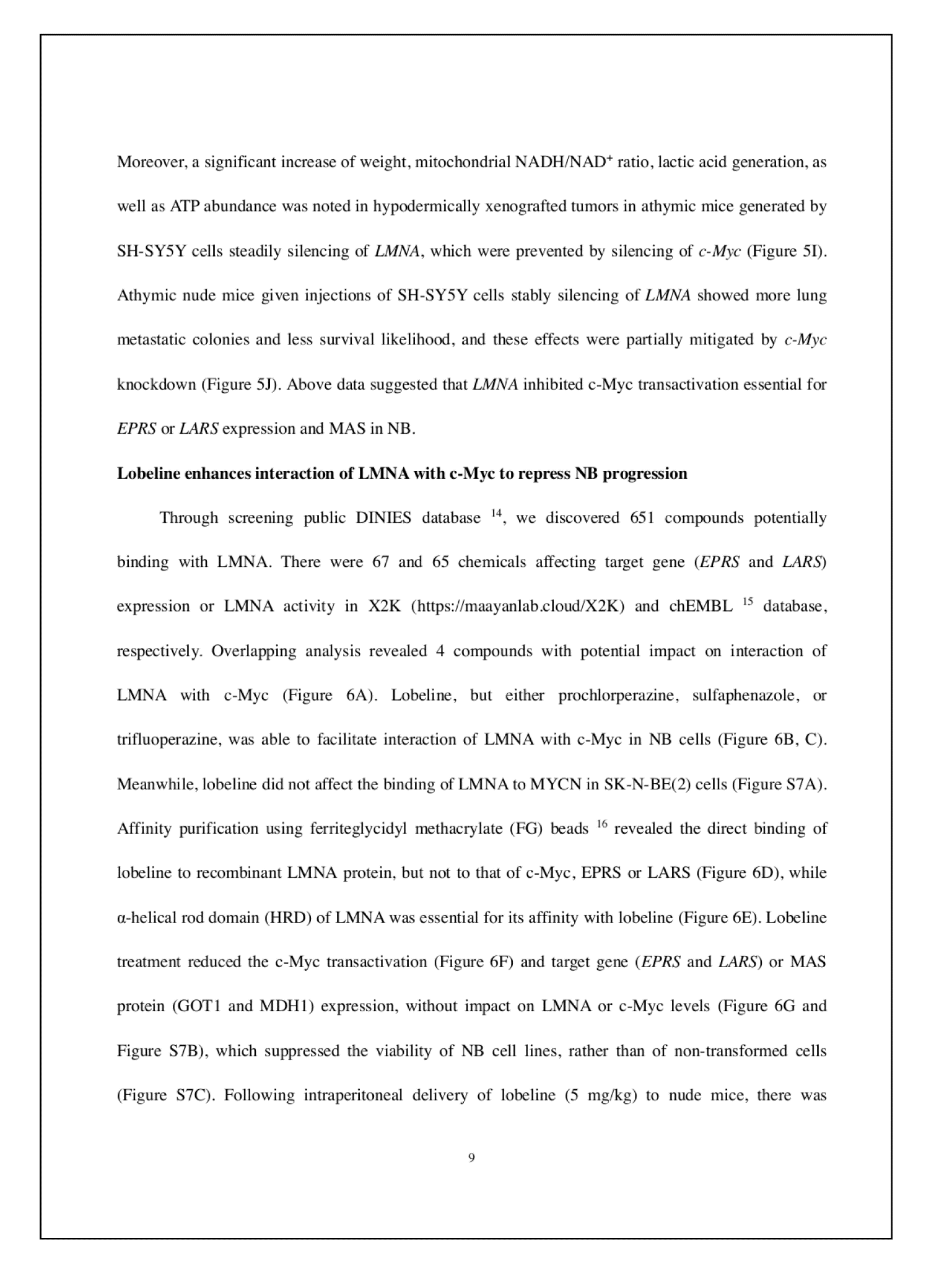


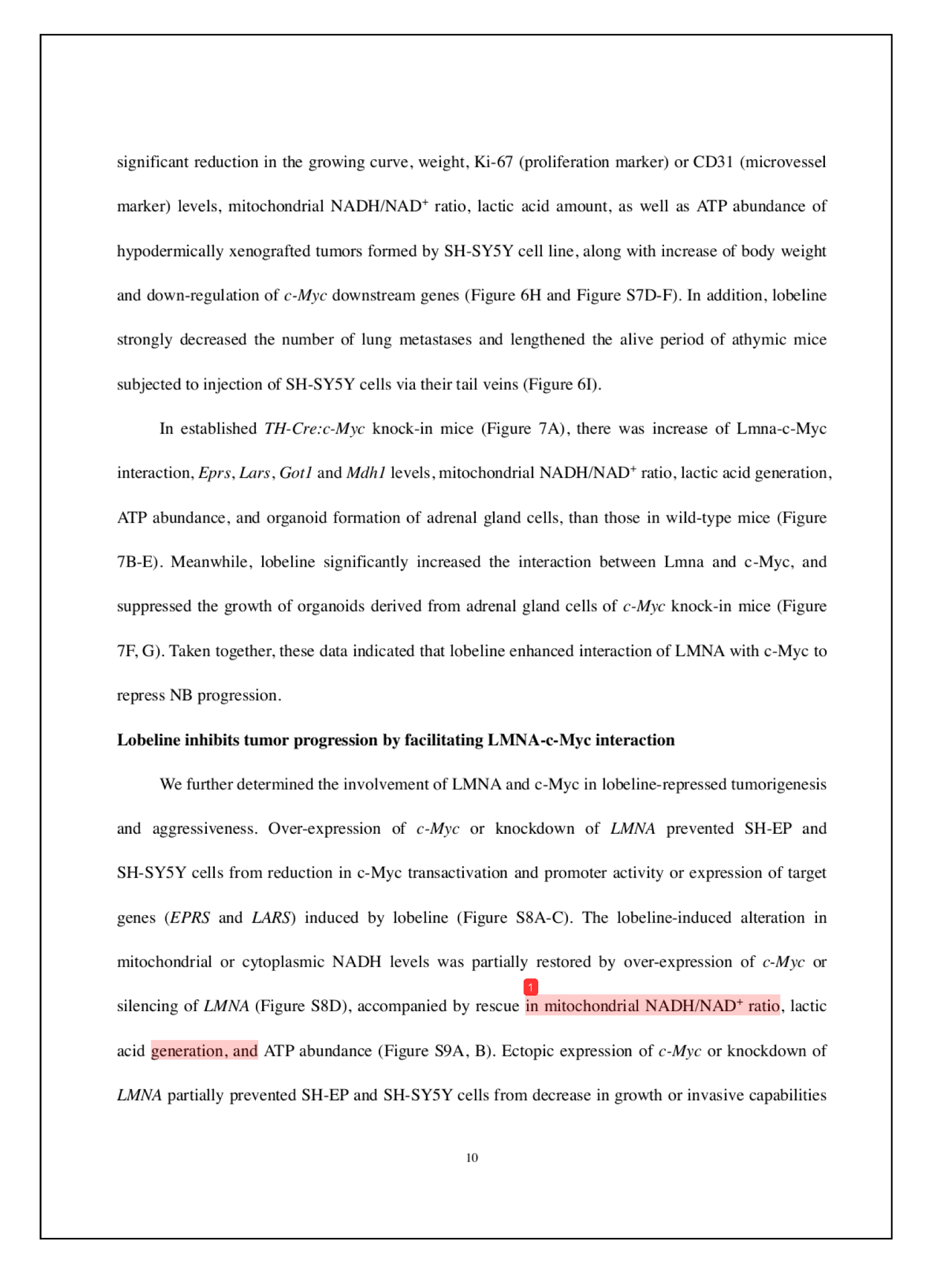


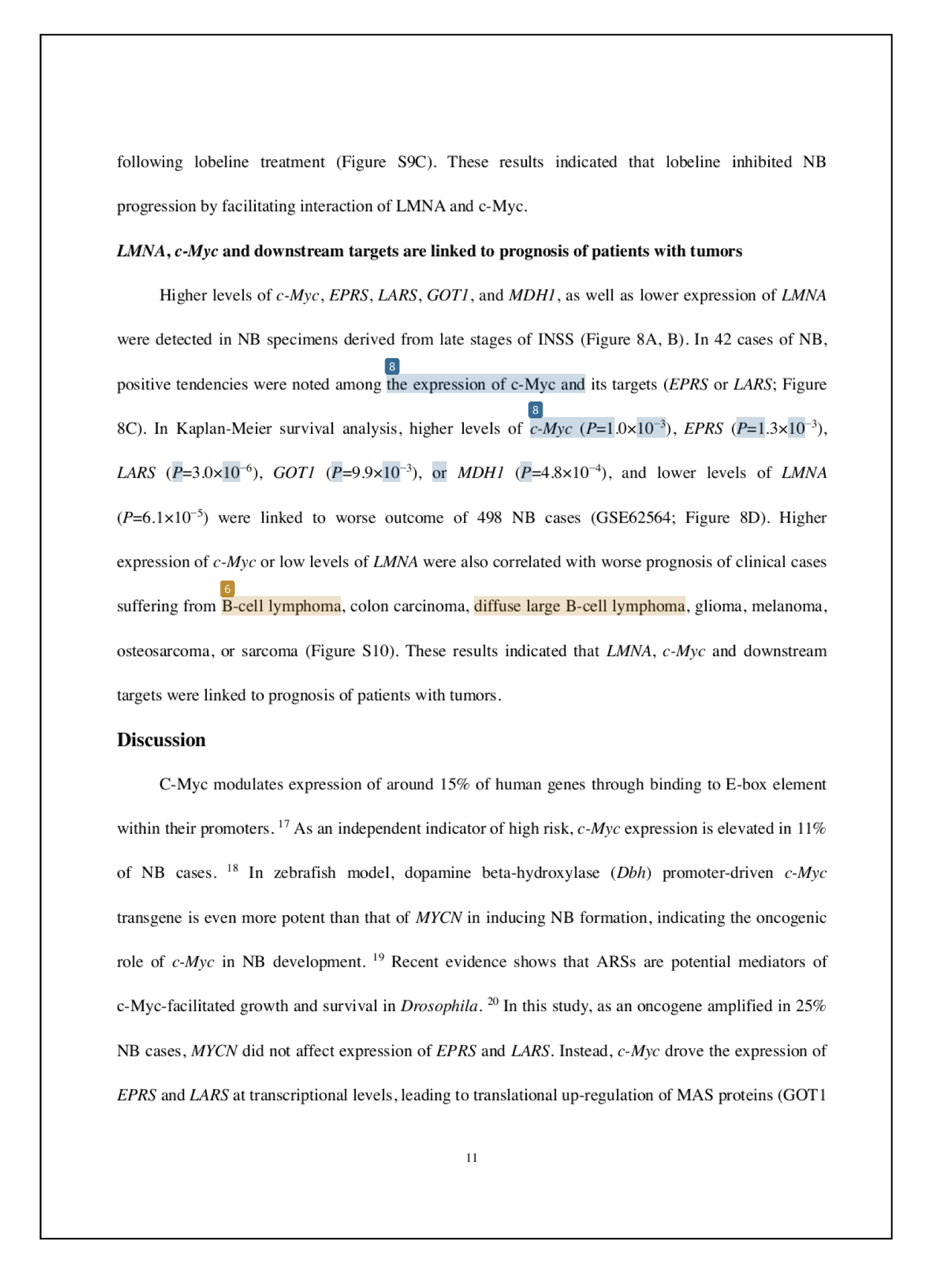


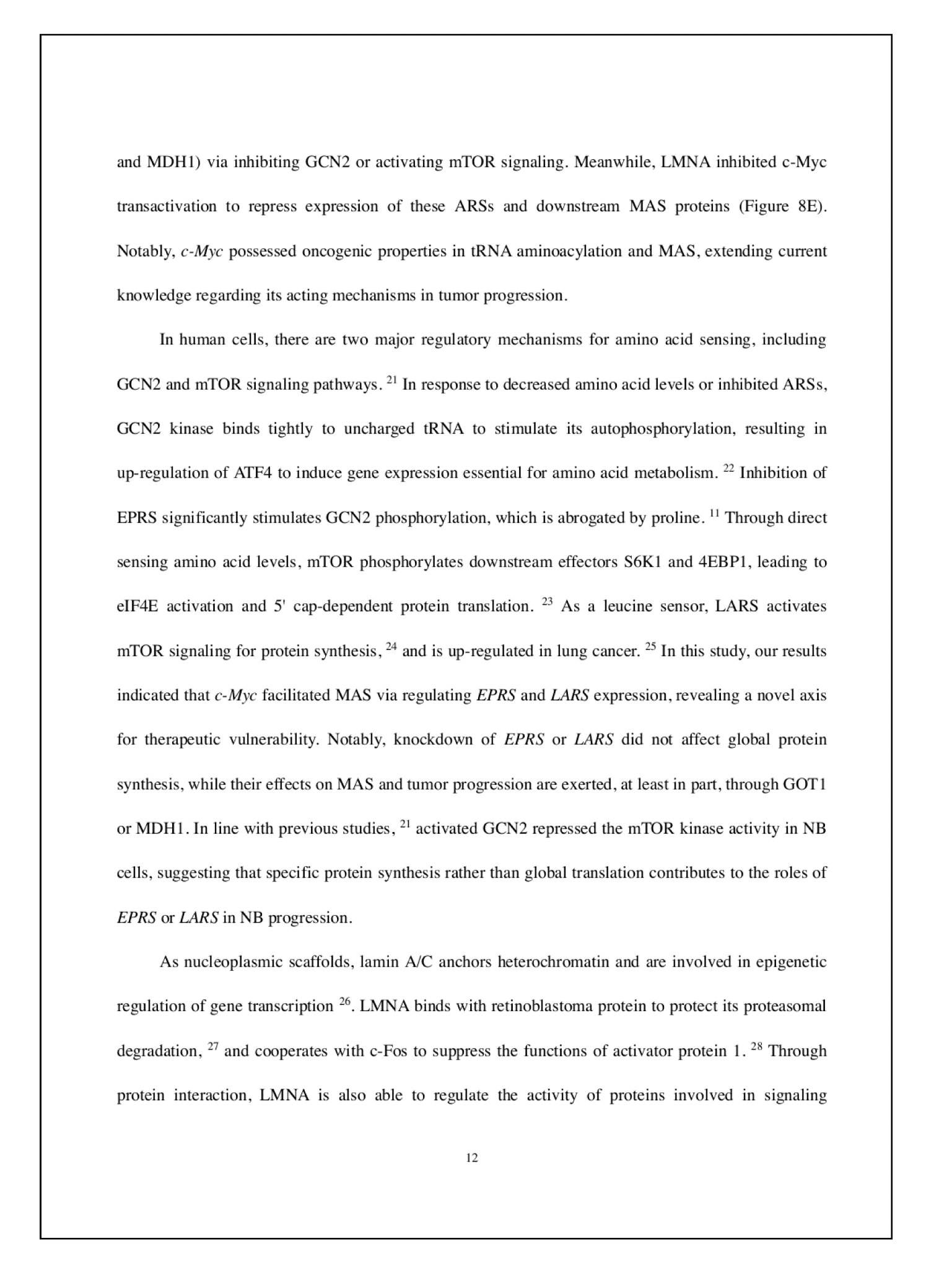


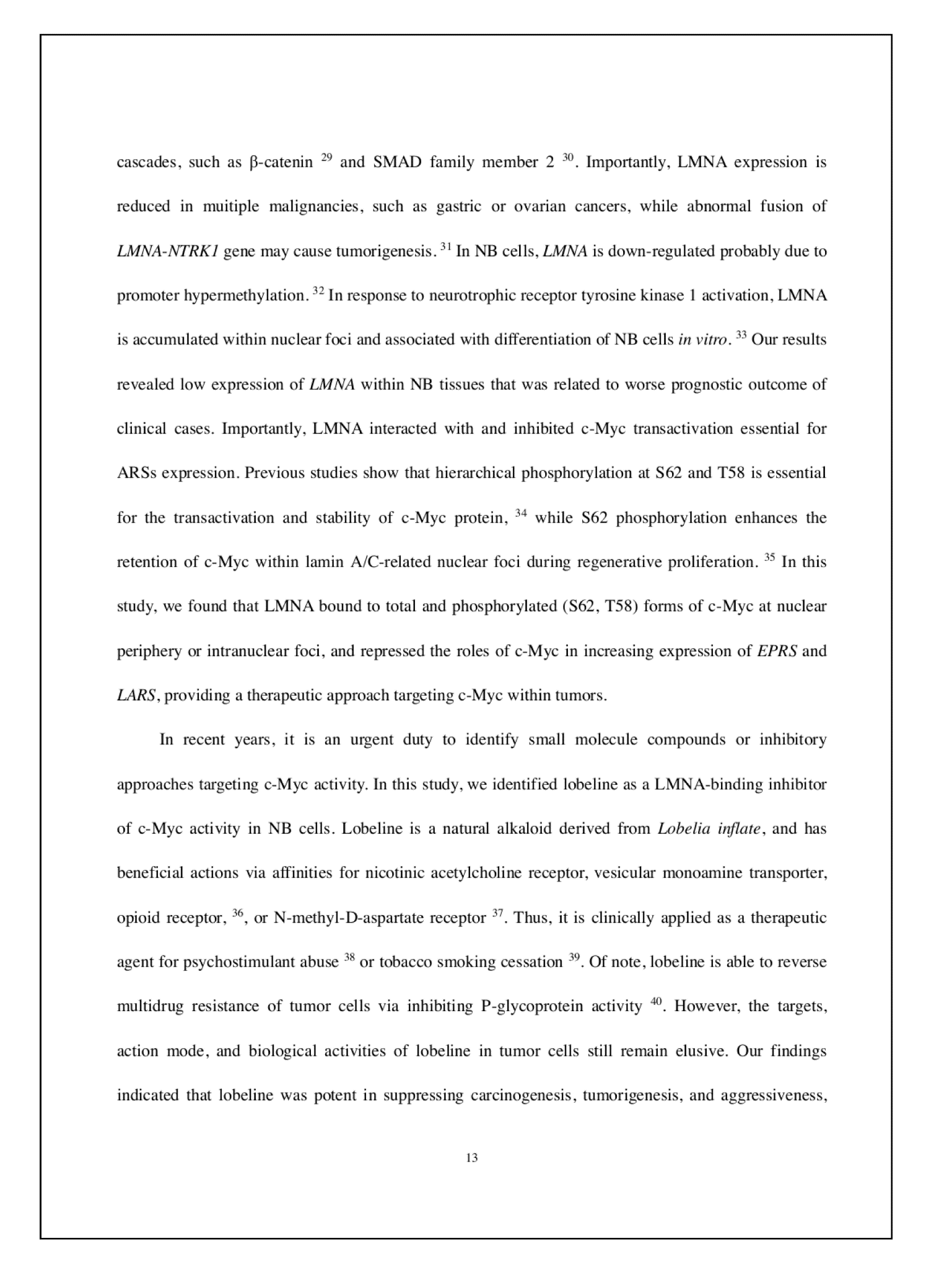


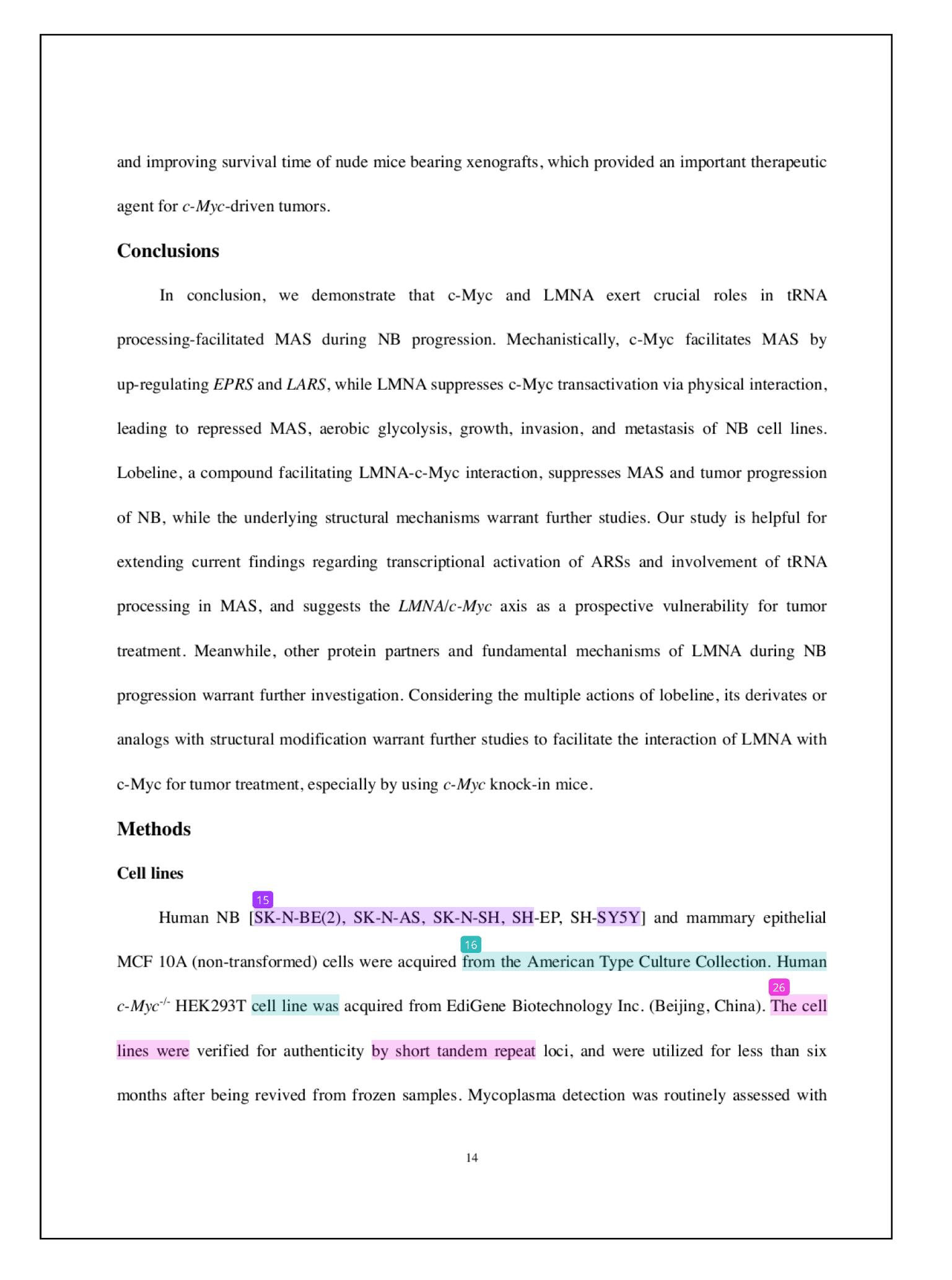


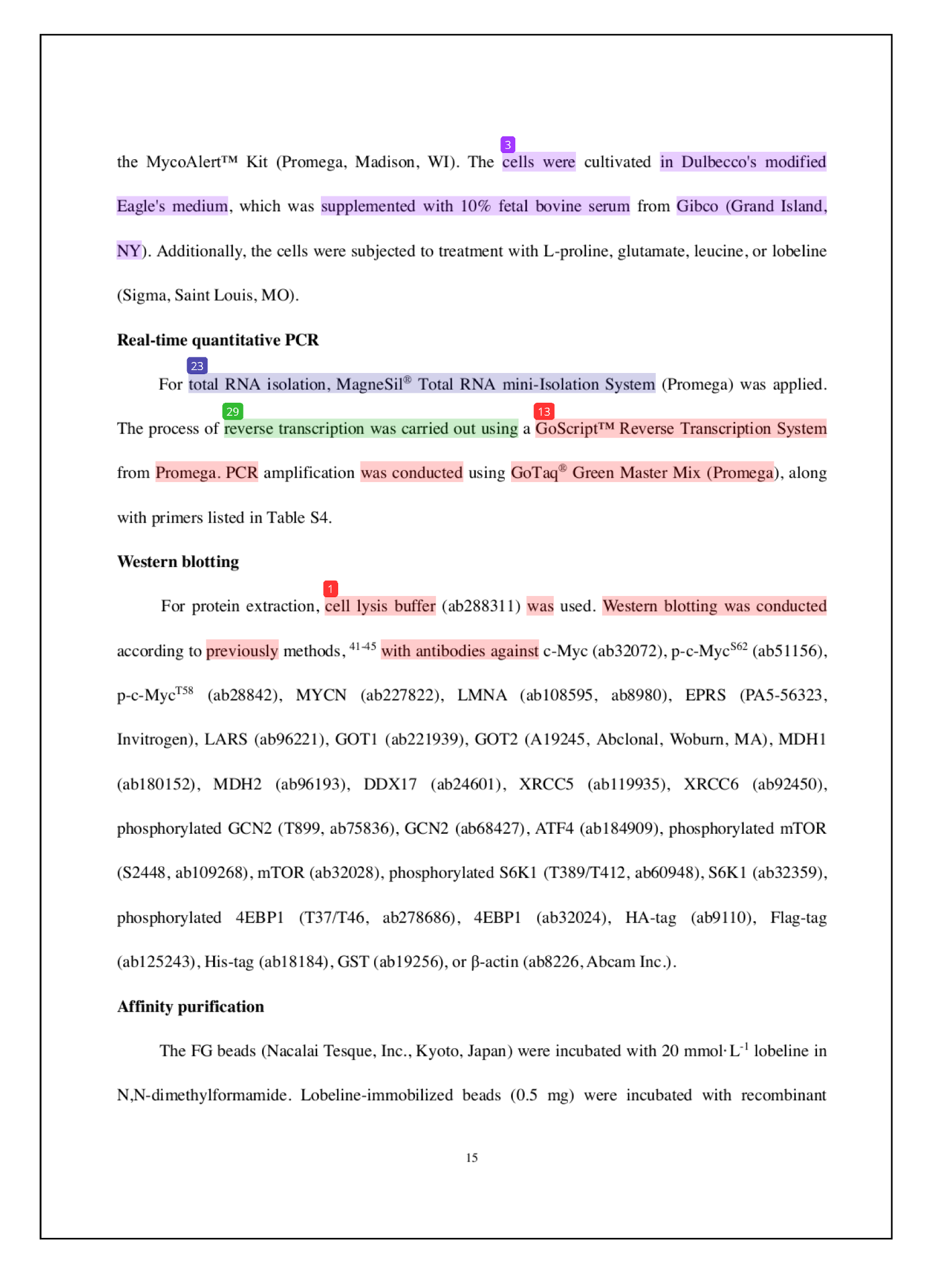


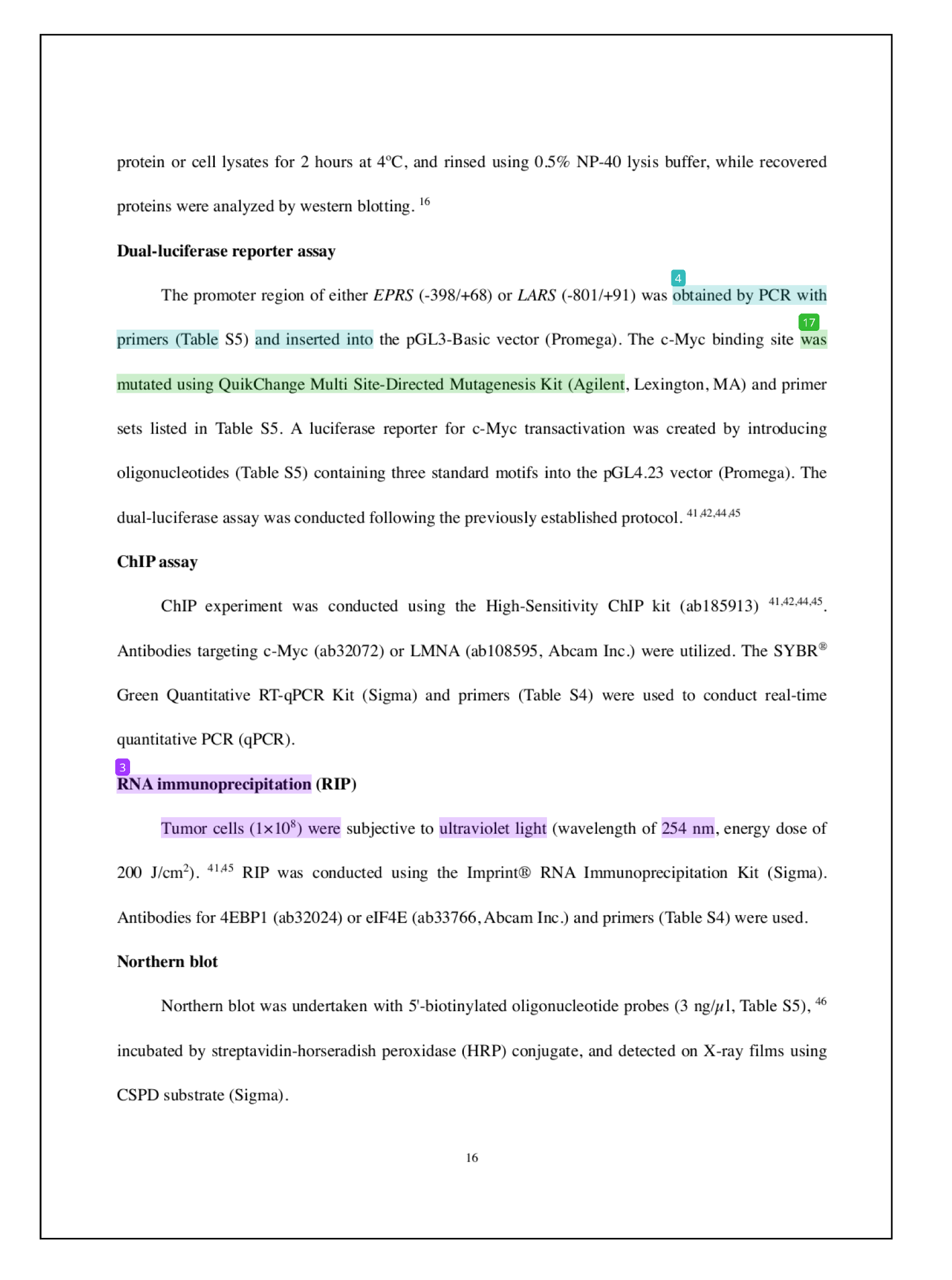


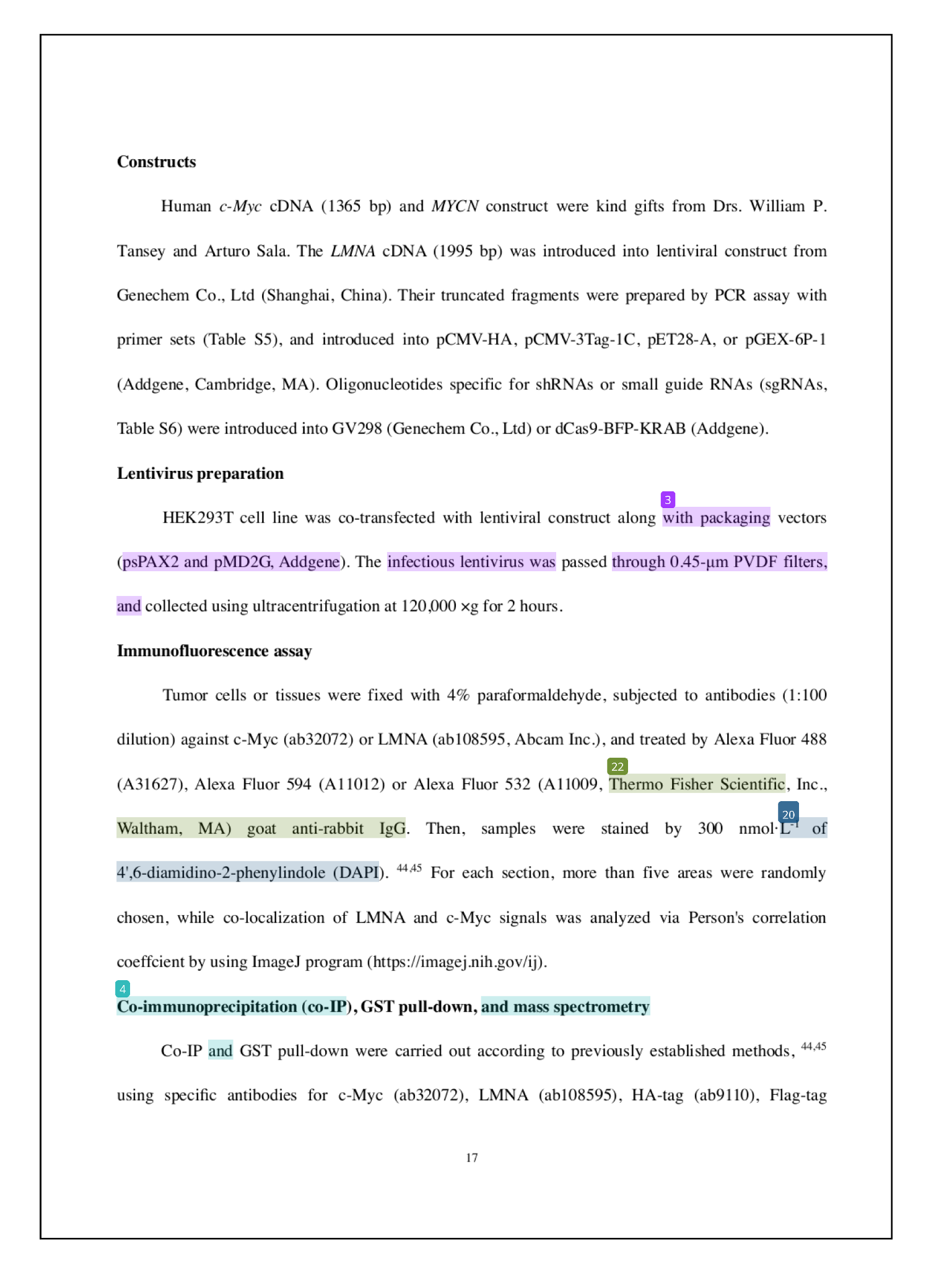


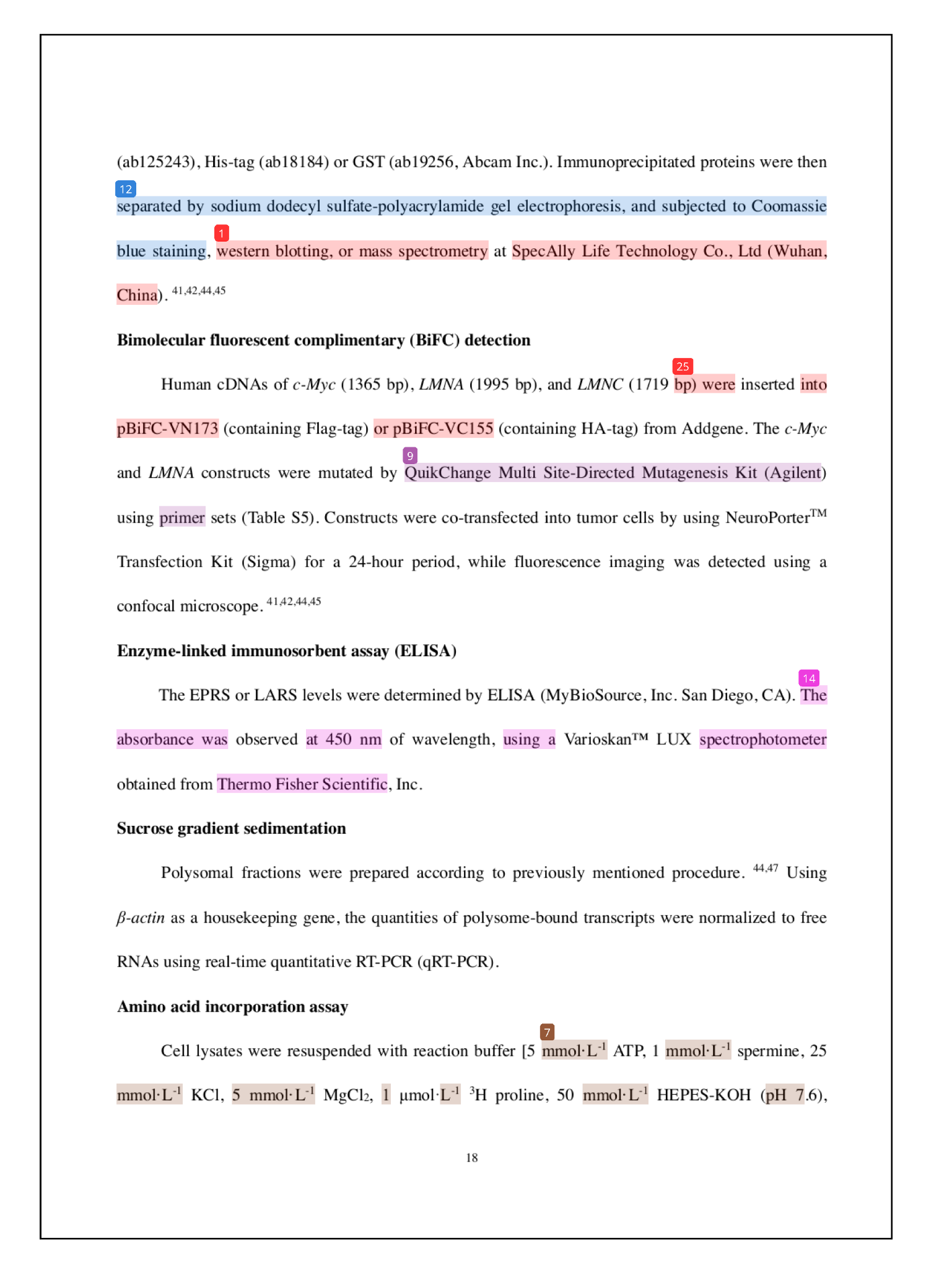


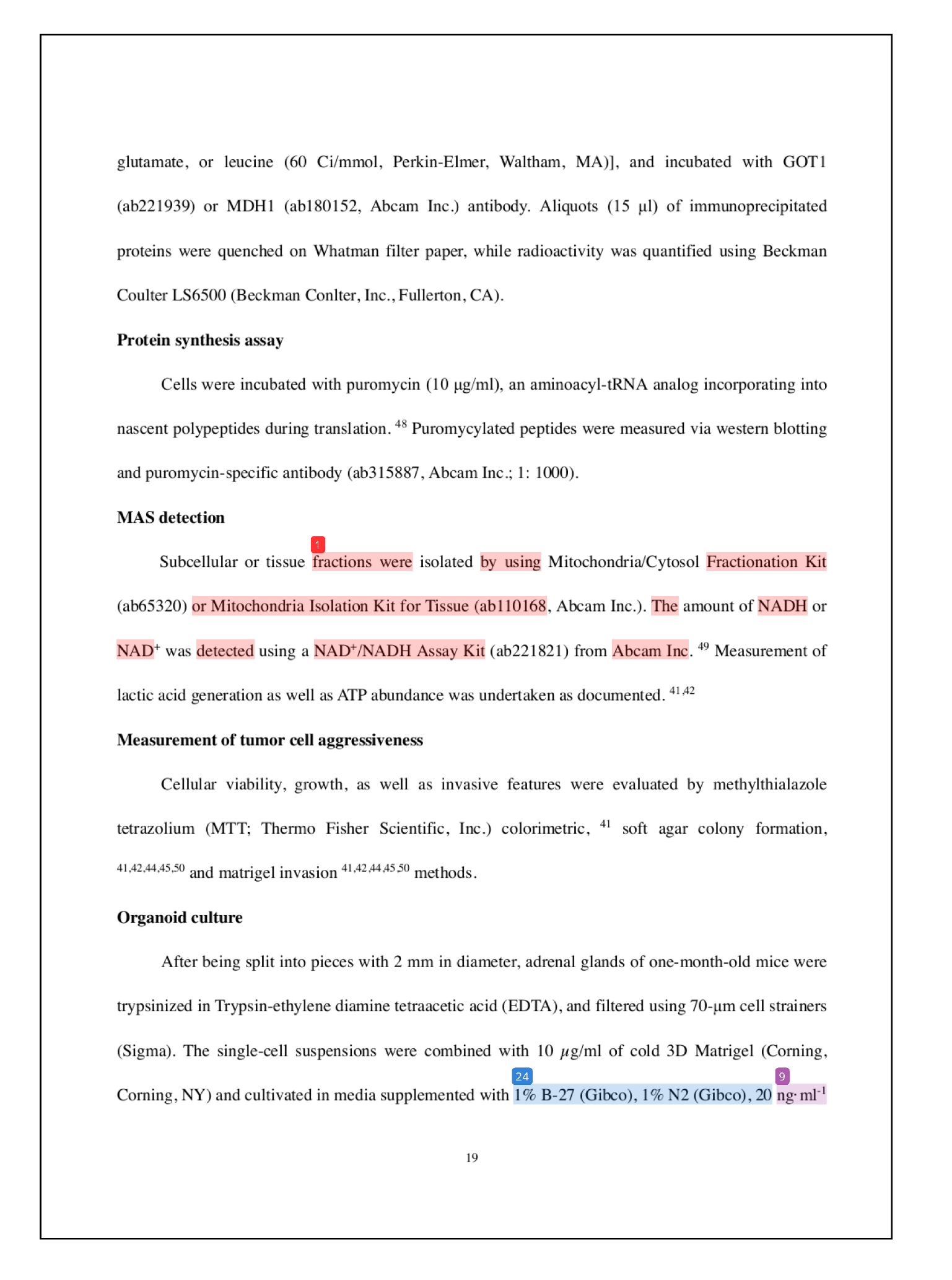


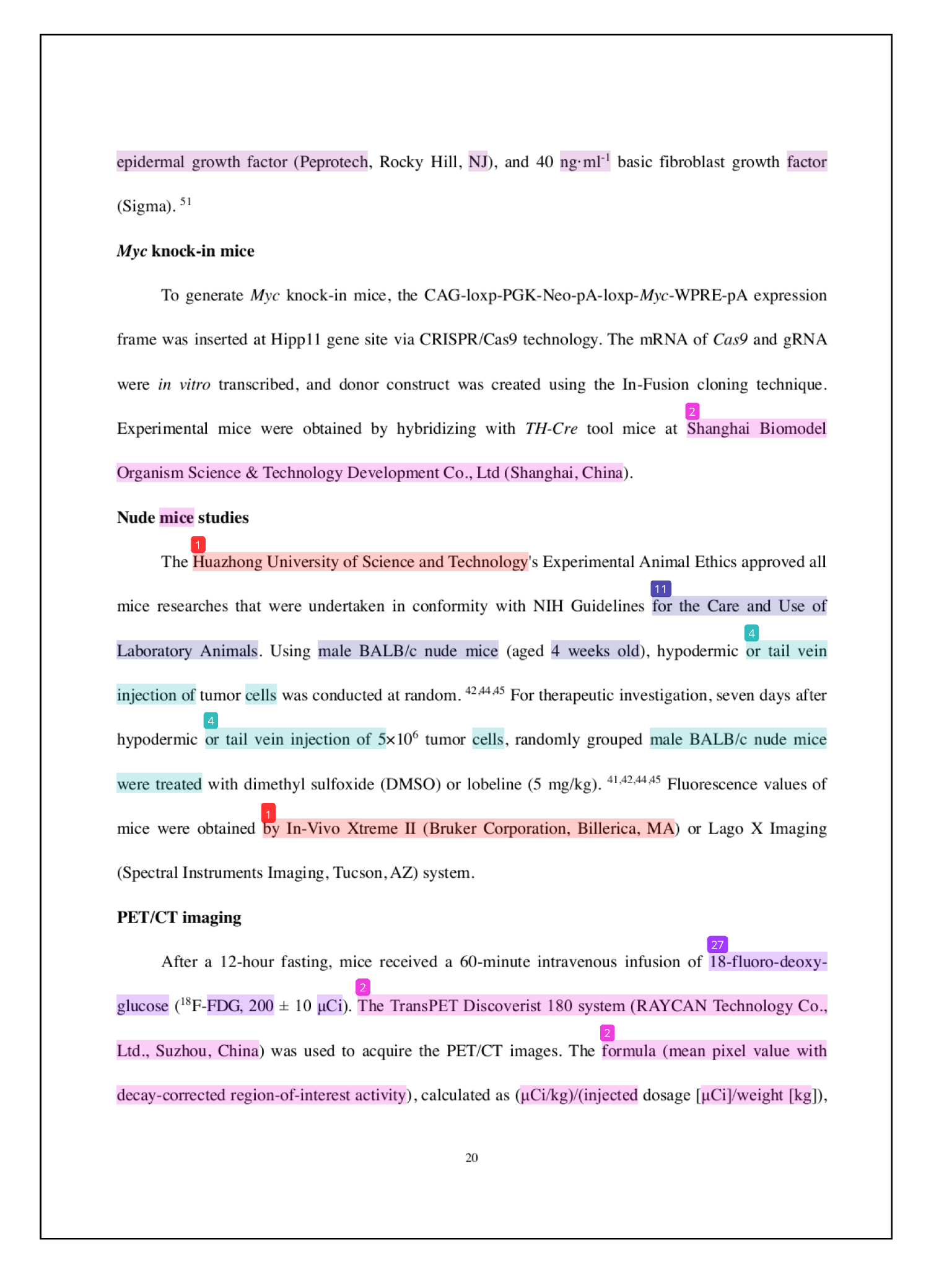


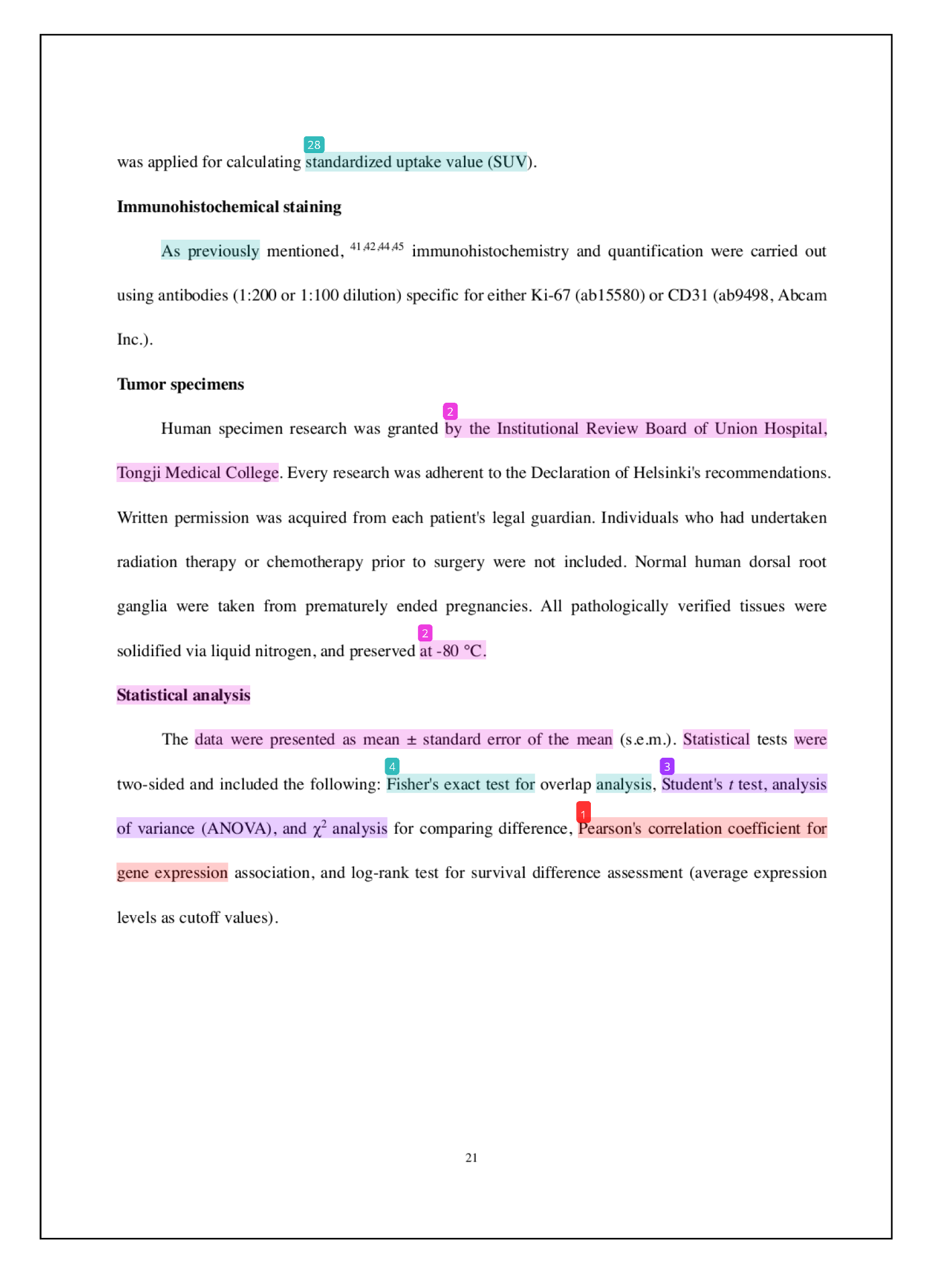


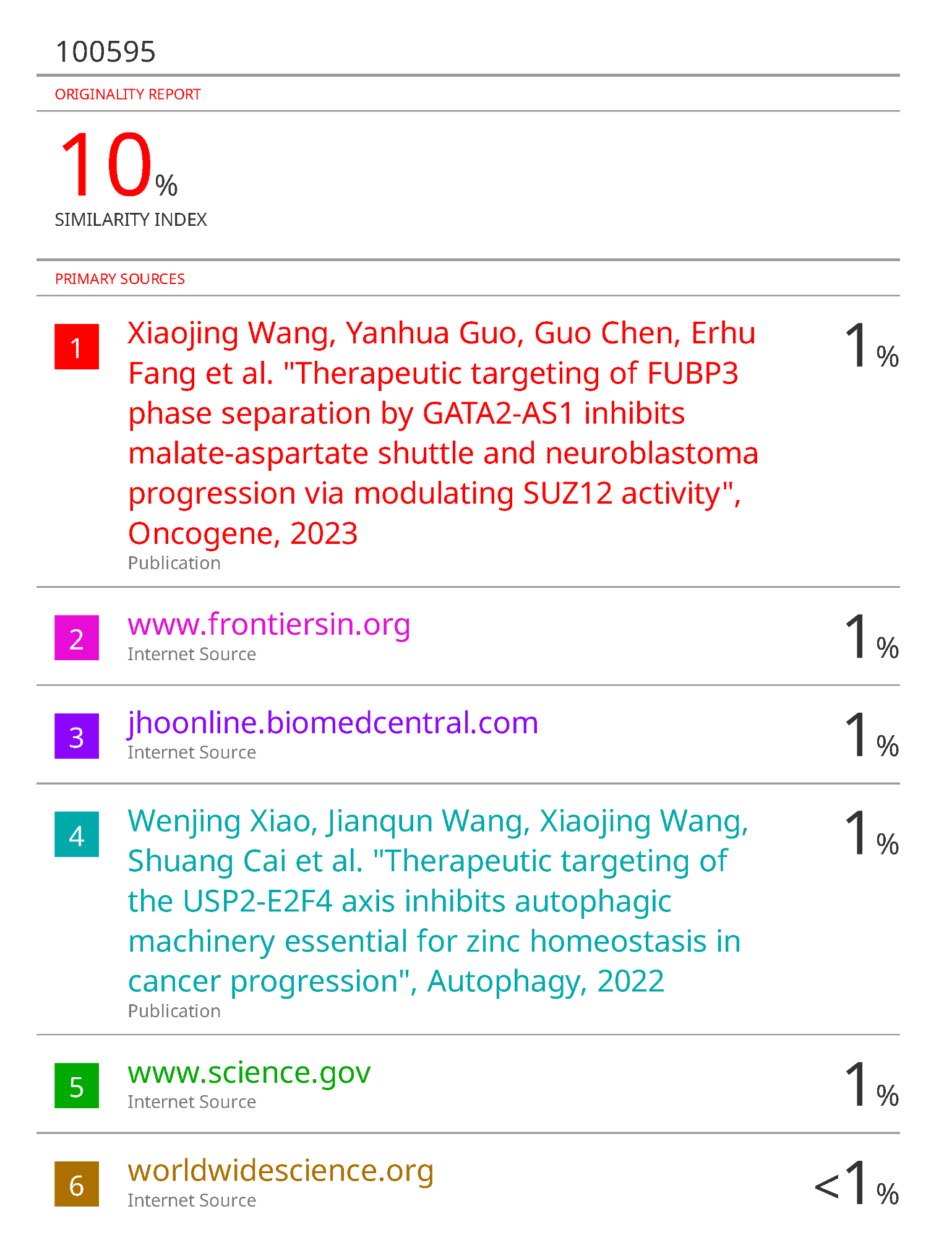


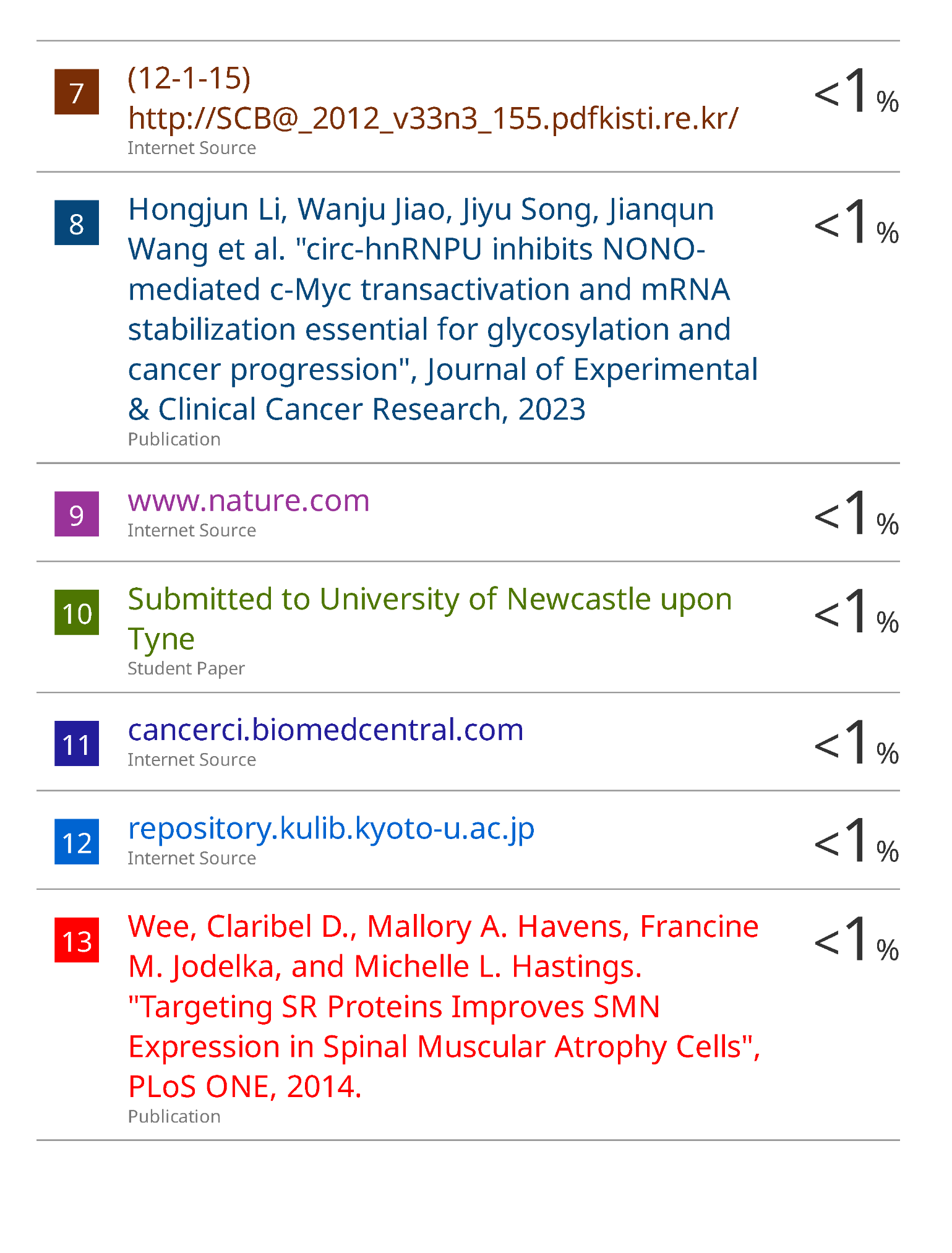


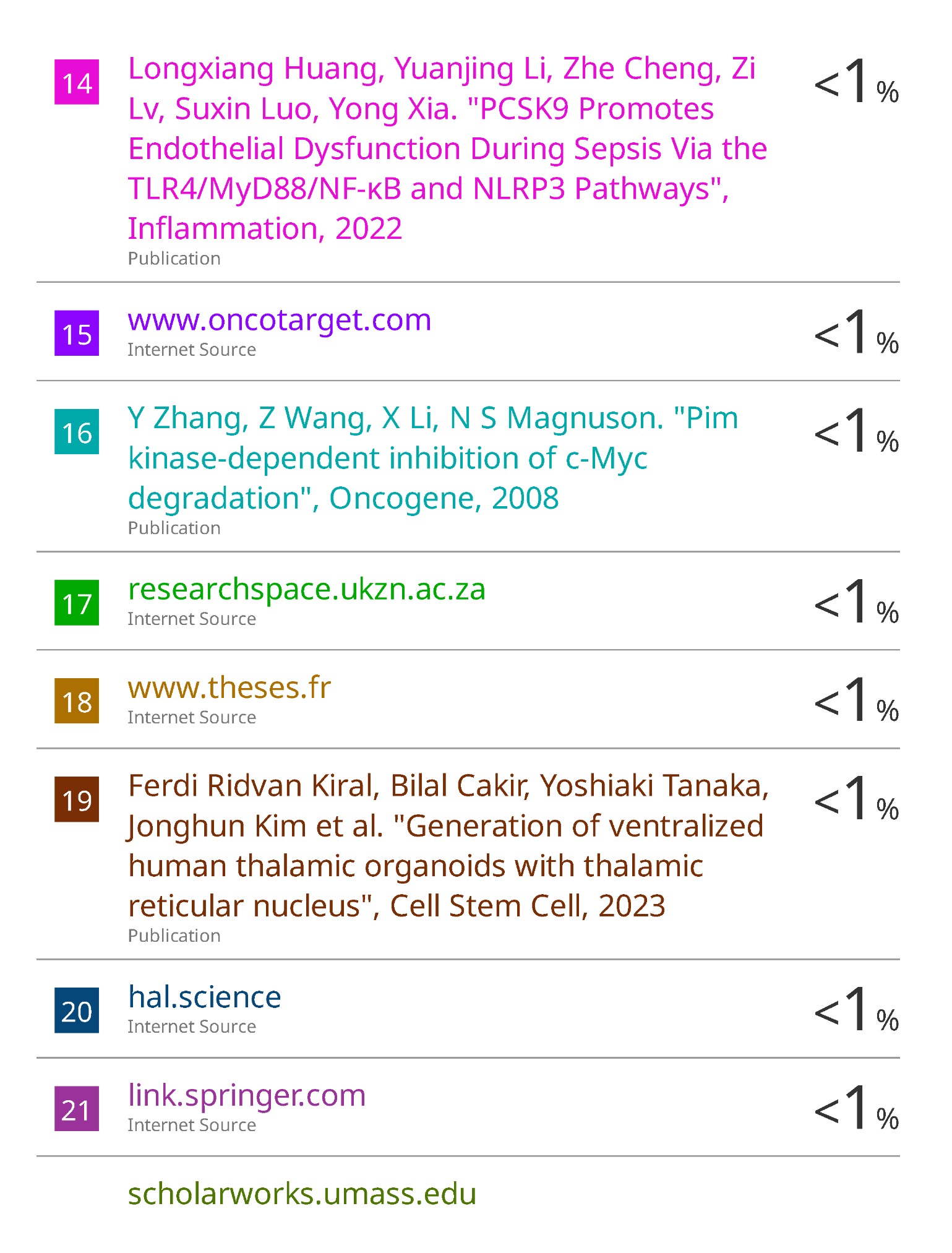


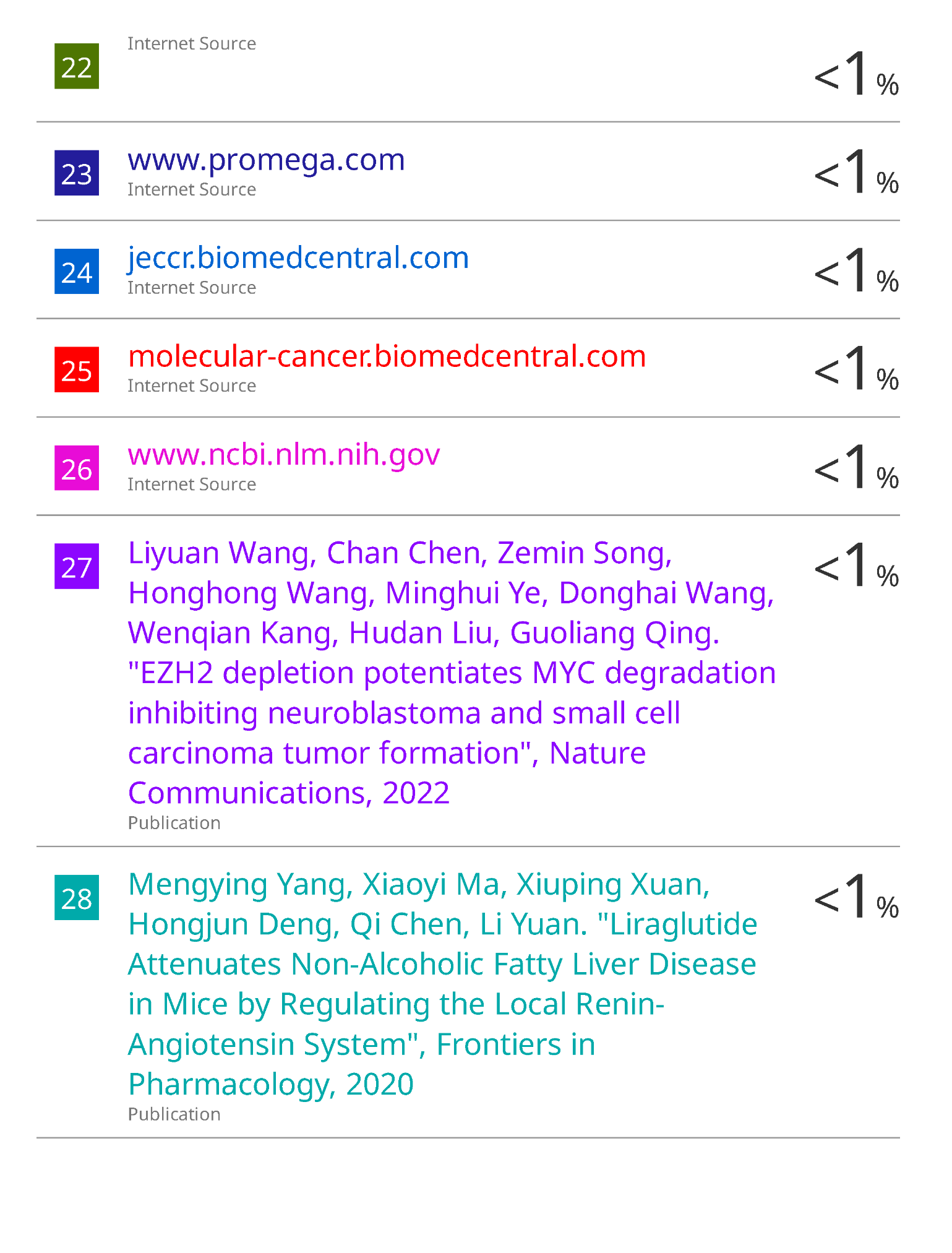


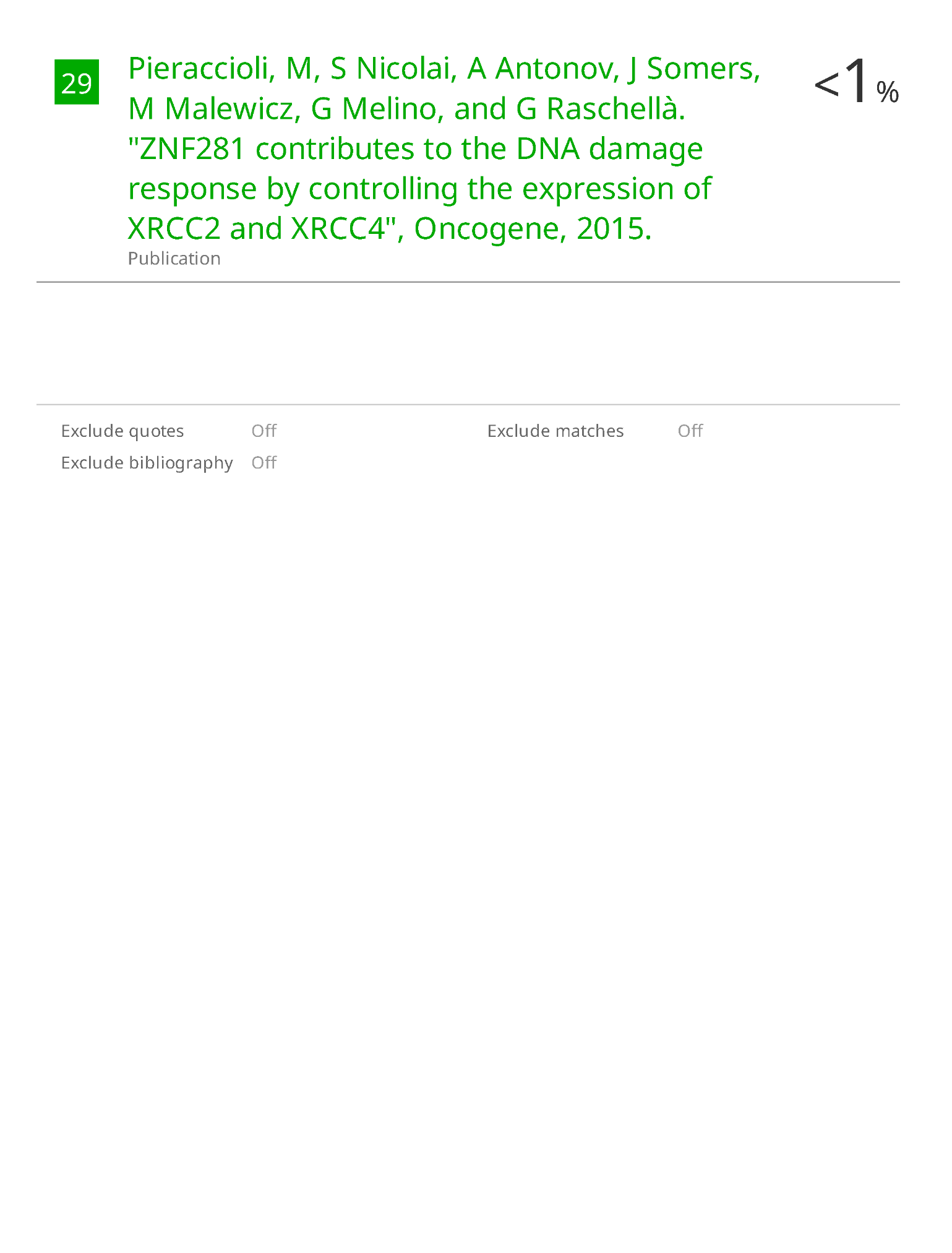

Supplement: Supplementary file 3 — Supporting Information [file CTM2-14-e1680-s003.docx]
